# Determining the Physiological Threshold for Angina (ORBITA-FIRE): A Double-Blind, Randomized, Placebo-Controlled Study

**DOI:** 10.1161/CIRCULATIONAHA.125.078738

**Published:** 2026-05-08

**Authors:** Fiyyaz Ahmed-Jushuf, Michael J. Foley, Shayna Chotai, Christopher A. Rajkumar, Danqi Wang, Florentina A. Simader, Krzysztof Macierzanka, Kayla Chiew, Sannidhya Misra, Rupert Williams, Klio Konstantinou, Jehangir N. Din, Shah R. Mohdnazri, Peter D. O’Kane, Peter Haworth, Sukhjinder S. Nijjer, Henry Seligman, Thomas R. Keeble, John R. Davies, Gerald Clesham, Jonathan Hinton, James C. Spratt, Jason N. Dungu, Daniel Knight, Tushar Kotecha, Frank E. Harrell, James P. Howard, Darrel P. Francis, Matthew J. Shun-Shin, Rasha K. Al-Lamee

**Affiliations:** National Heart and Lung Institute, Imperial College London, UK (F.A.-J., M.J.F., S.C., C.A.R., D.W., F.A.S., K.M., K.C., S.M., S.S.N., H.S., J.P.H., D.P.F., M.J.S.-S., R.K.A.-L.).; Imperial College Healthcare NHS Trust, London, UK (F.A.-J., M.J.F., S.C., C.A.R., K.C., S.M., S.S.N., H.S., J.P.H., D.P.F., M.J.S.-S., R.K.A.-L.).; St George’s, University of London and St. George’s University Hospitals NHS Foundation Trust, UK (R.W., J.C.S.).; Essex Cardiothoracic Centre, Mid and South Essex NHS Foundation Trust, Basildon, UK (K.K., S.R.M., T.R.K., J.R.D., G.C., J.N.D.).; Anglia Ruskin School of Medicine and MTRC, Anglia Ruskin University, Chelmsford, Essex, UK (T.R.K., J.R.D., J.N.D.).; University Hospitals Dorset NHS Foundation Trust, Bournemouth, UK (J.N.D., P.D.O., J.H.).; Portsmouth Hospitals University NHS Trust, UK (P.H.).; Royal Free London NHS Foundation Trust, UK (D.K., T.K.).; Institute of Cardiovascular Sciences, University College London, UK (D.K., T.K.).; Vanderbilt University School of Medicine, Nashville, TN (F.E.H.).

**Keywords:** angina pectoris, controlled clinical trials as topic, coronary artery disease, coronary circulation, fractional flow reserve, myocardial, ischemia, percutaneous coronary intervention

## Abstract

**BACKGROUND:**

In stable coronary artery disease, the primary goal of percutaneous coronary intervention (PCI) is symptom relief. Fractional flow reserve (FFR) and nonhyperemic pressure ratios such as resting full-cycle ratio (RFR) are used to guide revascularization. Although these indices correlate with myocardial ischemia, they have never been validated against the onset of angina. The physiological thresholds for angina, FFR_angina_ and RFR_angina_, at rest and during exercise remain undefined.

**METHODS:**

ORBITA-FIRE (Finding the Invasive Threshold for Symptom Relief in Exertional Angina) was a multicenter, double-blind, randomized, placebo-controlled study in patients with stable angina and single-vessel coronary artery disease. After imaging-guided PCI, an in-stent balloon was incrementally inflated until angina occurred at rest. This angina threshold was verified against placebo inflation, and corresponding FFR_angina_ and RFR_angina_ values were recorded at symptom onset. The protocol was repeated during low- and high-intensity exercise to assess changes in angina thresholds with increasing cardiac workload.

**RESULTS:**

Sixty-five patients were enrolled (mean age, 63.9±8.7 years; 74% male; 69% hypertensive; 23% diabetic; 91% with Canadian Cardiovascular Society class II–III angina). Median pre-PCI FFR was 0.59 (interquartile range [IQR], 0.46–0.70) and RFR was 0.61 (IQR, 0.40–0.82). Median FFR_angina_ at rest was 0.29 (IQR, 0.23–0.35), increasing to 0.38 (IQR, 0.30–0.48) during low-intensity exercise and 0.45 (IQR, 0.36–0.55) during high-intensity exercise. RFR_angina_ similarly increased from 0.22 (IQR, 0.16–0.30) at rest to 0.26 (IQR, 0.18–0.36) and 0.32 (IQR, 0.23–0.46) during low- and high-intensity exercise. All thresholds were significantly lower than clinical diagnostic cut points (*P*<0.001). Lower FFR_angina_ and RFR_angina_ thresholds were associated with greater symptom reproducibility across rest, low- and high-intensity exercise conditions (FFR_angina_: *P*=0.008, *P*<0.001, *P*<0.001, respectively; RFR_angina_: *P*=0.015, *P*<0.001, *P*=0.002, respectively). Lower angina thresholds across all conditions predicted higher baseline angina burden and greater symptom relief with PCI (probability of interaction >0.999).

**CONCLUSIONS:**

Physiological thresholds for angina, FFR_angina_ and RFR_angina_, are highly individualized, vary with cardiac workload, and are consistently lower than the universal ischemia-based thresholds used to guide revascularization. These findings support integrating personalized, symptom-linked physiology to refine patient selection and to improve symptomatic response to PCI.

**Clinical Perspective**

**What Is New?**

- This is the first double-blind, randomized, placebo-controlled study to define individualized physiological thresholds for angina onset, FFR_angina_ and RFR_angina_, in patients with stable single-vessel coronary artery disease.
- These symptom-linked thresholds were highly individualized, varied with cardiac workload, and were consistently lower than current clinical diagnostic cut points.
- Lower angina thresholds were associated with greater baseline symptom burden and more pronounced angina relief with percutaneous coronary intervention.

**What Are the Clinical Implications?**

- The wide variability in angina thresholds across patients and workloads indicates that fixed ischemia-based clinical cut points may not reliably predict symptom onset or the likelihood of symptom relief.
- Individualized angina thresholds, which link invasive pressure-derived physiology directly to symptom experience, may be more effective at identifying those most likely to benefit symptomatically from percutaneous coronary intervention.
- A fractional flow reserve ≤0.80 or nonhyperemic pressure ratio ≤0.89 should be interpreted with caution and within a broader physiological continuum that incorporates symptom burden, activity level, microvascular function, and pain perception. This approach supports more tailored, symptom-focused percutaneous coronary intervention decision-making and improves patient outcomes.

In stable coronary artery disease (CAD), the principal goal of percutaneous coronary intervention (PCI) is to relieve angina and improve quality of life.^1–5^ Both unblinded and placebo-controlled trials have demonstrated that PCI can deliver symptomatic benefit in appropriately selected patients.^1,4^ However, even after technically successful PCI with favorable postprocedural physiology, many patients remain symptomatic, highlighting a persistent disconnect between anatomical or physiological success and true symptom relief.^1,4^ Determining whether a coronary stenosis is directly responsible for angina, and thereby identifying which patients are most likely to benefit from PCI, remains a fundamental and unresolved challenge in interventional cardiology.^5,6^

Current guidelines give a Class I (Level A) recommendation for ischemia-guided PCI using hyperemic pressure-derived indices, such as fractional flow reserve (FFR; ≤0.80), and nonhyperemic pressure ratios, such as resting full-cycle ratio (RFR; ≤0.89).^7,8^ These thresholds are well validated for detecting flow-limiting stenoses and stratifying cardiovascular risk,^9–11^ but their ability to identify patients who will experience symptomatic benefit from PCI has not been established in clinical trials.^12^ Symptom improvement appears greatest in patients with the largest post-PCI increase in FFR,^13^ and blinded data suggest that those with the lowest baseline FFR and instantaneous wave-free ratio derive the greatest angina relief with PCI.^14^ However, the direct relationship between angina and invasive physiology has never been investigated under placebo-controlled conditions.

A growing body of evidence demonstrates that ischemia and symptoms are frequently dissociated. Many patients with objective ischemia report no symptoms, whereas others without demonstrable ischemia remain highly symptomatic.^1,5,15–18^ This heterogeneity likely reflects a complex interplay of patient-specific factors, including diabetic status, microvascular dysfunction, autonomic tone, myocardial mass, endothelial function, psychological state, cardiac interoception, and individual pain perception.^16,19–24^

Moreover, the use of a binary physiological threshold assumes a universal cut point for angina onset across all patients, a premise that is unlikely to be biologically plausible.^25^ Fixed cut points do not account for substantial interindividual variability in physiological response.^26^ While early studies established FFR <0.75 as highly specific for ischemia, subsequent trials raised this threshold to ≤0.80 to improve diagnostic sensitivity rather than to predict symptom response.^3,11,12,27^ These thresholds therefore represent population averages rather than patient-level measures. Even among patients with identical FFR values, angina burden can vary markedly.^5^ Individualized hemodynamic variation, differences in coronary flow reserve, collateral circulation, and other physiological modifiers also complicate interpretation, limiting the utility of a one-size-fits-all threshold.^28–30^

Interpretation is further challenged by the fact that invasive physiological assessments are typically performed in a resting, nonexercise state in the cardiac catheterization laboratory.^10,11^ Clinical angina, by contrast, often arises under very different conditions, typically during exertion. While hyperemic agents induce maximal myocardial blood flow, real-world exercise generates additional changes in coronary hemodynamics, including coronary vasodilation, altered microvascular resistance, increased myocardial oxygen demand, and reduced systemic vascular resistance, none of which are fully replicated with pharmacological hyperemia.^31,32^ Consequently, physiological indices obtained at rest may not reliably predict exertional symptoms.

ORBITA-FIRE (Finding the Invasive Threshold for Symptom Relief in Exertional Angina) was a double-blind, randomized, placebo-controlled study, designed to define the physiological threshold for angina onset for each patient at rest and during exercise. These individualized, symptom-linked thresholds were correlated with invasive pressure indices to derive FFR_angina_ and RFR_angina_. The study also tested whether established universal ischemic cut points (FFR≤0.80, RFR≤0.89) predict angina onset, or whether individualized, angina-specific physiological thresholds (FFR_angina_, RFR_angina_) provide greater accuracy. Last, it explored the association between these thresholds and the magnitude of angina relief after PCI.

## Methods

### Study Design

This multicenter, double-blind, randomized, placebo-controlled study was conducted across 6 sites in the United Kingdom. A complete list of trial centers is provided in the Supplemental Appendix. The study was approved by the London Central Research Ethics Committee (22/LO/0308), and all patients provided written informed consent before enrollment. Study data, analytical methods, and materials are not available for independent reproduction of the results or replication of methodology.

### Patient Eligibility

Eligible patients were ≥18 years of age with exertional angina, severe single-vessel CAD, and confirmed evidence of ischemia. Patients reporting only angina-equivalent symptoms, such as dyspnea, in the absence of chest pain were excluded. Additional exclusion criteria included multivessel disease, anatomy unsuitable for PCI, or absence of ischemia on invasive or noninvasive testing; the full list of exclusions is provided in the Supplemental Appendix.

### Enrollment and Pre-PCI Symptom Assessment

At enrollment, all antianginal medications were discontinued, and patients entered a 2-week symptom assessment phase. During this period, daily angina episodes were recorded with the ORBITA application (app; Figure S2).^33^ Baseline heart rate and physical activity were monitored with a smartwatch provided to each participant. Patients who remained asymptomatic despite stopping antianginal therapy were excluded.

Pre-PCI assessments included grading of angina severity according to the Canadian Cardiovascular Society classification system and evaluations of symptoms and quality of life with the Seattle Angina Questionnaire.^34^ Angina typicality was assessed with the Rose Angina Questionnaire, and pain sensitivity was measured with the Pain Sensitivity Questionnaire.^35,36^

### Invasive Protocol

**Figure 1.**
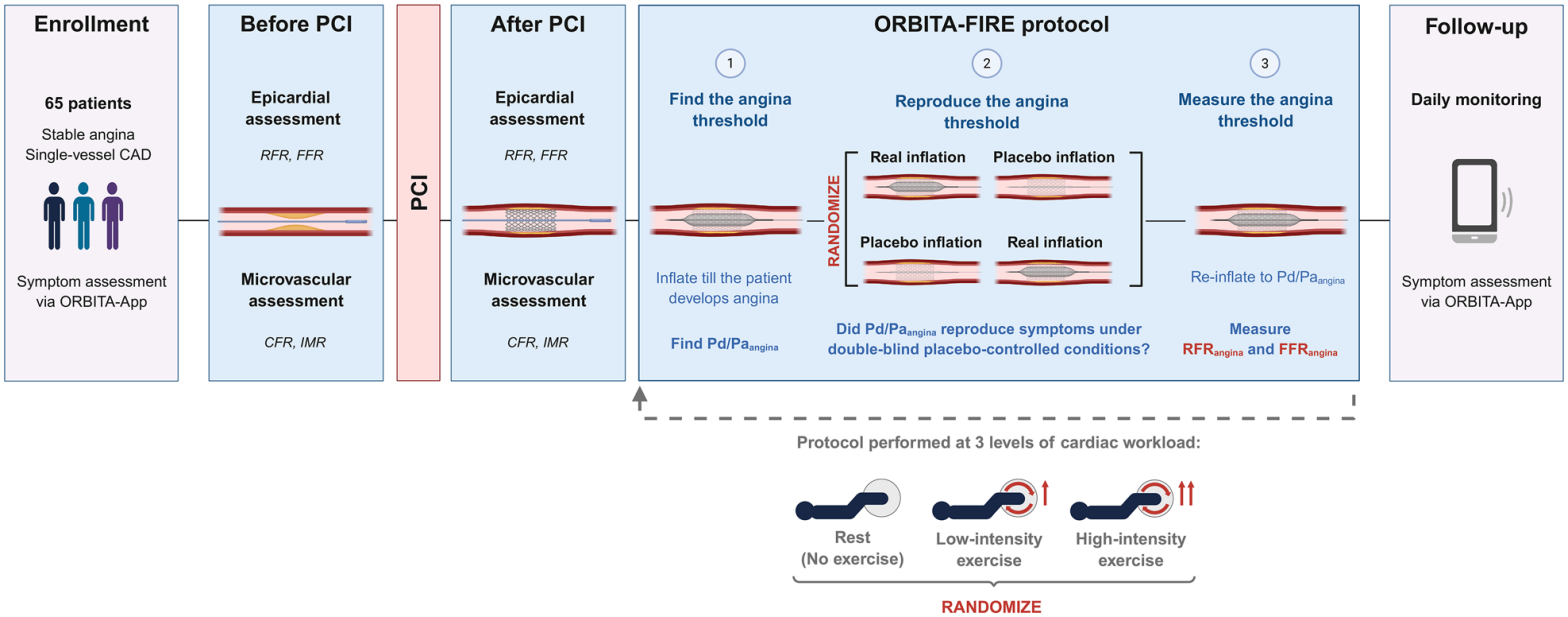
**ORBITA-FIRE study design.** At enrollment, all patients discontinued antianginal medications and completed baseline symptom questionnaires followed by a 2-week preprocedural symptom assessment phase recorded with the symptom application (app). Patients then underwent invasive assessment, including pre–percutaneous coronary intervention (PCI) and post-PCI coronary physiology followed by the main study protocol to determine individualized fractional flow reserve (FFR) at the point of angina (FFR_angina_) and resting full-cycle ratio (RFR) at the point of angina (RFR_angina_) thresholds at rest and during exercise under double-blind placebo-controlled conditions. After the procedure, patients entered a 12-week unblinded follow-up phase, with ongoing symptom burden recorded with the symptom-tracking app. CAD indicates coronary artery disease; CFR, coronary flow reserve; IMR, index of microvascular resistance; ORBITA-FIRE, Finding the Invasive Threshold for Symptom Relief in Exertional Angina; and Pd/Pa, distal to aortic pressure ratio.

The invasive study protocol is visualized in Figure 1.

### Preprocedural Exercise Protocol

Before PCI, all patients received dual antiplatelet therapy. Patients were positioned on the catheterization table with their legs secured to a supine bicycle ergometer (Lode Angio Imaging). Opiates and anxiolytics were not routinely used to ensure unrestricted exercise performance and accurate symptom reporting. Where required, midazolam 1 mg IV with a maximum total dose of 4 mg was administered.

After 6-Fr radial access was obtained, patients underwent a 2-minute familiarization period on the ergometer. The exercise protocol then began at 40 rpm with an initial workload of 40 W, increasing by 20 W every minute until either angina occurred or the patient reached exhaustion. This stage established symptom characteristics and the peak heart rate associated with angina, which would be used later in the research protocol.

### Coronary Angiography and Invasive Physiology

All patients underwent coronary angiography followed by baseline physiological measurements after administration of intracoronary nitroglycerin (400 μg) to ensure epicardial stability. A dual pressure and temperature sensor–tipped 0.014-in intracoronary guidewire (PressureWire X, Abbott Inc) was used to assess epicardial pressure gradients, coronary flow, and microvascular resistance. Hemodynamic and thermodilution indices were derived with the CoroFlow software (Coroventis Research, Uppsala, Sweden). The pressure sensor was zeroed in the aorta before being advanced at least 3 vessel diameters distal to the target lesion. Physiological assessments included distal-to-proximal aortic pressure ratio (Pd/Pa), RFR, FFR, coronary flow reserve, and index of microvascular resistance (Table S2).^10,11,37–42^ Stable hyperemia was induced with an intravenous adenosine infusion administered at 140 μg·kg^−1^·min^−1^ through a large-bore peripheral cannula.^11^

### PCI of Target Vessel

All patients with confirmed severe single-vessel CAD and objective evidence of ischemia underwent PCI of the target vessel. Stent optimization with intravascular imaging was mandatory. The choice of imaging modality was at the operator’s discretion. Physiological measurements were repeated after PCI.

### Angina Threshold Assessment

After physiological assessment, individualized angina thresholds were determined under 3 conditions: rest (without exercise), low-intensity exercise, and high-intensity exercise in randomized order.

### Rest Angina Threshold

At rest, without exercise, a noncompliant angioplasty balloon, sized 0.5 mm smaller than the external elastic lamina diameter of the reference vessel measured by intravascular imaging, was positioned within the stented segment. Incremental low-pressure inflations were applied every 5 to 10 seconds with a digital indeflator (Blue Diamond Inflation Device, Merit Medical) to induce a coronary stenosis. Each incremental balloon inflation lowered the Pd/Pa by ≈0.02. At the first onset of chest pain, the rest angina inflation pressure Pd/Pa was recorded, and the balloon was deflated. Experimental symptom intensity (0–10) and similarity (0–10) were scored relative to the patient’s typical day-to-day symptoms.

A double-blind, randomized, placebo-controlled protocol was used to validate the angina threshold and minimize reporting bias. Each patient underwent 2 paired inflations in randomized order: 1 real, flow-limiting balloon inflation performed at the same Pd/Pa previously shown to provoke angina, and 1 placebo inflation. During placebo inflation, the balloon remained deflated in the guide catheter, and identical visual and auditory cues were used to simulate a real inflation. Both the patient and the research team member who recorded symptom onset were blinded to inflation type and physiological measurements. After completion of both real and placebo inflations, patients reported symptoms and were asked which inflation they believed was real. There were no crossovers.

To determine FFR_angina_ and RFR_angina_ in a nonexercise resting state, the intracoronary balloon was inflated to replicate the Pd/Pa corresponding to the rest angina threshold. Nonhyperemic RFR_angina_ was then measured. Hyperemia was then induced with adenosine, and FFR_angina_ was measured. Last, the adenosine infusion was stopped, the balloon was deflated, and hemodynamics were allowed to return to baseline.

### Low-Intensity Exercise Angina Threshold

During low-intensity exercise, the guide catheter was disengaged after the balloon was positioned in stent to avoid vessel trauma during exercise and allow undamped aortic pressure measurement. Patients began cycling at 40 rpm with an initial workload of 25 W, increasing to 50 W after 1 minute. At 50 W, incremental balloon inflation was performed every 5 to 10 seconds to lower the Pd/Pa by ≈0.02 with each increment (Figures S3 and S4). At the first onset of chest pain, the low-intensity exercise angina inflation pressure Pd/Pa was recorded, and the balloon was deflated. Experimental symptom intensity (0–10) and similarity (0–10) were scored relative to the patient’s typical day-to-day symptoms. The double-blind, randomized, placebo-controlled symptom verification protocol described above was repeated during steady-state low-intensity exercise at 50 W.

To determine RFR_angina_ and FFR_angina_ in a low-intensity exercise state, the Pd/Pa recorded at the point of low-intensity exercise angina was replicated on exercise. With the balloon remaining inflated, exercise was stopped to allow the heart rate to return to baseline. Nonhyperemic RFR_angina_ was then measured. Hyperemia was then induced with adenosine, and FFR_angina_ was measured. Last, the adenosine infusion was stopped, the balloon was deflated, and hemodynamics were allowed to return to baseline. Because this stage was introduced after feasibility of the exercise protocol had been demonstrated in the first 4 patients, low-intensity exercise data were not collected in these individuals but were obtained in all subsequent patients to capture lower-workload symptom physiology.

### High-Intensity Exercise Angina Threshold

The protocol was repeated at a higher workload to define the high-intensity exercise angina threshold. For the higher workload, patients began cycling at 40 rpm at an initial workload of 40 W, increasing by 20 W/min until they achieved the peak heart rate previously triggering angina during baseline assessment. At this peak heart rate, the balloon was incrementally inflated to identify the angina threshold.

### Follow-Up

After completing the invasive protocol, patients entered a 12-week unblinded follow-up phase. Daily symptom reporting continued through the ORBITA app. Initiation and adjustment of antianginal therapy were guided by patient-reported symptoms and managed by the study team.

### Randomization

Randomization occurred at 2 stages in the protocol: to determine the order of rest and exercise assessments and determine the sequence of real versus placebo inflations. Randomization sequences were generated with RANDI-2 software.

### Statistical Analysis

The published reproducibility of FFR measurements informed sample size and power calculations (Supplemental Appendix). Continuous variables are expressed as mean±SD or median (interquartile range [IQR]). Categorical variables are expressed as frequencies and percentages. Physiological thresholds for angina, including FFR_angina_ and RFR_angina_, were summarized as medians (IQRs) under resting, low-intensity, and high-intensity exercise conditions. Comparisons of angina thresholds with established clinical cut points (FFR ≤0.80, RFR ≤0.89) were performed with the Wilcoxon signed-rank test (2-sided significance level of *P*<0.05). Within-patient comparisons of physiological angina thresholds across different workload states (rest, low-intensity exercise, and high intensity-exercise) were performed with the Friedman test. The association between angina thresholds (FFR_angina_ and RFR_angina_) and angina reproducibility (binary outcome: reproducible versus nonreproducible angina on repeat exposure to the same physiological stimulus) was assessed with logistic regression.

Bayesian ordinal regression models were used to evaluate the relationship between angina frequency and the unblinded effect of PCI, as recorded by the ORBITA app before PCI and during follow-up.^4,43^ In these models, follow-up scores were conditioned on pre-PCI values, with an interaction term for FFR_angina_ and RFR_angina_. Nonlinearity was addressed with restricted cubic splines with 3 knots for continuous predictors. Treatment efficacy was compared between patients in the upper and lower quartiles of FFR_angina_ and RFR_angina_ across different levels of cardiac workload. Posterior probabilities were then estimated to quantify the likelihood that lower FFR_angina_ and RFR_angina_ values were associated with greater symptomatic benefit from PCI. In the proportional odds framework, odds ratios <1 indicate lower odds of being in a higher angina-frequency category after PCI per unit decrease in physiological threshold (FFR_angina_ and RFR_angina_), consistent with greater symptomatic improvement among those with lower thresholds (Supplemental Appendix).

All analyses were performed in R (R Foundation for Statistical Computing, Vienna, Austria) with the rmsb package for regression modeling^43,44^ and other base and contributed packages as appropriate.

## Results

Between September 12, 2022, and March 11, 2025, a total of 65 patients completed the study protocol. A CONSORT (Consolidated Standards of Reporting Trials) flow diagram is available in the Supplemental Appendix (Figure S1).

### Patient Demographics

The mean age was 63.9±8.7 years, and 48 (73.8%) were male. Most patients (n=59, 90.8%) had Canadian Cardiovascular Society class II or III angina, and 51 patients (78.5%) had Rose angina. The median angina duration before enrollment was 36 weeks (IQR, 26–53 weeks). Most patients (n=55, 84.6%) were on at least 1 antianginal medication before entering the study (Tables S18 and S19). Baseline demographic and clinical characteristics are summarized in Table 1.

**Table 1.**
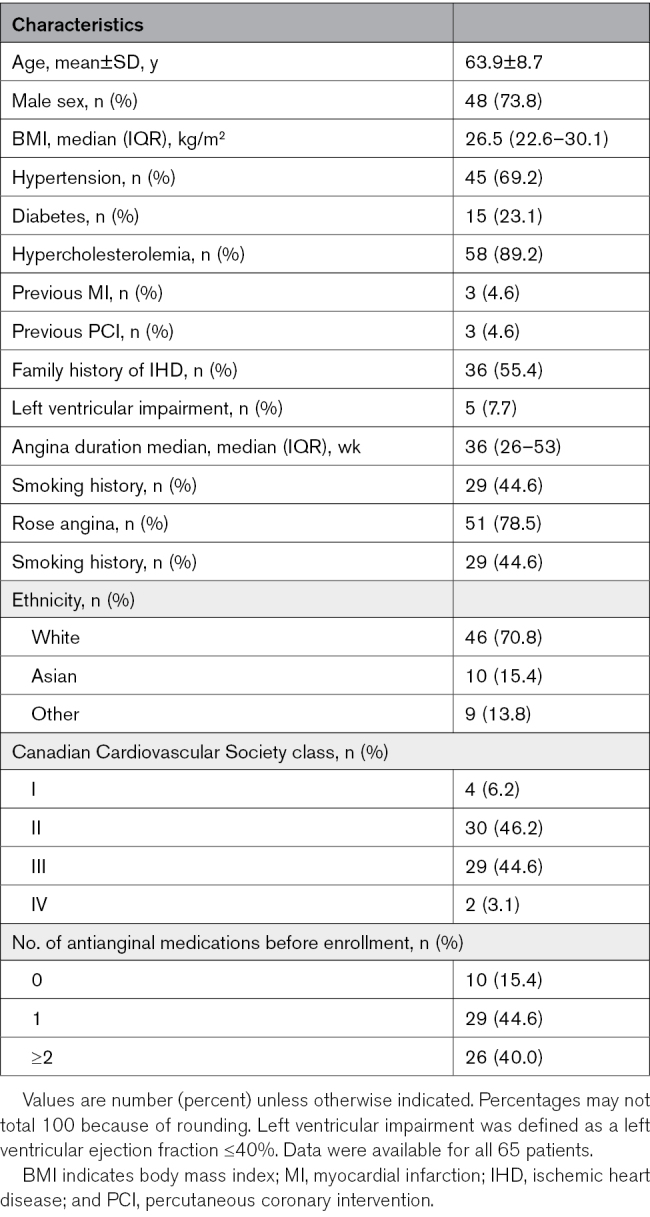
Baseline Characteristics at Enrollment

### Procedural Characteristics

The most frequently assessed vessel was the left anterior descending artery (LAD), which accounted for 47 patients (72.3%), followed by the right coronary artery in 12 (18.5%) and any other coronary territory in 6 (9.2%). Median pre-PCI physiological indices were as follows: FFR of 0.59 (IQR, 0.46–0.70), RFR of 0.61 (IQR, 0.40–0.82), coronary flow reserve of 1.7 (IQR, 1.1–2.5), and index of microvascular resistance of 29 (IQR, 18–39). After PCI, physiological indices improved across all parameters: median post-PCI FFR increased to 0.88 (IQR, 0.84–0.92), RFR to 0.92 (IQR, 0.90–0.94), and coronary flow reserve to 3.8 (IQR, 2.2–5.1), and index of microvascular resistance decreased to 20 (IQR, 12–32). Pre-PCI and post-PCI invasive physiological data are presented in Table 2.

**Table 2.**
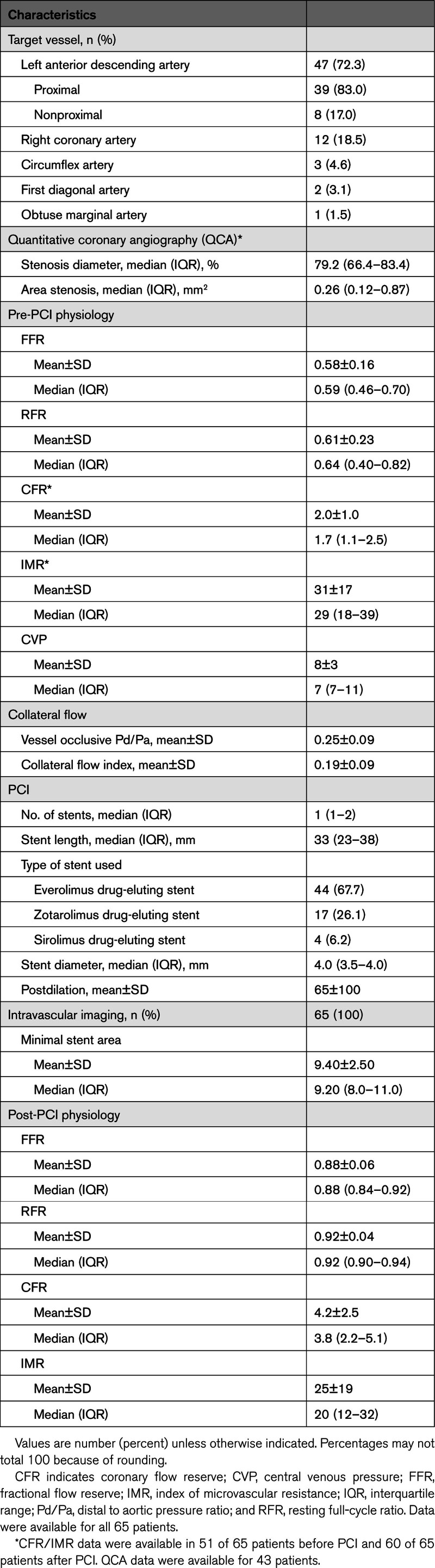
Procedural Characteristics

### Study End Points

After successful PCI with normalized physiology, individualized angina thresholds were determined at rest (without exercise) and during low-intensity and high-intensity exercise. Angina thresholds and related physiological measurements are summarized in Table 3.

**Table 3.**
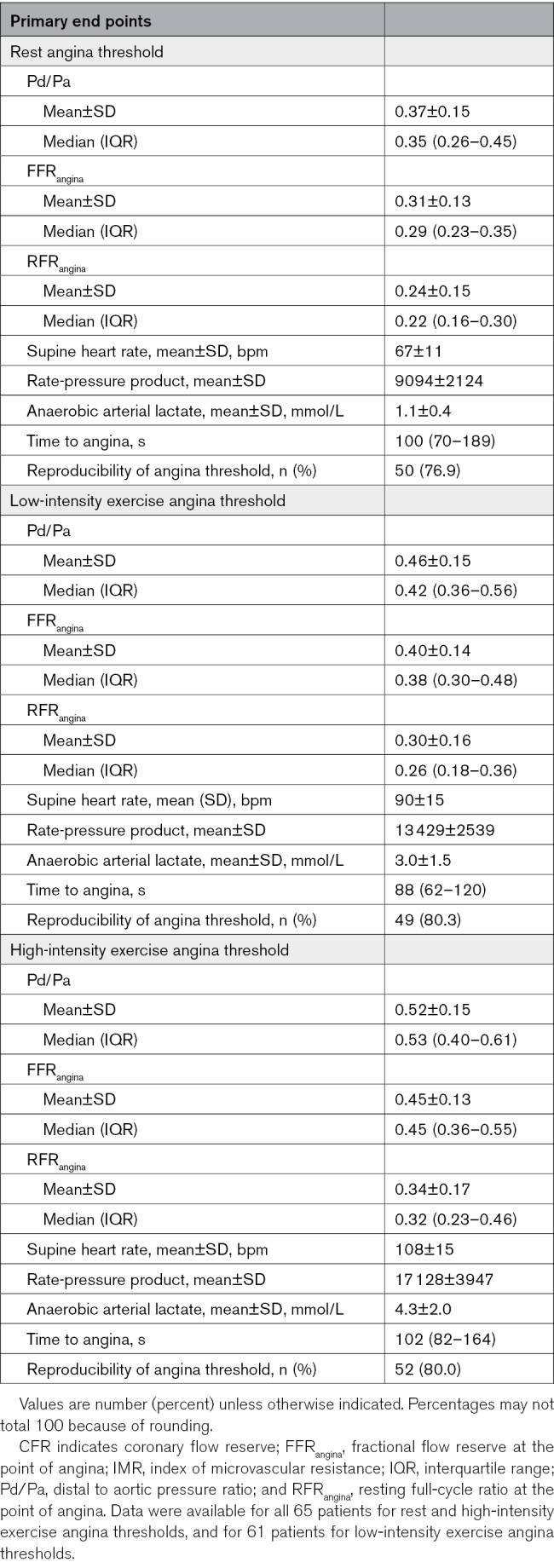
Primary and Secondary End Points

### Rest Angina Threshold

All 65 patients underwent rest angina threshold assessment. The mean peak supine heart rate was 67±11 bpm; the mean arterial lactate concentration was 1.1±0.4 mmol/L. The median time to onset of angina from the start of balloon inflation was 100 seconds (IQR, 70–189 seconds).

**Figure 2.**
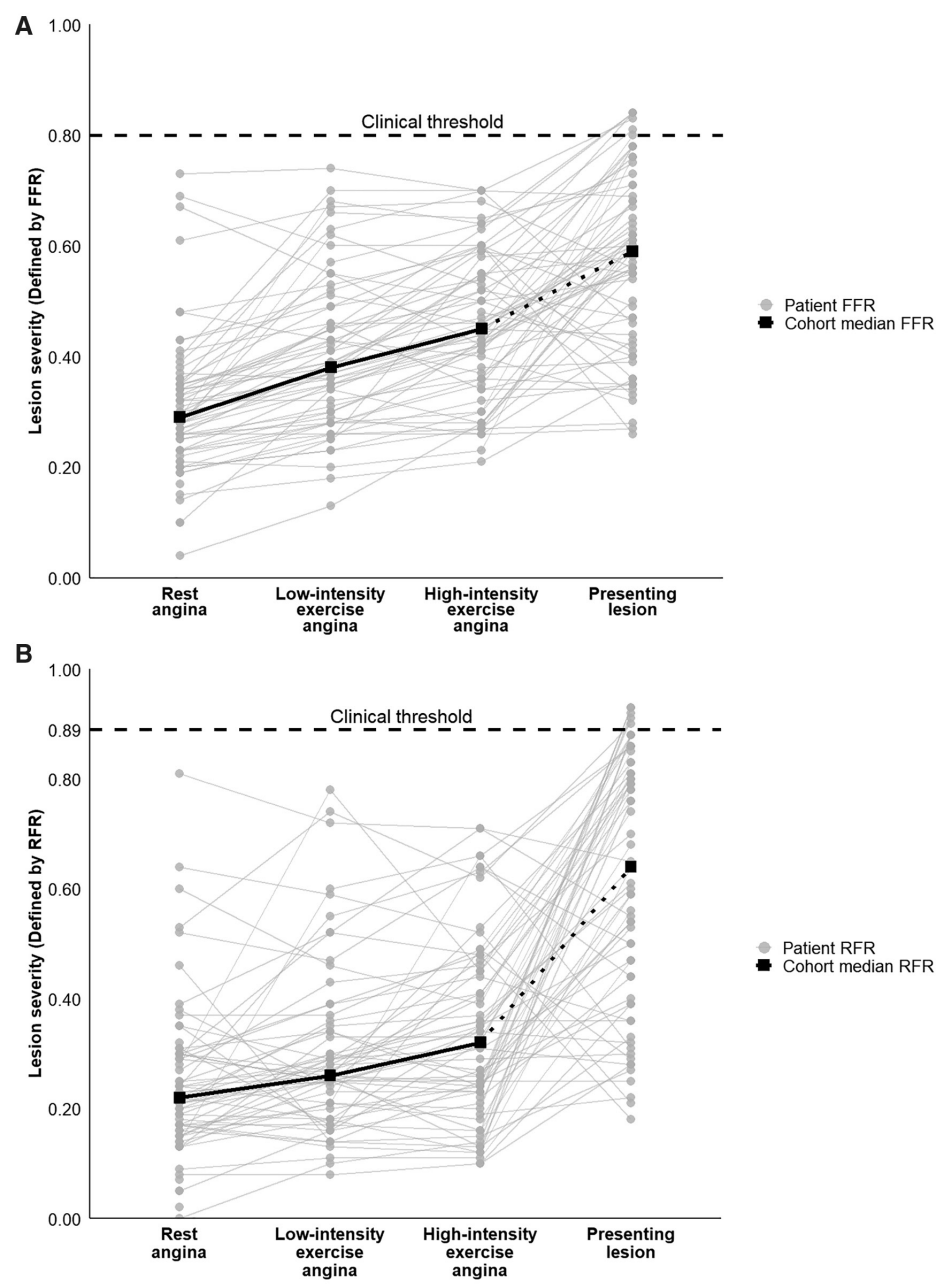
**Physiological thresholds for angina under double-blind, placebo-controlled conditions.** Fractional flow reserve (FFR; **A**) and resting full-cycle ratio (RFR; **B**) are shown at 4 key stages: the angina thresholds at rest, during low-intensity exercise, and during high-intensity exercise and the presenting lesion severity before percutaneous coronary intervention. Each gray dot represents an individual patient, and gray lines show within-patient changes in FFR/ RFR with increasing exercise. Black squares denote cohort medians, and the dotted horizontal line corresponds to the clinical threshold for intervention (FFR ≤0.80, RFR ≤0.89). All median thresholds were significantly lower than the corresponding clinical diagnostic threshold (*P*<0.001).

At rest, the median Pd/Pa ratio at which patients first reported angina was 0.38 (IQR, 0.33–0.44). Corresponding median FFR_angina_ was 0.29 (IQR, 0.23–0.35), significantly lower than the clinical threshold of FFR ≤0.80 (*P*<0.001). The median RFR_angina_ was 0.22 (IQR, 0.16–0.30), also significantly lower than the clinical threshold of RFR ≤0.89 (*P*<0.001). These data are shown in Figure 2.

### Low-Intensity Exercise Angina Threshold

Sixty-one patients underwent low-intensity exercise angina threshold assessment. Steady-state exercise increased cardiac workload, with a mean supine peak heart rate of 90±15 bpm and a rise in mean arterial lactate concentration to 3.0±1.5 mmol/L, consistent with mild anaerobic activity. The median time to angina from the start of the balloon inflation was 88 seconds (IQR, 62–120 seconds).

During low-intensity exercise (cycling at 50 W continuously), patients developed angina at a median Pd/Pa of 0.44 (IQR, 0.37–0.52). Corresponding median FFR_angina_ was 0.38 (IQR, 0.30–0.48), significantly lower than the clinical threshold of FFR ≤0.80 (*P*<0.001). The median RFR_angina_ was 0.26 (IQR, 0.18–0.36), also well below the clinical thresholds of RFR ≤0.89 (*P*<0.001). These data are shown in Figure 2.

### High-Intensity Exercise Angina Threshold

All 65 patients underwent high-intensity exercise angina threshold assessment. At peak exercise, the mean supine peak heart rate was 108±15 bpm, and nearly all patients (n=61, 93.8%) reached ≥85% of their pre-PCI angina-triggering target heart rate, indicating optimal exercise levels. Mean arterial lactate concentration was 4.3±2.0 mmol/L, consistent with significant anaerobic activity. The median time to angina from the start of the balloon inflation was 102 seconds (IQR, 82–164 seconds).

During high-intensity exercise, angina was provoked at a median Pd/Pa of 0.51 (IQR, 0.43–0.60). Corresponding median FFR_angina_ was 0.45 (IQR, 0.36–0.55), significantly lower than the clinical threshold of FFR ≤0.80 (*P*<0.001). The median RFR_angina_ was 0.32 (IQR, 0.23–0.46), and this was also significantly lower than the clinical thresholds of RFR ≤0.89 (*P*<0.001). These data are shown in Figure 2.

### Effect of Cardiac Workload on the Angina Threshold

**Figure 3.**
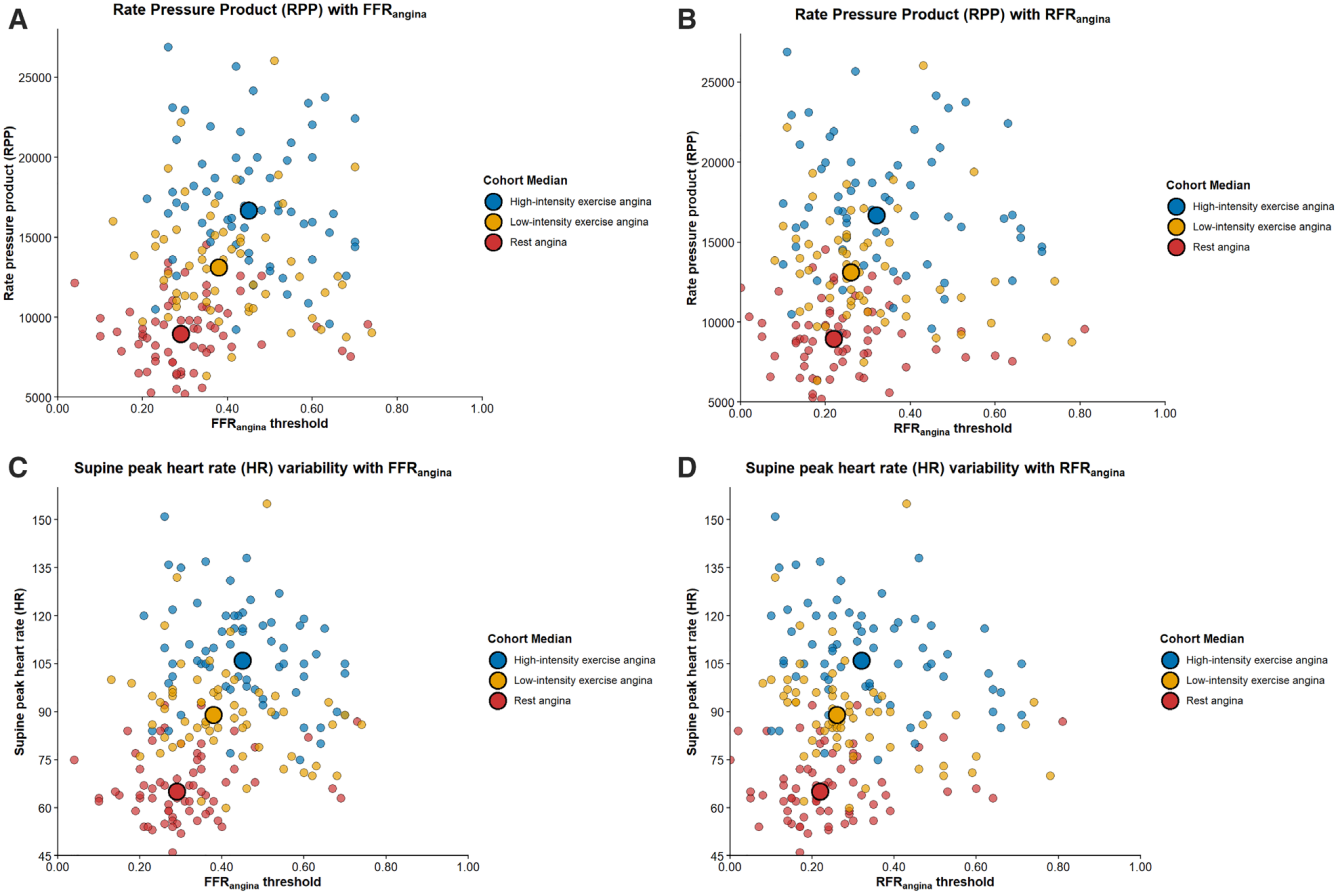
**Relationship between cardiac workload and the angina thresholds. A**, Relationship between rate–pressure product (RPP) and fractional flow reserve at the point of angina (FFR_angina_); (**B**) relationship between RPP and resting full-cycle ratio at the point of angina (RFR_angina_); (**C**) relationship between supine peak heart rate (HR) and FFR_angina_; and (**D**) relationship between supine peak HR and RFR_angina_. Each point represents an individual angina threshold measured at rest (red), during low-intensity exercise (yellow), or during high-intensity exercise (blue); larger circles indicate the median for each stage. With increasing exercise intensity, both FFR_angina_ and RFR_angina_ rose progressively in parallel with cardiac workload. RPP and HR increased significantly across all pairwise comparisons (*P*<0.001).

As cardiac workload increased, reflected by rising rate-pressure product, supine peak heart rate, and systolic blood pressure, angina thresholds increased in parallel (Figure 3). FFR_angina_ values were significantly higher during exercise, when myocardial oxygen demand is greater, compared with rest (*P*<0.001) and increased further from lower to higher workload states (*P*<0.001). Similarly, RFR_angina_ values increased from rest to low-intensity (*P*=0.007) and high-intensity (*P*<0.001) exercise, and between low- and high-intensity exercise states (*P*=0.007).

### ORBITA App Data and Symptom Questionnaires

**Figure 4.**
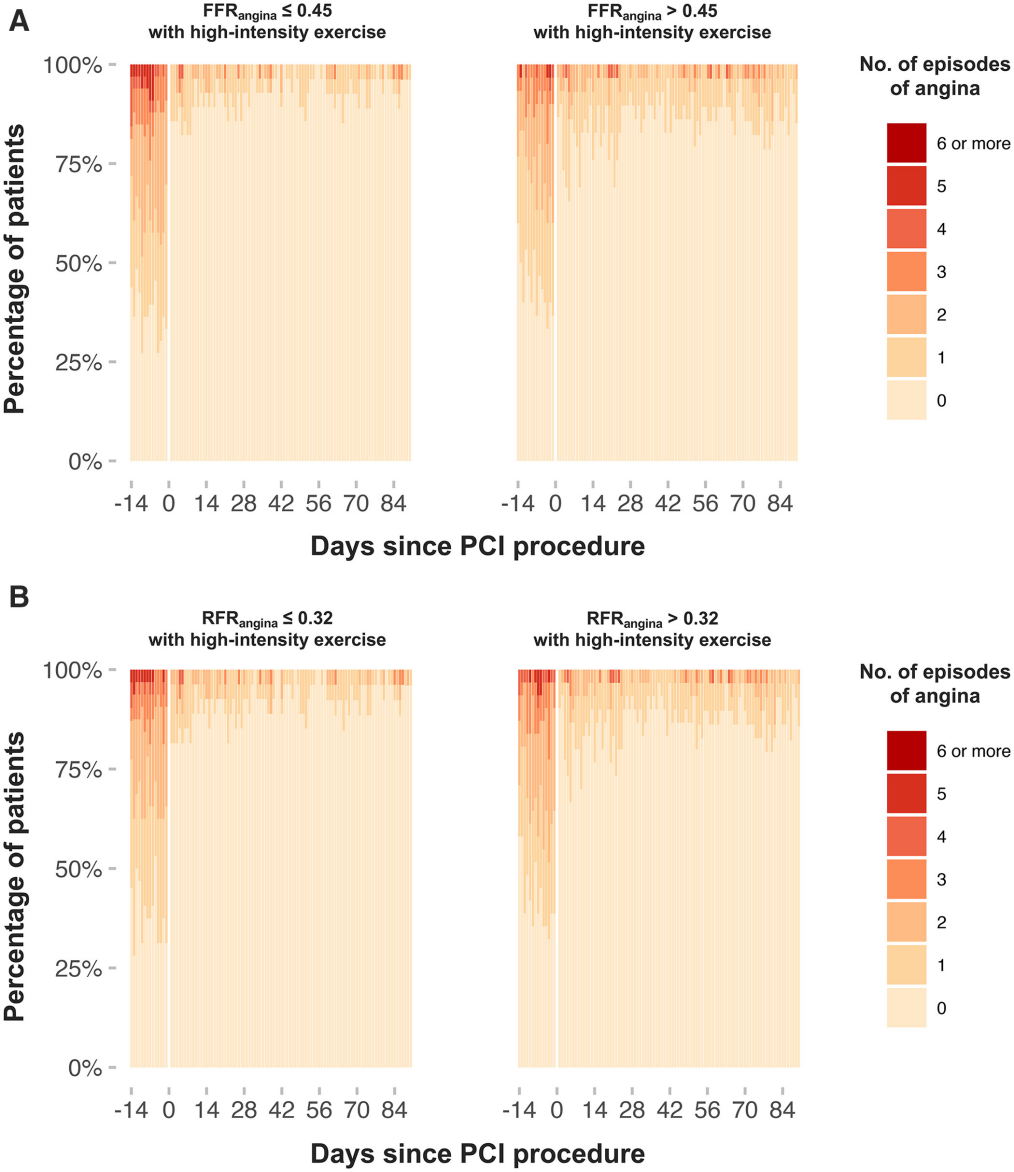
**Effect of PCI on daily angina episodes during high-intensity exercise.** Heat maps show the proportion of patients reporting daily angina episodes before and after percutaneous coronary intervention (PCI), stratified by (**A**) median fractional flow reserve at the point of angina (FFR_angina_; ≤0.45 vs >0.45) and (**B**) median resting full-cycle ratio at the point of angina (RFR_angina_; ≤0.32 vs >0.32) with high-intensity exercise. Each vertical bar represents an individual patient-day, with color intensity indicating the number of angina episodes. Day 0 marks the PCI procedure. During high-intensity exercise, higher FFR_angina_ and RFR_angina_ thresholds predicted markedly less improvement in daily angina frequency compared with lower thresholds (FFR_angina_ odds ratio [OR], 0.49 [95% CrI, 0.33–0.66]; RFR_angina_ OR, 0.37 [95% CrI, 0.18–0.55]; Pr(interaction) >0.999 for both).

Symptom app data were available for 64 patients. Comparison was made between patients in the upper and lower quartiles of FFR_angina_ and RFR_angina_ before and after PCI. Patients with lower FFR_angina_ thresholds reported higher baseline frequency of daily angina episodes compared with those with higher thresholds (Table S8). During high-intensity exercise, there was strong evidence of interaction between both FFR_angina_ and RFR_angina_ and the symptomatic effect of PCI, with higher thresholds predicting markedly less improvement in daily angina frequency compared with lower thresholds (FFR_angina_ odds ratio, 0.49 [95% CrI, 0.33–0.66]; Pr(interaction) >0.999; RFR_angina_ odds ratio, 0.37 [95% CrI, 0.18–0.55]; Pr(interaction) >0.999; Figure 4). Similar findings were observed at low-intensity exercise (Figures S19 and S23). In contrast, at rest, this differential effect was attenuated; both quartile groups experienced significant benefit (Figures S11 and S15). There was no relationship between FFR_angina_ or RFR_angina_ values and Canadian Cardiovascular Society class, Rose angina, Seattle Angina Questionnaire domains, or the pain-sensitivity questionnaire at baseline (Tables S10–S17).

### Angina Threshold Reproducibility

The reproducibility of the angina threshold was assessed under double-blind placebo-controlled conditions across rest and exercise states. Reproducibility was defined as the onset of angina at the same Pd/Pa threshold after placebo-controlled reinflation of the intracoronary balloon. At rest, balloon inflation to the Pd/Pa associated with angina onset reproduced symptoms at the same threshold in 50 of 65 patients (76.9%) compared with placebo inflation. Reproducibility was confirmed during low-intensity exercise in 49 of 61 patients (80.3%) and during high-intensity exercise in 52 of 65 patients (80.0%). These results indicate robust reproducibility of angina thresholds across varying physiological states.

**Figure 5.**
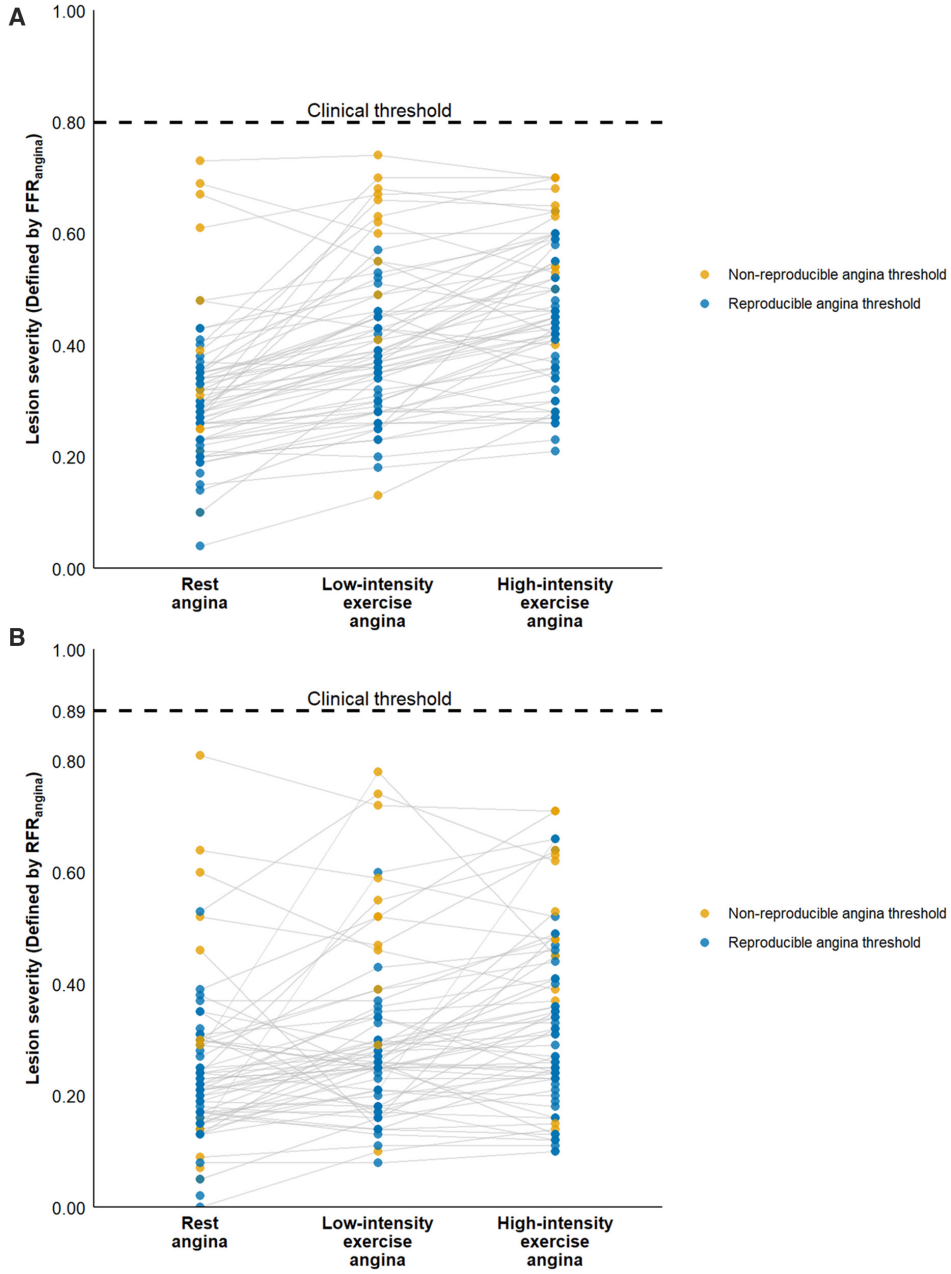
**Symptom reproducibility at the individualized angina thresholds across exercise states.** Fractional flow reserve at the point of angina (FFR_angina_; **A**) and resting full-cycle ratio at the point of angina (RFR_angina_; **B**) are shown for each participant at rest, during low-intensity exercise, and during high-intensity exercise. Colored dots indicate reproducibility of angina at each patient-specific threshold under double-blind, placebo-controlled conditions (blue for reproducible thresholds and yellow for nonreproducible thresholds). Reproducibility was defined as the onset of angina at the same distal to aortic pressure ratio (Pd/Pa) threshold after placebo-controlled reinflation of the intracoronary balloon. Dotted horizontal line corresponds to the clinical threshold for intervention (FFR ≤0.80, RFR ≤0.89). Lower FFR at the point of angina (FFR_angina_) thresholds were associated with greater symptom reproducibility across rest and low- and high-intensity exercise (*P*=0.008, *P*<0.001, *P*<0.001), with similar findings for RFR at the point of angina (RFR_angina_; *P*=0.015, *P*<0.001, *P*=0.002).

Notably, the lower the FFR_angina_ threshold was, the more consistently symptoms were reproduced across rest and low-intensity and high-intensity exercise conditions (*P*=0.008, *P*<0.001, and *P*<0.001, respectively). Equally, lower RFR_angina_ values were also associated with greater symptom reproducibility across rest and low-intensity, high-intensity exercise conditions (*P*=0.015, *P*<0.001, and *P*=0.002, respectively). These findings are illustrated in Figure 5. The degree of cardiac workload (rest, low-intensity and high-intensity exercise) did not influence the reproducibility of the angina threshold (*P*=0.872).

### Angina Symptom Intensity and Similarity

At each angina threshold, patients characterized their experimental symptoms and reported the severity (0–10) and similarity (0–10) compared with their typical day-to-day symptoms. The predominant symptom characteristic was chest tightness in 62 patients (95.5%). Other characteristics were uncommon and included sharp chest pain (n=1, 1.5%), shoulder tip discomfort (n=1, 1.5%), and retrosternal burning (n=1, 1.5%). Symptom severity and similarity scores were consistent across the different angina thresholds. At rest, the median symptom severity score was 3 (IQR, 2–3), with a median angina similarity score of 10 (IQR, 10–10). During low-intensity exercise, the median symptom severity score was 2 (IQR, 1–4), and the similarity score remained at 10 (IQR, 10–10). Similarly, during high-intensity exercise, the median severity score was 2 (IQR, 2–4), and the similarity score was also 10 (IQR, 10–10). There was no significant difference in reported symptom severity across all 3 conditions (*P*=0.817).

### Vessel-Specific Angina Thresholds

There was no statistically significant difference in the FFR_angina_ or RFR_angina_ thresholds between LAD and non-LAD lesions across rest, low-intensity and high-intensity exercise conditions (FFR_angina_: *P*=0.757, *P*=0.416, *P*=0.757; RFR_angina_: *P*=0.071, *P*=0.616, *P*=0.616, respectively). In patients with LAD disease, thresholds did not differ between proximal and nonproximal lesions for either FFR_angina_ or RFR_angina_ (Tables S3–S5).

### Procedural Safety

Safety was prioritized throughout the invasive protocol, with continuous hemodynamic and electrocardiographic monitoring and predefined stopping criteria. Systematic preprocedural and postprocedural ECGs, troponins, and clinical assessments were performed. No serious adverse events occurred during balloon inflations or supine bicycle exercise. Minor transient symptoms such as brief hypotension or mild arrhythmias resolved spontaneously without intervention. No vessel dissection, stent thrombosis, sustained arrhythmias, or periprocedural myocardial infarctions were observed in any patients during the study.

## Discussion

This is the first double-blind, randomized, placebo-controlled study to define individualized physiological thresholds for angina, FFR_angina_ and RFR_angina_, in patients with stable single-vessel CAD. The symptom-linked thresholds were highly reproducible within patients and varied substantially between individuals and across levels of cardiac workload. Importantly, patients with lower angina thresholds were more symptomatic at baseline and achieved the greatest angina relief with PCI, whereas those with higher thresholds had fewer symptoms, less reproducible angina, and derived limited benefit. These findings highlight marked interindividual variability in the physiological threshold for angina onset and suggest that fixed, population-derived clinical cut points for ischemia should be interpreted with caution when applied to individual patients for the purpose of predicting angina relief.

The historical rationale for PCI in stable CAD is grounded in a conventional pathophysiological model: Ischemia provokes angina, and relieving ischemia relieves angina. In this framework, oxygen supply–demand imbalance triggers anaerobic metabolism, accumulation of metabolites (eg, adenosine, lactate), and activation of cardiac nociceptors, often accompanied by wall motion abnormalities, diastolic dysfunction, or electrocardiographic changes.^45,46^ However, the mechanisms underlying angina perception remain incompletely understood and likely involve a continuum of neural and metabolic pathways. Proposed mechanisms include activation of adenosine A1-A2 receptor complexes, myocardial stretch receptors, and local ischemic metabolites, each contributing variably to the complex perception of angina.^47^ For decades, the clinical translation has been deeply embedded: identify ischemia with a fixed physiological threshold, treat the stenosis, normalize flow, and thereby resolve angina.

This mechanistic view shaped the development and adoption of FFR, introduced in 1993 to quantify coronary flow limitation as the Pd/Pa during maximal hyperemia.^48,49^ FFR is intrinsically flow-dependent and reflects a composite of both epicardial resistance and microvascular vasodilatory capacity, rather than an isolated measure of lesion severity alone.^48^ Early validation studies proposed ischemic thresholds between 0.66 and 0.74, but variability led to the more conservative adoption of 0.75, as demonstrated in DEFER (Deferral Versus Performance of PTCA in Patients Without Documented Ischaemia), the first randomized trial to support the safety of deferring PCI at or above this threshold.^11,27,50,51^ The FAME trials (Fractional Flow Reserve Versus Angiography for Multivessel Evaluation) extended this principle, showing that PCI could be deferred safely in lesions with FFR >0.80 and highlighted the poor correlation between angiographic and physiological severity.^12^ Subsequently, FFR ≤0.80 became synonymous with "ischemia," often justifying PCI even in the absence of symptoms, stress-induced ST depression, or left ventricular dysfunction, an evolution beyond the original trial rationale.^7,8^ Nonhyperemic indices such as instantaneous wave-free ratio and RFR have since emerged as adenosine-free alternatives with thresholds of ≤0.89.^52,53^ As a result, guidelines now assign Class I (Level A) recommendations to physiology-guided PCI when prior evidence of ischemia is lacking.^7,8^ However, the landscape of stable CAD has changed since the advent of these trials. Revascularization does not appear to have a significant prognostic benefit, and its primary role has shifted toward symptom relief.^1,19,54^ However, despite this shift in clinical emphasis, the relationship between these physiological thresholds and angina has never been defined in a placebo-controlled setting.

This knowledge gap is clinically significant. ORBITA-2 (A Placebo-Controlled Trial of Percutaneous Coronary Intervention for the Relief of Stable Angina) and ISCHEMIA (International Study of Comparative Health Effectiveness With Medical and Invasive Approaches) highlighted a persistent disconnect between measured physiology and patient-reported symptoms: 59% of patients in the blinded ORBITA-2 trial remained symptomatic despite normalized post-PCI physiology,^4^ whereas one-third of patients in the unblinded ISCHEMIA trial were asymptomatic despite moderate to severe ischemia.^1^ Similar variability is seen with positron emission tomography; many patients with markedly reduced perfusion are asymptomatic, and others with only mild flow limitation have severe angina relieved by PCI.^18^ Collectively, these observations reveal a fundamental problem: Although ischemia and angina often coexist, the relationship between them is not reliably synchronous.

The key limitation is the assumption that a universal threshold can identify symptomatic angina in a clinically heterogeneous population with diverse physiological and perceptual responses.^25,26^ Although fixed cut points facilitate guideline development, trial design, and reimbursement, they imply a universal tipping point for angina onset, overlooking biological variability and the dynamic influence of cardiac workload. Two patients with identical FFR values may have profoundly different symptom profiles and responses to PCI,^5^ particularly if one has a sedentary lifestyle and the other is physically very active. Reducing a complex physiological continuum to a single dichotomous threshold for all patients risks misclassification and misguided intervention. Precision PCI begins by recognizing that the onset of angina is not governed by a universal physiological threshold developed for clinical outcomes.

In this study, FFR_angina_ and RFR_angina_ varied significantly across patients and were consistently lower than clinical thresholds used to guide PCI (Figure 2). These findings support the concept of individualized symptom-linked patient thresholds shaped by myocardial oxygen demand, autonomic tone, microvascular health, and central pain processing.^20,23,24,55^ They also highlight inherent limitations of interpreting FFR as an isolated measure of stenosis severity. For example, an FFR of 0.70 may reflect either high absolute flow across an angiographically mild lesion or true flow limitation across a severe stenosis, depending on the amount of perfused myocardium subtended.^56^ This further justifies exploring individualized angina thresholds in a population enriched for severe epicardial disease. The results align with prior work demonstrating that angina arises from an interplay of anatomical, physiological, and perceptual factors rather than anatomic severity alone.^21,30,57^

The lower thresholds identified in this study may reflect the elimination of cognitive and anticipatory bias under double-blind placebo-controlled conditions. In daily practice, angina perception is heavily influenced by expectation, interoception, and prior experience.^23,57^ Patients conditioned by past angina episodes may report symptoms even in the absence of significant ischemia.^58^ Neural sensitization from recurrent ischemia (ischemic memory) may lower pain thresholds, adding further complexity to real-world symptom expression.^23,57^ By removing anticipatory bias, this protocol more precisely isolated the true physiological stimulus required to provoke angina.

Another key finding was that angina thresholds increased systematically with increasing cardiac workload, confirming the dynamic nature of symptom onset. As heart rate and myocardial oxygen demand rose, the physiological severity of the stenosis required to provoke angina decreased proportionally (Figure 3). This workload-dependent relationship highlights the limitations of relying solely on nonexercise measurements in clinical practice. Although hyperemic indices partially approximate exercise physiology, these data show that real-world exertion imposes distinct hemodynamic demands influencing symptom thresholds.^31,32,59^ Although exercise in this study was performed supine in a catheter laboratory setting, where absolute workload and heart rate are typically lower than in upright exercise because of altered loading conditions, the physiological stress achieved was still optimal. This was evidenced by the progressive rise in lactate and the significant stepwise increase in rate-pressure product, peak heart rate, and systolic blood pressure across all stages (Figures S36–S39). The peak rate-pressure product achieved was comparable to, and in some cases higher than, values reported in prior invasive supine catheter laboratory studies.^30,60^ This variability with cardiac workload aligns with findings from exercise physiology studies demonstrating that symptom onset and ischemia thresholds vary with rate-pressure product.^30,61^ The consistency of thresholds across repeated testing supports their physiological validity (Figure 5).

**Figure 6.**
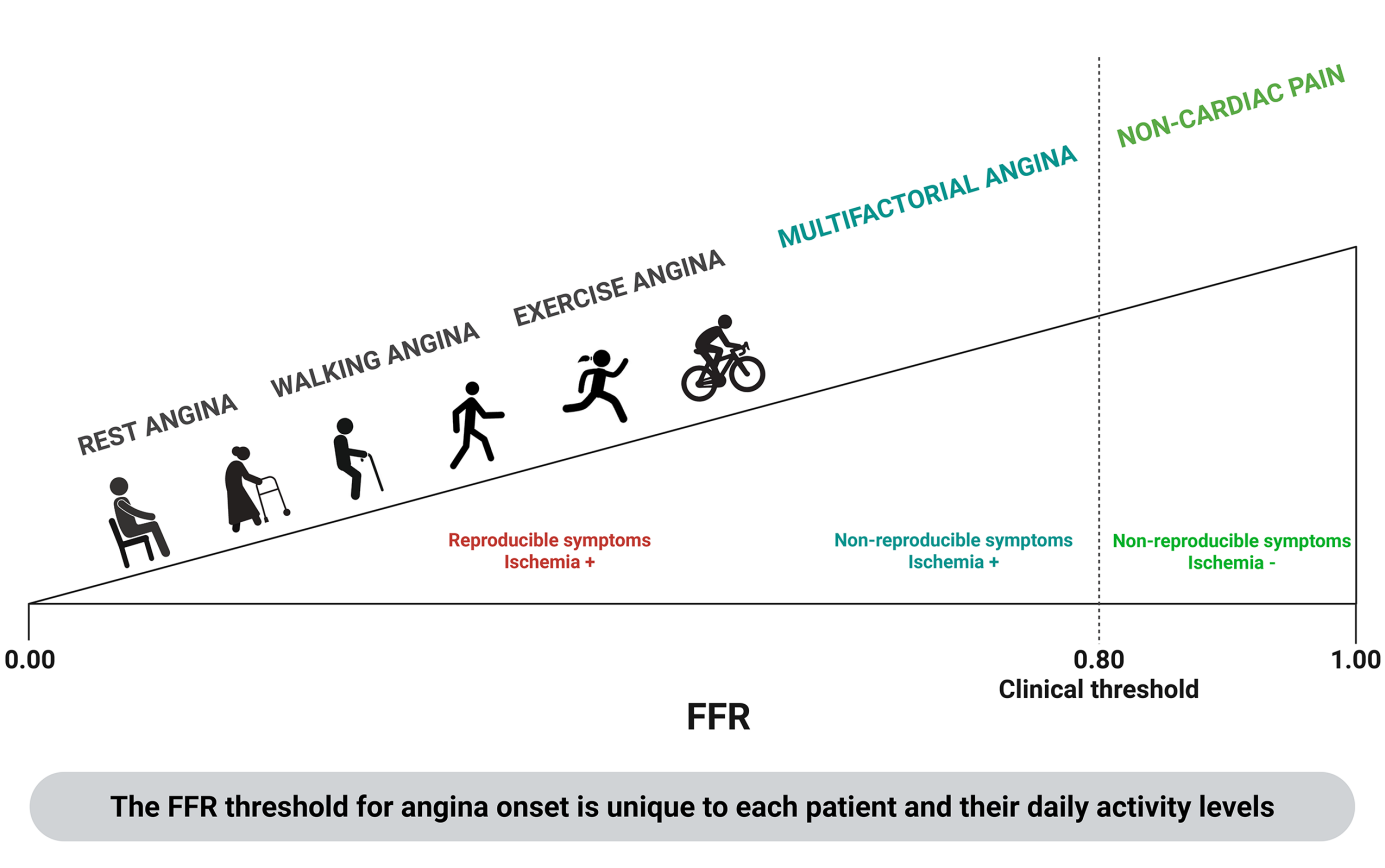
**Conceptual model of patient-specific angina thresholds across the spectrum of cardiac workload.** This schematic illustrates how angina onset varies along a physiological continuum, from rest to exertion, mapped against fractional flow reserve (FFR) values. Clinical cut points (eg, FFR ≤0.80) assume a universal threshold and oversimplify a highly individualized relationship. Two patients with identical FFRs may have very different angina profiles depending on activity level and physiological reserve: A sedentary patient may develop reproducible angina only at low FFR values, whereas an active patient may become symptomatic earlier at higher FFR values. In this study angina thresholds during high-intensity exercise were substantially lower than clinical threshold used to guide revascularization. Moreover, some patients with ischemia demonstrated variable or nonreproducible symptoms at higher thresholds, likely reflecting multifactorial rather than purely epicardial mechanisms. This model highlights the limitations of binary diagnostic thresholds and supports individualized, symptom-linked approach to assessment.

The ORBITA app results reinforce these observations by linking physiological thresholds to patient-reported outcomes (Figure 4). Patients with lower angina thresholds were more symptomatic at baseline and experienced the greatest improvement in daily angina frequency after PCI, whereas those with higher angina thresholds reported fewer baseline symptoms and demonstrated only modest improvement. This gradient of benefit suggests that angina thresholds identify patients whose symptoms are closely coupled to their epicardial stenosis and therefore have the most to gain from revascularization. Conversely, patients with higher FFR_angina_ and RFR_angina_ thresholds may have symptoms that are less reproducible and not driven primarily by epicardial disease, whether due to lower daily cardiac workload, greater microvascular reserve, or nonischemic contributors to chest pain (Figure 6). Notably, conventional tools such as Canadian Cardiovascular Society class, the Rose angina questionnaire, Seattle Angina Questionnaire scores, and even the pain-sensitivity questionnaire did not correlate with physiology, highlighting that angina thresholds capture a distinct, patient-specific biological phenotype that more accurately predicts who benefits from PCI. Persistent post-PCI symptoms recorded on the symptom app, particularly among patients with higher FFR_angina_ and RFR_angina_ values, likely reflect additional mechanisms such as microvascular dysfunction, vasospasm, or altered pain processing that may coexist with epicardial disease rather than representing procedural failure. Recognizing these overlapping contributors is essential for comprehensive angina management and emphasizes that patients who respond to optimized antianginal therapy are generally those most likely to benefit from PCI, whereas those with multifactorial angina refractory to medical treatment may derive less symptomatic improvement.^4^

Because our assessments were performed after PCI rather than in native lesions, the potential influence of ischemic preconditioning warrants consideration. The post-PCI model was chosen for ethical and patient safety reasons, allowing repeated, titratable ischemic provocations that would not have been permissible in untreated vessels. Although preconditioning could theoretically affect symptom physiology, several features of the protocol mitigated this concern: Measurements were obtained under double-blind, placebo-controlled conditions; the order of balloon inflations was randomized; and there was a substantial interval between repeated provocations. Importantly, the angina-associated Pd/Pa was highly reproducible at the exact same threshold despite repeated inflations across different time points. Moreover, prior work has shown that sequential inflations do not affect acute collateral recruitment or pain intensity within the same individual, providing no evidence of acute ischemic preconditioning.^62^ These features make a substantial contribution from ischemic preconditioning unlikely.

Taken together, these findings illustrate that although clinical cut points reliably identify flow-limiting disease at a population level, the physiological threshold at which angina occurs is highly individualized and does not align with a single universal value. An FFR ≤0.80 or nonhyperemic pressure ratio ≤0.89 should therefore not be viewed as an absolute threshold for intervention but rather as a value within a broader physiological continuum that must be contextualized with angina burden, activity level, microvascular function, pain perception, and psychological factors. Optimal care should integrate epicardial physiology with coronary function testing and, when appropriate, evaluation of pain perception and central modulation. By defining FFR_angina_ and RFR_angina_ thresholds under blinded, placebo-controlled invasive conditions, this study emphasizes the need to move beyond the one-size-fits-all paradigm of ischemia-driven PCI toward a personalized, symptom-guided approach to improve symptomatic outcomes with PCI.

ORBITA-FIRE is a mechanistic study demonstrating that angina thresholds can be precisely defined and measured in a placebo-controlled setting. These data complement noninvasive perfusion imaging by linking invasive pressure–derived physiology directly to symptom experience, thereby informing more precise and individualized revascularization decisions.^18,63^ Translating these physiological thresholds into clinical practice will require further studies to identify key modifiers of symptom perception and reproducibility, including autonomic tone, daily cardiac workload, microvascular health, collateral circulation, and psychosocial factors. Ultimately, this proof-of-concept study suggests a potential direction toward a more individualized, symptom-focused revascularization strategy: treating the right lesion, in the right patient, for the right reason.

### Limitations

Several important limitations should be acknowledged. The study cohort was relatively small and selective. It was designed to explore variability in patient-specific physiology rather than to redefine clinical thresholds. All patients had single-vessel disease with angiographically severe stenoses, objective ischemia, and were sufficiently fit to complete exercise testing. Although necessary for safety and feasibility, these criteria may limit generalizability to broader populations. Furthermore, a significant proportion of patients had LAD disease, limiting insights into vessel-specific differences in angina thresholds.

The research protocol was performed after PCI. Although this design was essential to permit safe in-stent balloon provocations under placebo-controlled conditions, it raises the possibility that postprocedural changes such alteration is microvascular integrity and flow dynamics could have influenced angina thresholds.

Balloon inflation was used to simulate flow limitation rather than inducing true native stenosis physiology. Although this approach allowed precise and reproducible control of coronary pressure, it may not fully replicate the complex flow dynamics or plaque-related characteristics of native lesions. In addition, given the extensive invasive protocol, pullback pressure gradients, lesion length, and plaque morphology were not systematically evaluated.

Exercise protocols offer greater signal-to-noise ratio but were conducted in a controlled supine setting, unlike real-world upright exertion in which emotional and neurohormonal factors may affect angina thresholds. Nonetheless, clinical invasive physiology can be measured only in a supine state in cardiac catheterization laboratory.

Although patients were randomized to the order of rest and exercise, those who exercised first may have experienced warm-up angina, a phenomenon in which prior ischemia delays symptom onset on repeat exposure as a result of improved perfusion or preconditioning.^60^

Follow-up symptom data were unblinded and uncontrolled in that PCI was required by the study design. While unblinded evaluation limits accurate quantification of the true physical effect of treatment, patients in this study were blinded to the physiological thresholds measured in the catheterization laboratory, and the symptom-tracking app was completed independently without researcher interaction. This ensured that the relationship between physiological thresholds and angina response could still be quantified.

Last, although the placebo-controlled design reduced bias, symptom perception remains inherently subjective and may be influenced by individual pain perceptions, expectations, and psychological factors.^22,23^

### Conclusions

This is the first double-blind, randomized, placebo-controlled study to define patient-specific physiological thresholds for angina, FFR_angina_ and RFR_angina_, in stable single-vessel CAD under controlled invasive catheter laboratory conditions. These thresholds were highly reproducible, varied substantially between individuals, and increased with cardiac workload. Symptom data from the ORBITA app showed that lower angina thresholds were associated with greater baseline angina burden and more pronounced angina relief after PCI. By introducing the concept of individualized physiological thresholds for symptoms, this mechanistic study provides early evidence supporting a more personalized, symptom-driven approach to revascularization. Future studies are needed to determine which physiological cut points are appropriate for specific patient populations, and whether integrating symptom-linked thresholds along with established diagnostic tools improves patient selection and angina outcomes beyond current practice.

## Article Information

### Disclosures

Dr Ahmed-Jushuf has received speaker fees from Philips. Dr Foley has received speaker fees from Menarini and has received consulting and speaker fees from Shockwave Medical, Inc and Philips. Dr Chotai has received sponsorship from Servier Pharmaceuticals and speaker fees from Philips. Dr Rajkumar has received speaker fees from Menarini and Novartis, has received consulting fees from Philips, and has received shares from Mycardium AI. Dr Simader has received a sponsorship from Servier Pharmaceuticals. Dr Keeble has served on advisory boards for Abbott Vascular, Abiomed, and Philips; has received institutional research funding from Terumo and Abbott Vascular; and has received speaker fees from AstraZeneca, Nipro, Cathworks, and Shockwave Medical. Dr Davies has received grants from Medtronic and Abbott; has received sponsorship from Vascular Perspectives, Boston Scientific, Medtronic, and Abbott; and has received speaker fees from AstraZeneca, Pfizer, Bristol Myers Squibb, and Novartis. Dr Din has received speaker fees from Philips and Vascular Perspective. Dr O’Kane has received speaker fees from Abbott Vascular, Biosensors, Boston Scientific, Heartflow, Medtronic, Philips, Shockwave, and Terumo. Dr Nijjer has received speaker fees from Philips Volcano, Pfizer, Bayer, AstraZeneca, Boehringer Ingelheim, and Amarin. Dr Hinton has received speaker fees from Boston Scientific Corp, Medtronic, Shockwave Medical, Inc, and Cordis, as well as research funding from Abbott. Prof Spratt has received speaker fees from Boston Scientific Corp and Shockwave Medical, Inc. Dr Dungu has received shares in Mycardium AI. Prof Harrell has received consulting fees from Annexon and Regeneron. Dr Howard has received shares in Mycardium AI and has received a grant from the British Heart Foundation. Dr Shun-Shin has received shares in Mycardium AI. Prof Al-Lamee has served on advisory boards for Janssen Pharmaceuticals, Abbott, and Philips and has received speaker fees from Abbott, Philips, Medtronic, Servier, Omniprex, and Menarini. The other authors reported no conflicts.

### Supplementary Material

Supplemental Appendix

Tables S1–S21

Figures S1–S38

## Supplementary Material


