# Supplementary material for: Determining the Physiological Threshold for Angina (ORBITA-FIRE): A Double-Blind, Randomized, Placebo-Controlled Study
Source: Circulation. 2026 May 8;153(23):1795–812. doi: 10.1161/CIRCULATIONAHA.125.078738 (PMC13236056; doi:10.1161/CIRCULATIONAHA.125.078738)
Supplement: Supplementary file 1 [file cir-153-1795-s001.pdf]

ORBITA-FIRE

Supplemental Material

Table of Contents

Trial Conduct **4**

List of trial sites **4**

ORBITA-FIRE Investigators **4**

Statistical Working Group **5**

Writing Committee **5**

Supplemental Methods **6**

Inclusion and exclusion criteria 6
Supplemental Figure S1: Consort diagram 7
Sample size and power calculations 7
Bayesian analysis for angina frequency 8
Odds ratio for angina frequency 9

Patient enrolment and pre-assessment **10**

Smartphone ORBITA-App 10
Supplemental Figure S2: Screenshots from ORBITA-App 11
Smart watch 12

Questionnaires **12**

Seattle Angina Questionnaire 12
Rose angina questionnaire 12
Pain Sensitivity Questionnaire 12

Medication prescribing protocol **13**

Supplemental Table S1: Antianginal medication quantification 14

Cardiac Catheterization Protocol **15**

Physiological measurements and indices **15**

Supplemental Table S2: Hemodynamic Indices 15
Protocol for assessing RFR 15
Protocol for assessing CFR/IMR 16
Protocol for assessing FFR 16
Collateral Flow Index (CFI) measurement 16
Quantitative coronary angiography (QCA) Analysis 17

Exercise Protocol **17**

Supplemental Figure S3: Balloon inflations during supine exercise 17
Supplemental Figure S4: Physiology screenshots with exercise 18

Supplementary results **20**

Supplemental Table S3: Vessels assessed and vessel location 20
Supplemental Table S4: Left anterior descending (LAD) vs non-LAD 20
Supplemental Table S5: Proximal vs non-proximal LAD 21
Supplemental Table S6: Pre-PCI peak heart rates (per patient) 21
Supplemental Table S7: Lactates across all groups (per patient) 21

Logistic regression modeling of the patient-individualized angina thresholds **22**

Supplemental figure S5: FFR_{angina} threshold at rest 22
Supplemental figure S6: RFR_{angina} threshold at rest 23
Supplemental figure S7: FFR_{angina} threshold with low-intensity exercise 24
Supplemental figure S8: RFR_{angina} threshold with low-intensity exercise 25
Supplemental figure S9: FFR_{angina} threshold with high-intensity exercise 26
Supplemental figure S10: RFR_{angina} threshold with high-intensity exercise 27

Effect of PCI with the primary endpoints **28**

Angina symptom frequency **28**

Supplemental Table S8: Comparison of angina frequency burden at baseline............................28
Supplemental Table S9: Ability of angina thresholds to predict benefit with PCI 29
Supplemental Figure S11: FFR_{angina} (at rest) and angina symptom score 30
Supplemental Figure S15: RFR_{angina} (at rest) and angina symptom score 33
Supplemental Figure S19: FFR_{angina} (at low-intensity exercise) and angina symptom score 35
Supplemental Figure S23: RFR_{angina} (at low-intensity exercise) and angina symptom score......38
Supplemental Figure S27: FFR_{angina} (at high-intensity exercise) and angina symptom score.....41
Supplemental Figure S32: RFR_{angina} (at high-intensity exercise) and angina symptom score.....44

Baseline symptom questionnaires ***47***

Canadian Cardiovascular Society (CCS) Class **47**

Supplemental Table S10: CCS class and angina thresholds 47

Rose angina questionnaire **47**

Supplemental Table S11: Rose Angina status and angina thresholds..47

Seattle angina questionnaire (SAQ) **48**

Supplemental Table S12: SAQ Physical limitation and angina thresholds 48
Supplemental Table S13: SAQ Angina Frequency and angina thresholds 48
Supplemental Table S14: SAQ Quality of life and angina thresholds 48
Supplemental Table S15: SAQ stability and angina thresholds...49
Supplemental Table S16: SAQ treatment satisfaction and angina thresholds 49

Pain-sensitivity Questionnaire (PSQ) **49**

Supplemental Table S17: PSQ and angina thresholds 49

Exercise data analysis ***50***

Supplemental Figure S36: Rate–pressure product (RPP) across all exercise stages 50
Supplemental Figure S37: Systolic blood pressure (SBP) and the angina thresholds................50

Antianginal medication record ***52***

Supplemental Table S18: Baseline antianginal medication use 52
Supplemental Table S19: Type and mean daily dose of antianginal medications used 52
Supplemental Table S20: Antianginal medication usage during the study.................................52

Completeness of data collection ***53***

Supplemental Table S21: Data completeness 53

Supplementary Figure S38: Consort checklist ***54***

Trial Conduct

List of trial sites

Imperial College London Healthcare NHS Trust

Mid and South Essex NHS Foundation Trust

University Hospitals of Dorset NHS Foundation Trust

Royal Free London NHS Foundation Trust

Portsmouth Hospitals University NHS Trust

St George's University Hospitals NHS Foundation Trust

ORBITA-FIRE Investigators

Imperial College London Healthcare NHS Trust
Fiyyaz Ahmed-Jushuf, Michael Foley, Shayna Chotai, Christopher Rajkumar, Danqi Wang, Florentina Simader, Krzysztof Macierzanka, Kayla Chiew, Sannidhya Misra, Sukhjinder Nijjer, Henry Seligman, James Howard, Darrel Francis, Matthew Shun-Shin, Rasha Al-Lamee

Mid and South Essex NHS Foundation Trust
Thomas Keeble, John Davies, Gerald Clesham, Klio Konstantinou, Shah Mohd Nazri Jason Dungu

University Hospitals of Dorset NHS Foundation Trust
Peter O'Kane, Jehangir Din, Jonathan Hinton

Royal Free London NHS Foundation Trust
Tushar Kotecha, Daniel Knight

Portsmouth Hospitals University NHS Trust
Peter Haworth

St George's University Hospitals NHS Foundation Trust
James Spratt, Rupert Williams

Statistical Working Group

Dr Fiyyaz Ahmed-Jushuf
Clinical Research Fellow; Imperial College London

Dr Matthew Shun-Shin
Clinical Senior Lecturer and Consultant Cardiologist; Imperial College London

Professor Frank E Harrell, Jr.
Professor of Biostatistics; Vanderbilt University School of Medicine

Dr Rasha Al-Lamee
Reader and BHF Intermediate Research Fellow; National Heart and Lung Institute, Imperial College London

Writing Committee

Dr Fiyyaz Ahmed-Jushuf
Clinical Research Fellow; Imperial College London

Dr Rasha Al-Lamee
Reader and BHF Intermediate Research Fellow; National Heart and Lung Institute, Imperial College London

Dr Matthew Shun-Shin
Clinical Senior Lecturer and Consultant Cardiologist; Imperial College London

Supplemental Methods

Inclusion and exclusion criteria

Inclusion criteria

1. Angina or angina-equivalent symptoms
2. Anatomical evidence of a significant single vessel coronary stenosis on computerized tomography coronary angiography (CTCA) or diagnostic coronary angiography indicating ≥ 70% stenosis
3. Evidence of ischemia, on any of the following tests:
    a. Dobutamine stress echocardiography (DSE)
    b. Stress perfusion cardiac magnetic resonance imaging (MRI)
    c. Nuclear medicine myocardial perfusion scan
    d. Invasive pressure wire assessment suggestive of ischemia, as judged by the interventional cardiologist, at the time of clinical or research coronary angiography
4. Referral for percutaneous coronary intervention for treatment of stable angina

Exclusion criteria

1. Age younger than 18
2. Age older than 85
3. Recent acute coronary syndrome
4. Multivessel coronary artery disease
5. Previous coronary artery bypass graft surgery
6. Significant left main stem coronary disease
7. Chronic total occlusion in the target vessel
8. Contraindication to percutaneous coronary intervention or drug-eluting stent implantation
9. Contraindication to antiplatelet therapy
10. Contraindication to adenosine infusion
11. Severe valvular disease
12. Severe LV systolic impairment
13. Severe respiratory disease
14. Life expectancy less than 2 years, pregnancy, unable to consent

Supplemental Figure S1: Consort diagram

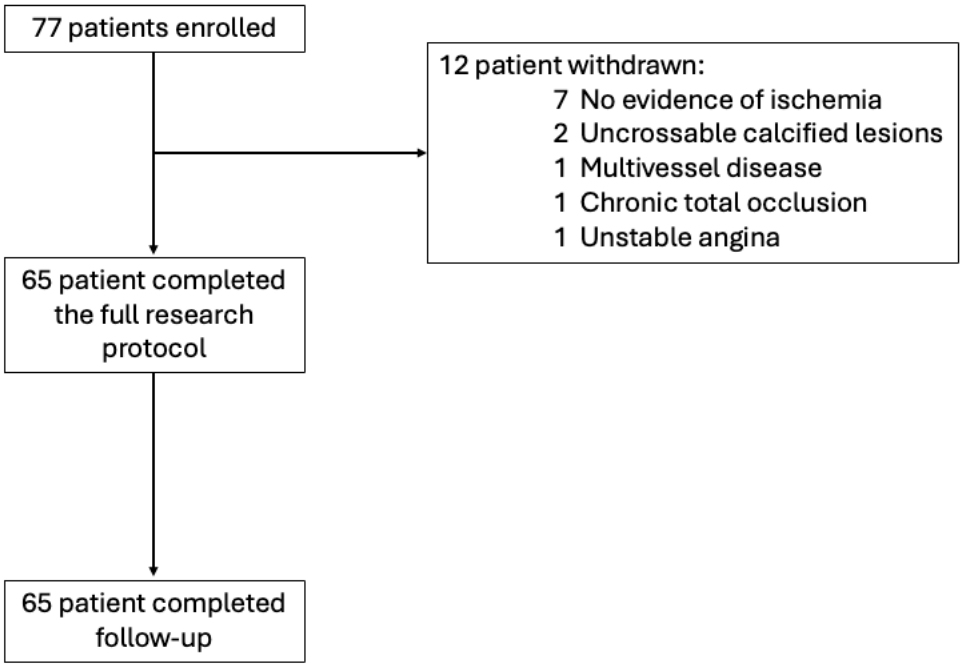

Each withdrawn participant was allocated a singular reason for exclusion.

Sample size and power calculations

It was hypothesized that the resting angina threshold will be lower than the exercise angina threshold. A conservative standard deviation difference of 0.06 for reproducibility of FFR was taken.[27]

To detect a change of 0.03 units with 90% power (Z_{β}=1.282) at the 5% two-tailed significance level (Z_{α}=1.960), the required sample size was:

= $(Z_{\alpha}+Z_{\beta})^2$ x (standard deviation of difference/ change)^2
= 10.51 x $(0.06/0.03)^2$ = **42 patients**

Allowing for an estimated 10% false-positive referral rate from CT coronary angiography, 10% dropout due to absence of symptoms, and a 10% dropout due to inability to comply with the exercise protocol, a total of 58 patients were planned for recruitment (42/0.9/0.9/0.9 **= 58**).

Bayesian analysis for angina frequency

Bayesian analysis for angina frequency

The number of daily anginal episodes was recorded throughout the trial using a mobile phone application (ORBITA-App), completed by participants for 14 days before the research PCI and for up to 3 months (12 weeks) afterwards.

The symptom endpoint, frequency of daily angina episodes (an ordinal outcome), was analysed using a longitudinal, first-order Markov ordinal model within a Bayesian framework.[4,43]

Model structure: The number of daily angina episodes (ordinal outcome) was analysed using a longitudinal, first-order Markov Bayesian proportional odds model to account for within-patient correlation across serial measurements. FFR_{angina} and RFR_{angina} were entered in separate models. Analyses were performed separately for rest, low-intensity exercise, and high-intensity exercise conditions.

The dependent variable was the “number of episodes of angina,” the previous day’s numbers of episodes angina (i.e. a first order Markov Model). Independent variables included the pre-procedural mean of the same component with an interaction term for FFR_{angina} and $RFR_{angina,}$ and clustering of scores within patients was accounted for. This method has been described previously.[4,43]

Restricted cubic splines: Three knots were selected to allow flexible modelling of potential nonlinearity in the previous day’s number of angina, and the previous mean number of angina episodes at baseline .

Priors: Intercepts were specified using a Dirichlet-induced prior on cumulative cell probabilities, when all covariates were set to their means. This enforced a strict ordering of the intercepts since they were defined by logits of cell probabilities accumulated over increasing values of the response.

Prior covariates: A virtually flat prior was used, i.e., a distribution with mean 0 and standard deviation of 100 on a normalised covariate scale.

Convergence diagnostics: Model convergence was assessed using trace plots, Gelman–Rubin R-hat statistics, and effective sample size estimates.

Posterior interpretation: Posterior probabilities >0.975 were considered strong evidence of an association.

Odds ratio for angina frequency

The Bayesian proportional odds model estimated the association between physiological angina thresholds and ordinal categories of daily angina frequency recorded in the ORBITA-App. Odds ratios represent the change in the odds of being in a higher angina-frequency category per unit decrease in physiological threshold.

An odds ratio <1 indicates lower odds of being in a higher angina-frequency category after PCI per unit decrease in physiological threshold (FFR_{angina} and RFR_{angina}). Interaction effects were interpreted using posterior probabilities, with higher values indicating stronger evidence of effect modification by physiological threshold.

Patient enrolment and pre-assessment

Patients were invited to a pre-assessment visit at least two weeks before their scheduled PCI. At this visit, the research team confirmed eligibility and obtained informed consent. Pre-PCI assessments included angina severity graded by the Canadian Cardiovascular Society (CCS) classification, symptoms and quality of life evaluated with the Seattle Angina Questionnaire (SAQ), angina typicality assessed with the Rose Angina Questionnaire, and pain sensitivity measured with the Pain Sensitivity Questionnaire (PSQ).

Patients were also trained to use the dedicated ORBITA-FIRE smartphone application for daily angina reporting.

Smartphone ORBITA-App

The ORBITA-FIRE symptom smartphone application requires the participant to define their angina in their own words and then report the number of episodes of this symptom for each day of the trial. It also requires the participant to report for each week if they experienced angina with 2 activities that were set by the participant at enrolment as triggering their symptoms.

The symptom application approach permits not only a quantitative assessment of the time-course of angina evolution during the follow-up period, but also a time-to-event analysis of occurrence of first angina episode.

Full details regarding development and use of the application have previously published.[33]

Supplemental Figure S2 contains screenshots from the ORBITA-FIRE symptom application.

Supplemental Figure S2: Screenshots from ORBITA-App

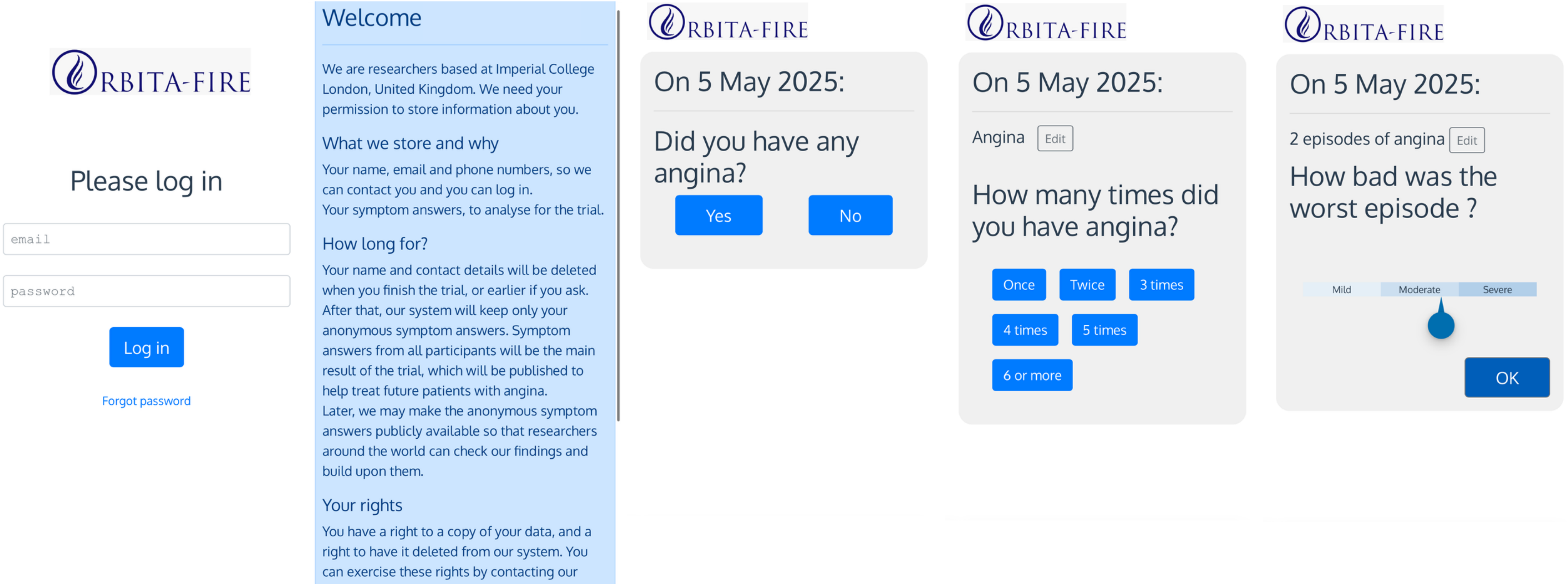

Smart watch

To understand patients' daily physical activity levels and cardiovascular health, they were also provided with an Apple Watch™ during this period. Total energy expenditure, daily step count, peak heart rate, walking heart rate, resting heart and heart rate variability were assessed.

Questionnaires

Seattle Angina Questionnaire

The Seattle Angina Questionnaire, introduced in 1994 as a self-administered, disease specific measure comprising 19 items. This instrument systematically evaluates key domains, including angina frequency, angina stability, physical limitation, quality of life, and treatment satisfaction. SAQ scores range from 0 to 100, with higher scores indicating better health status.[34]

Rose angina questionnaire

The Rose angina questionnaire, initially developed in 1962 as a research instrument for the identification of coronary artery disease, comprises eight questions, seven of which aim to assess the presence of angina and one addressing myocardial infarction.

According to this framework, angina is considered present if the patient experiences chest pain induced either by walking on level or walking uphill, resulting in the patient slowing down or coming to a complete stop, with the pain subsiding within a 10-minute timeframe. Moreover, the specified location of the pain must fall within the regions of the sternum and/or left chest and left arm.[35]

Pain Sensitivity Questionnaire

The Pain Sensitivity Questionnaire (PSQ), developed in 2009, measures perceived pain sensitivity through 14 imagined painful scenarios rated on a 0–10 scale. Three non-painful control items are included as controls but not used in scoring.[36]

Medication prescribing protocol

All medication changes during the study were made by the research team with informed consent from the participant. Decisions were discussed with primary care practitioners as necessary. The medication protocol has previously been described.[4]

1. Participants not already taking the following medications were started on:

Dual antiplatelet therapy:
Standard loading doses was used. Thereafter, aspirin 75 mg once daily with either clopidogrel 75 mg once daily or ticagrelor 90 mg twice daily or prasugrel 5-10 mg once daily, dose adjusted for age and weight, was administered.

Gastrointestinal (GI) protection:
If at high risk of adverse GI effects (based on previous GI ulceration, age or concomitant medications that increase risk), participants were started on a proton pump inhibitor, lansoprazole 30mg once daily, in accordance with NICE guidance on gastro-esophageal reflux disease and dyspepsia in adults (CG184).

Lipid-lowering medication:
Atorvastatin 80 mg once daily was preferred. If participants were already taking lower dose atorvastatin, simvastatin or pravastatin, this was changed to atorvastatin 80 mg once daily. If taking rosuvastatin, this was continued.

2. Other concomitant risk factor modifying medication

Antihypertensives:
Antihypertensives with antianginal properties were stopped. Participants were given a blood pressure monitor and asked to perform home readings. Blood pressure control was monitored by the research team, and if required, antihypertensives were added. Agents without antianginal properties were preferred.

3. Antianginal medication

Regular antianginal medications were stopped on enrolment. All participants were given glyceryl trinitrate spray to be used when necessary. The need for starting regular antianginals was determined by participant preference and patient-reported symptoms.

An individualized protocol for potential introduction of antianginal medications was prepared for each participant by the research team. This protocol was based on the participant's medical history, heart rate, blood pressure and any medication intolerance. The preferred sequence was as follows: Bisoprolol, nifedipine MR, isosorbide mononitrate MR, nicorandil, ranolazine.

Antianginals started prior to PCI were stopped following PCI and re-introduced according to participant preference and symptoms as described above, by a member of the research team.

Supplemental Table S1: Antianginal medication quantification

Common antianginal medications were classified as 1 unit based on the following total daily dosages:

Medication	Total daily dose in mg that constitutes 1 unit
Bisoprolol	5
Atenolol	25
Amlodipine	2.5
Nifedipine	20
Isosorbide mononitrate MR	30
Isosorbide mononitrate SR	25
Diltiazem	120
Nicorandil	20
Ranolazine	750
Ivabradine	5

Cardiac Catheterization Protocol

Physiological measurements and indices

The following measurements were obtained during invasive assessment of coronary physiology.[10,11, 37-42]

Supplemental Table S2: Hemodynamic Indices

Hemodynamic Index	Definition
Aortic Pressure (**Pa**)	Proximal (Aortic) pressure (mmHg)
Distal Pressure (**Pd**)	Distal (Coronary) pressure (mmHg)
Resting Full-cycle Ratio (**RFR**)	A measure of the functional significance of an epicardial stenosis at rest.<br>Calculated as:<br>Pd/Pa at baseline during the wave-free period of the cardiac cycle
Fractional Flow Reserve (**FFR**)	A measure of the functional significance of an epicardial stenosis during maximal hyperemia.<br>Calculated as:<br>Pd/Pa at whole-cycle during pharmacologically induced hyperemia
Coronary Flow Reserve (**CFR**)	A measure of the vasodilatory capacity of the epicardial and microvascular coronary compartments.<br>Calculated as:<br>Resting mean transit time (T_{mnrest}) / hyperemic mean transit time ($T_{mnhyperemia}$)
Index of Microvascular Resistance (**IMR_{corr}**)	A measure of coronary microvascular resistance.<br>Calculated as:<br>P_a x mean transit time (T_{mn}) during pharmacologically induced hyperemia.<br>IMR values will be corrected for the wedge pressure using Yong's formula:<br>P_a x $T_{mnhyperaemia}$ x ([1.35 x P_d/P_a] - 0.32)

Protocol for assessing RFR

Following calibration and normalization of the pressure wire in the aorta, the wire was advanced so that the pressure and temperature sensor was positioned ≥6 cm distal to the ostium of the guide catheter within the target coronary artery. Resting pressure waveforms were recorded simultaneously from the guide catheter (Pa) and the distal coronary segment (Pd). Equalization of Pa and Pd was confirmed at the ostium prior to wire advancement. Resting Pd/Pa was documented, and RFR was measured using the CoroFlow software with continuous pressure recording. The lowest resting Pd/Pa

ratio over the entire cardiac cycle was automatically detected and bookmarked by the system.[10] If RFR was negative (>0.89) and no prior evidence of ischemia was present, patients were not enrolled in the study. If RFR was positive (≤ 0.89), the procedure proceeded to thermodilution-derived assessment of coronary flow and microvascular resistance.

Protocol for assessing CFR/IMR

Prior to thermodilution measurements, the guide catheter was thoroughly flushed with heparinized saline using a 3 mL Luer-lock syringe attached to the manifold, ensuring complete removal of residual contrast to avoid erroneous transit time measurements. The CoroFlow system was placed into CFR/IMR mode, and the temperature sensor was zeroed.

For assessment of mean transit time at rest, boluses of 3 mL room temperature saline were quickly injected by the operator via the three-way tap on the manifold upon instruction from the CoroFlow software. Transit times were measured by the software using thermodilution methodology. Three valid transit time curves were acquired at rest.

Hyperemia was then induced via a central intravenous infusion of adenosine at a rate of 140 mcg/kg/min. Once a stable hyperemic response was observed (defined as a sustained drop in Pd and consistent pressure waveform morphology), three additional 3 mL saline boluses were administered, again timed with prompts from the CoroFlow system. The CFR, CFR-norm (corrected for FFR), IMR, IMR-norm (corrected for FFR), and the resistive reserve ratio (RRR) were then automatically calculated and recorded.[37-40]

Protocol for assessing FFR

With sustained intravenous adenosine infusion ongoing, the CoroFlow system was switched to FFR mode. The FFR value was calculated during stable hyperemia. Following FFR measurement, the adenosine infusion was discontinued, and pressures were monitored to ensure return to pre-hyperemic baseline values.

Collateral Flow Index (CFI) measurement

Our study allowed for the calculation of CFI through complete balloon occlusion of the target vessel post-PCI. A guide catheter was used to measure the aortic pressure proximal to the occlusion (Pa), while the pressure distal to the occluding balloon (P_{occl}) was recorded using the pressure sensor on the intracoronary guidewire. Mean central venous pressure (CPV) was obtained via a pig tail catheter positioned in the right atrium and averaged over the cardiac cycle. CFI was then calculated using[41-42]:

$$CFI = \frac{P_{occl} - CVP}{Pa - CVP}$$

Quantitative coronary angiography (QCA) Analysis

QCA was performed using McKesson Cardiology version 14.0 software. The system was calibrated by tracing catheter segments with known diameters to ensure accurate scaling. Stenotic regions were then outlined to calculate vessel dimensions, including percentage diameter stenosis, with higher values indicating more severe narrowing. Measurements were performed independently by three investigators, and the final values were obtained by averaging their results. All investigators were blinded to the patients' FFR and RFR thresholds for angina during the analysis.

Exercise Protocol

Supplemental Figure S3: Balloon inflations during supine exercise

This image shows the catheterization laboratory setup during high-intensity exercise testing in ORBITA-FIRE. After positioning a non-compliant balloon within the stented segment, the guide catheter was disengaged to minimize vessel trauma and ensure accurate, undamped aortic pressure measurement. Patients began cycling on a supine ergometer at 40 RPM, starting with an initial workload of 40 W and increasing by 20 W per minute until the heart rate that previously triggered angina at baseline was achieved, in this example, 112 bpm caused chest pain pre-PCI. Once this target heart rate was achieved, stepwise balloon inflations were performed, reducing the distal-to-aortic pressure ratio (Pd/Pa) in increments of approximately 0.02 every 5-10 seconds. The high-intensity angina inflation pressure Pd/Pa was recorded at the first onset of chest pain.

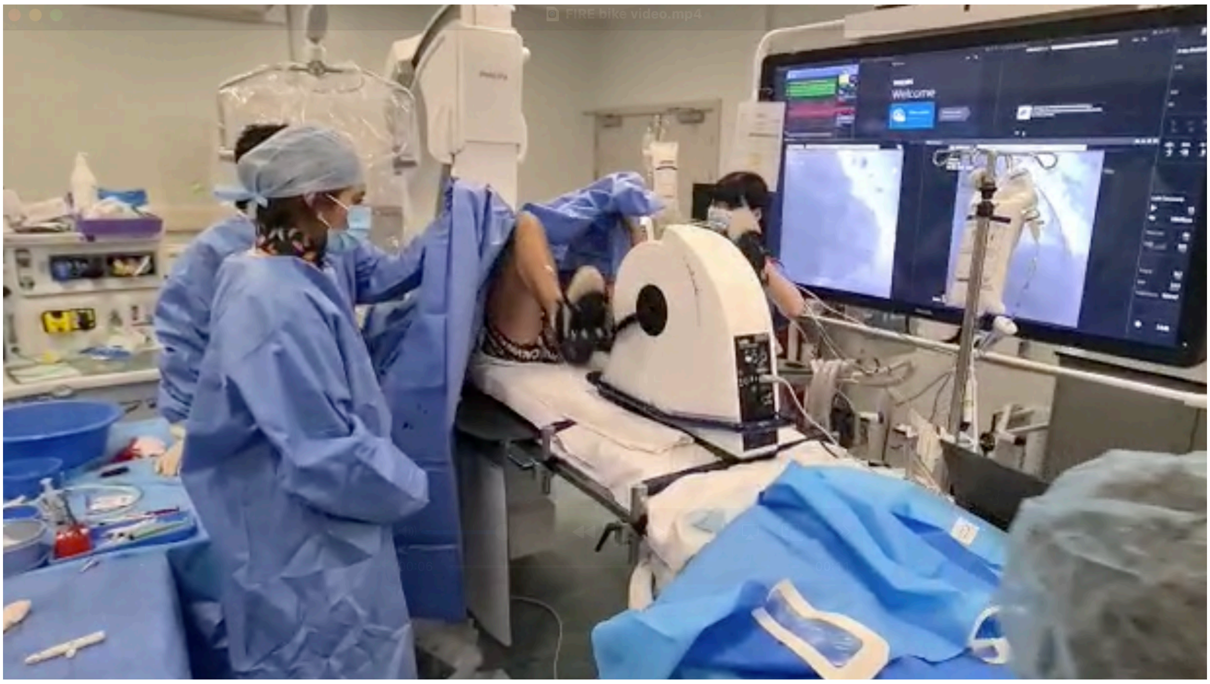

Supplemental Figure S4: Physiology screenshots with exercise

This panel illustrates stepwise low-pressure inflations performed within a treated segment of the left anterior descending artery, where a 3.5 × 20 mm drug-eluting stent had been implanted. A 3.0 × 15 mm non-compliant balloon was positioned in-stent and sequentially inflated in small increments. Each inflation aimed to reduce the distal-to-aortic pressure ratio (Pd/Pa) by approximately 0.02 every 5-10 seconds. In this example, the patient's post-PCI Pd/Pa was initially 0.94 (**Figure S4a**), and the first onset of chest pain occurred at a Pd/Pa of 0.38 (**Figure S4b**). The entire process took 240 seconds from the first inflation to the onset of angina. This Pd/Pa threshold of 0.38 was subsequently reproduced during blinded testing to confirm the symptom threshold against a placebo inflation and was also used to calculate both FFR_{angina} and RFR_{angina} for this symptom-inducing stenosis.

Figure S4a

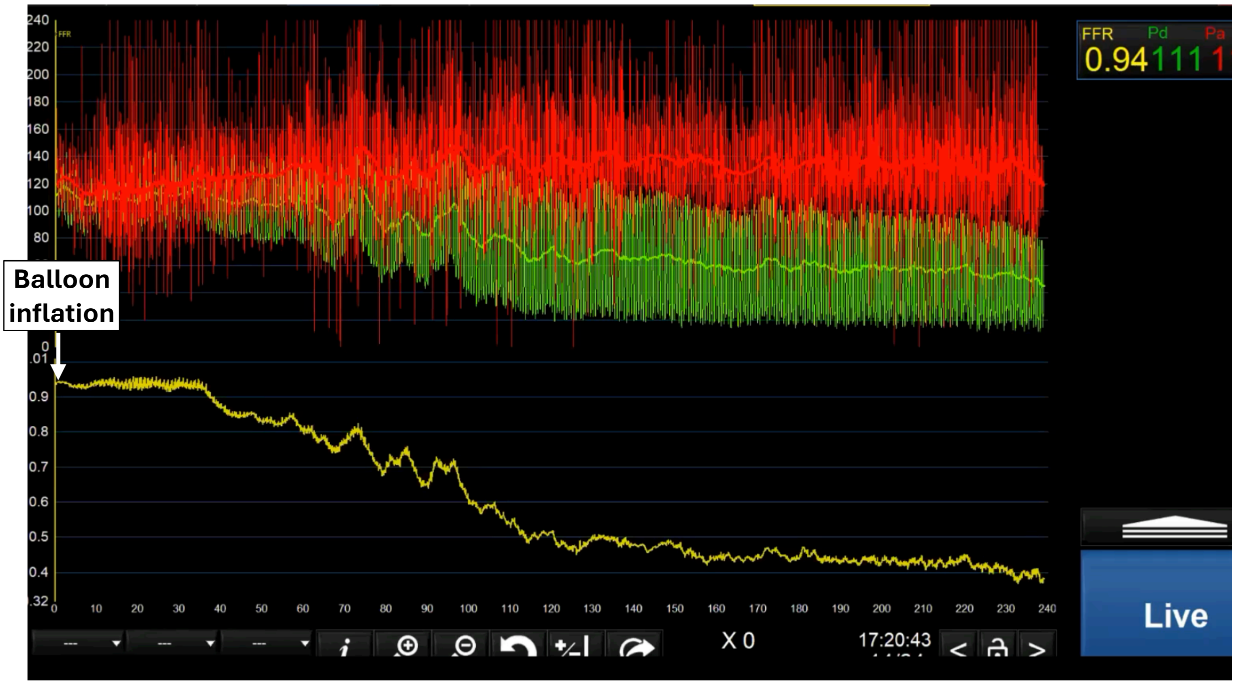

Figure S4b

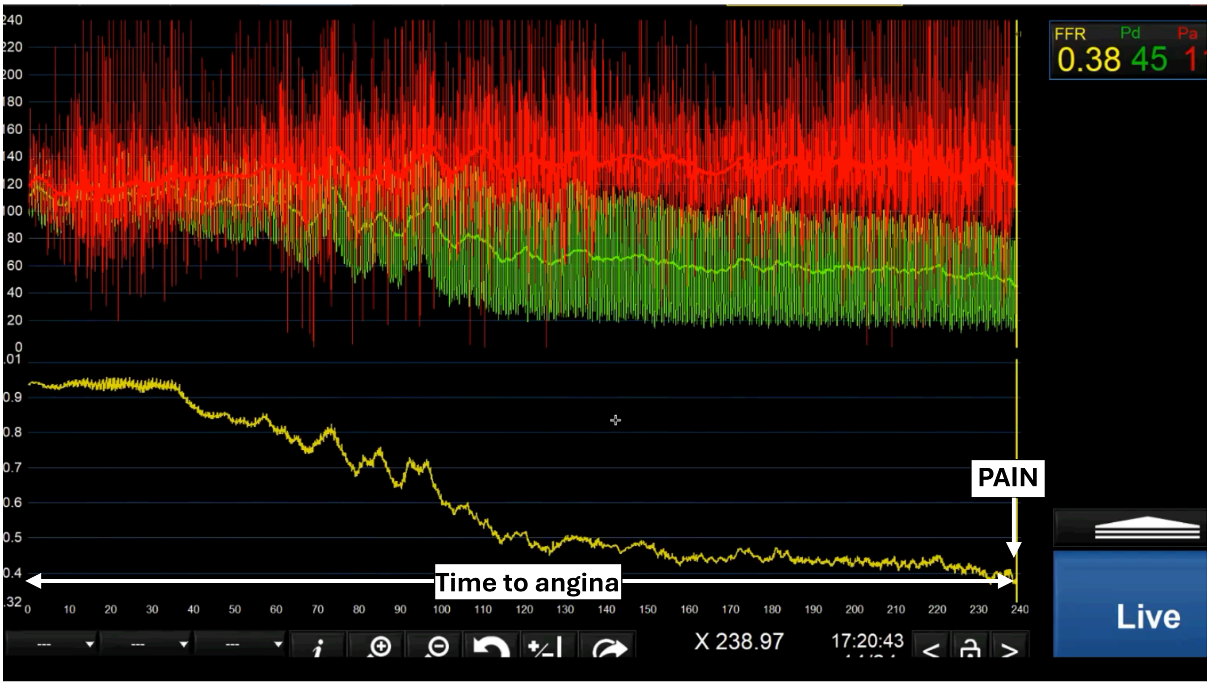

Supplementary results

Supplemental Table S3: Vessels assessed and vessel location

Vessel type	n=65
Left anterior descending artery	47 (72.3)
Proximal	39 (60.0)
Mid	8 (12.3)
Distal	0 (0.0)
Right coronary	12 (18.5)
Proximal	2 (3.0)
Mid	9 (13.8)
Distal	1 (1.5)
Circumflex artery	3 (4.6)
First diagonal artery	2 (3.1)
Obtuse marginal artery	1 (1.5)

Values are n (%) unless otherwise indicated. Percentages may not total 100 because of rounding.

Supplemental Table S4: Left anterior descending (LAD) vs non-LAD

LAD vs Non-LAD	P-value
**Rest**	
FFR_{angina}	0.757
RFR_{angina}	0.071
**Low-intensity exercise**	
FFR_{angina}	0.416
RFR_{angina}	0.616
**High-intensity exercise**	
FFR_{angina}	0.757
RFR_{angina}	0.616

Supplemental Table S5: Proximal vs non-proximal LAD

**Proximal vs non-proximal LAD**	**P-value**
**Rest**	
FFR_{angina}	0.373
RFR_{angina}	0.338
**Low-intensity exercise**	
FFR_{angina}	0.726
RFR_{angina}	0.642
**High-intensity exercise**	
FFR_{angina}	0.987
RFR_{angina}	0.642

Supplemental Table S6: Pre-PCI peak heart rates (per patient)

**Heart rate**	**Mean (SD) - BPM**
Pre-PCI	108.2 (16.4)
Rest (no exercise)	66.9 (10.6)
Low-intensity exercise (n=62)	89.7 (15.3)
High-intensity exercise	107.8 (15.9)

Supplemental Table S7: Lactates across all groups (per patient)

**Lactate**	**Mean (SD)**
Pre-PCI (n=60)	4.0 (2.1)
Rest (no exercise) (n=61)	1.1 (0.4)
Low-intensity exercise (n=61)	3.0 (1.5)
High-intensity exercise (n=61)	4.3 (2.0)

Logistic regression modeling of the patient-individualized angina thresholds

Supplemental figure S5: FFR_{angina} threshold at rest

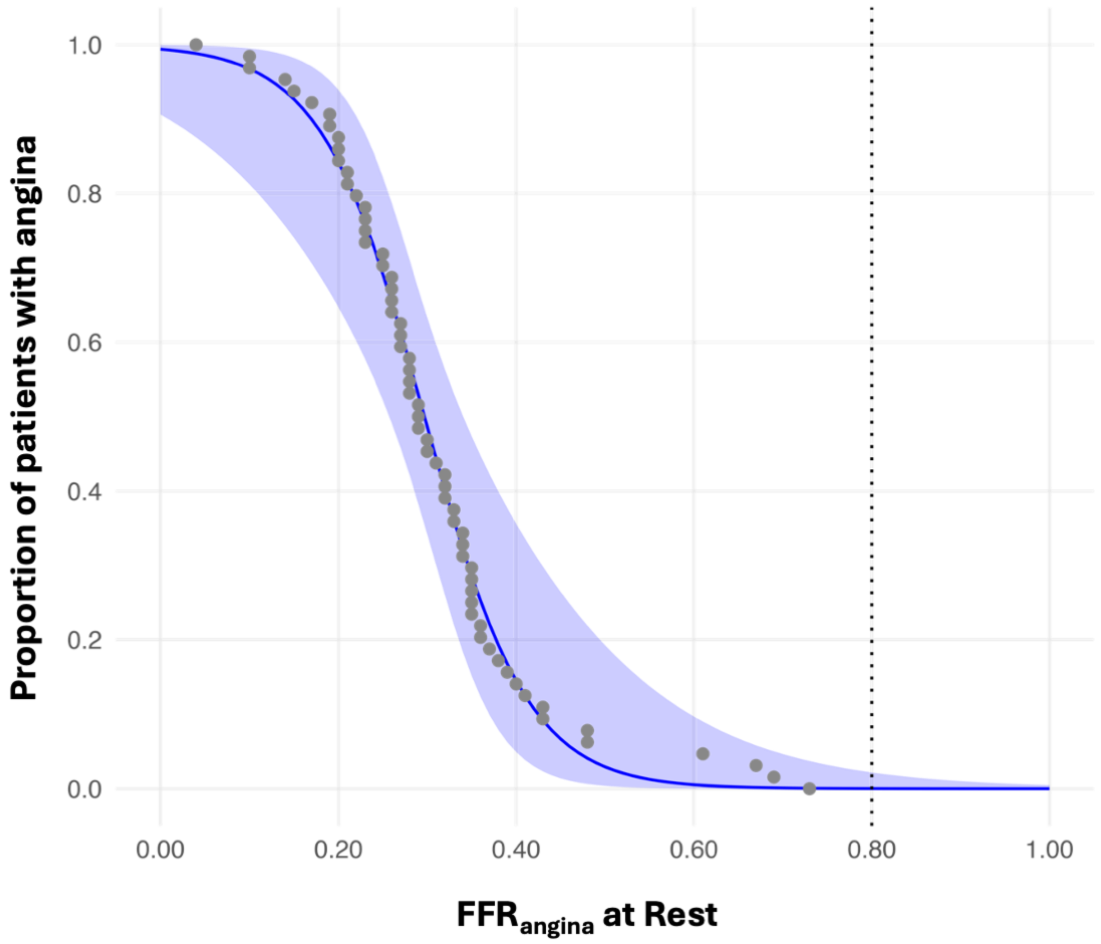

FFR_{angina} threshold at Rest	Proportion of patients with angina
0.80	<0.001%
0.36	25%
0.30	50%
0.23	75%

At rest, an FFR_{angina} of 0.80 corresponded to less than 0.001% of patients having angina. The proportion of patients expected to have angina increased to 25% at an FFR_{angina} of 0.36, 50% at 0.30, and 75% at 0.23.

Supplemental figure S6: RFR_{angina} threshold at rest

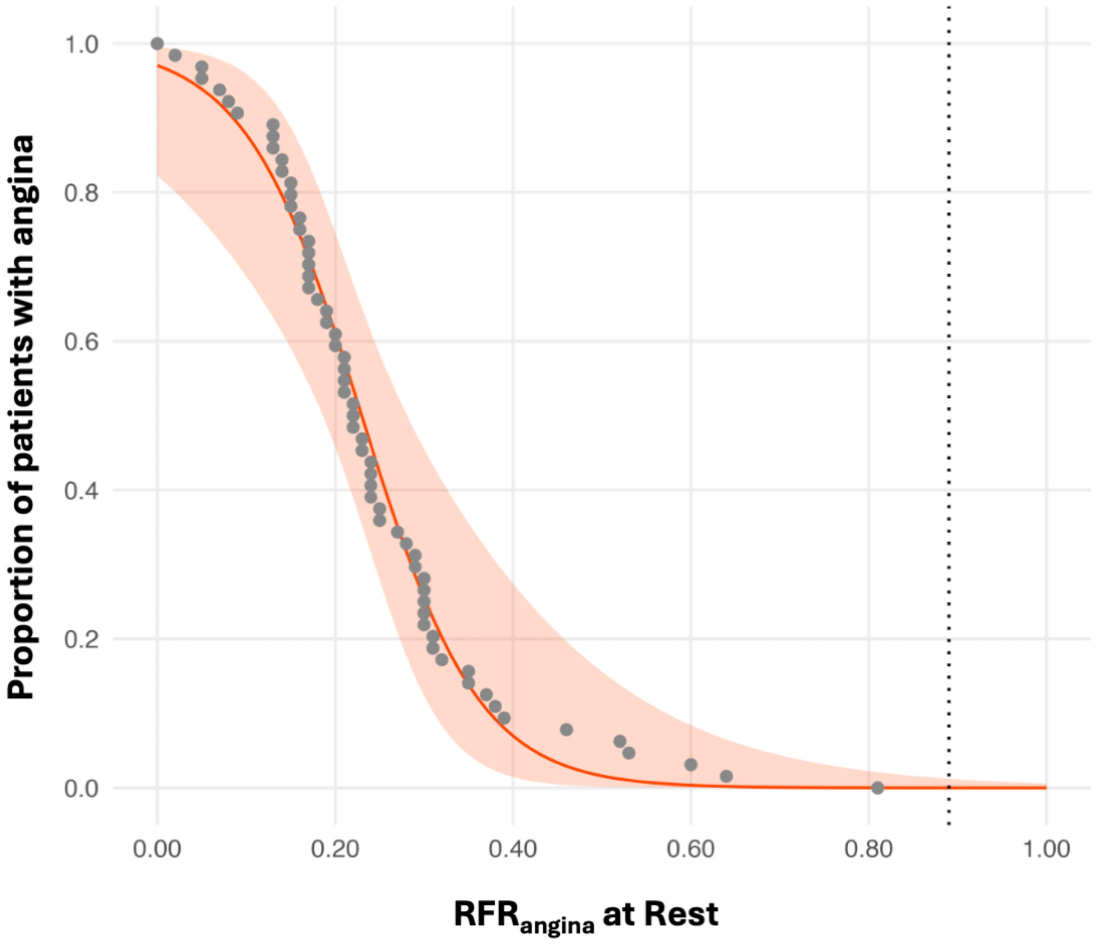

RFR_{angina} threshold at Rest	Proportion of patients with angina
0.89	<0.001%
0.30	25%
0.23	50%
0.16	75%

At rest, an RFR_{angina} of 0.89 corresponded to less than 0.001% of patients having angina. The proportion of patients expected to have angina increased to 25% at an RFR_{angina} of 0.30, 50% at 0.23, and 75% at 0.16.

Supplemental figure S7: FFR_{angina} threshold with low-intensity exercise

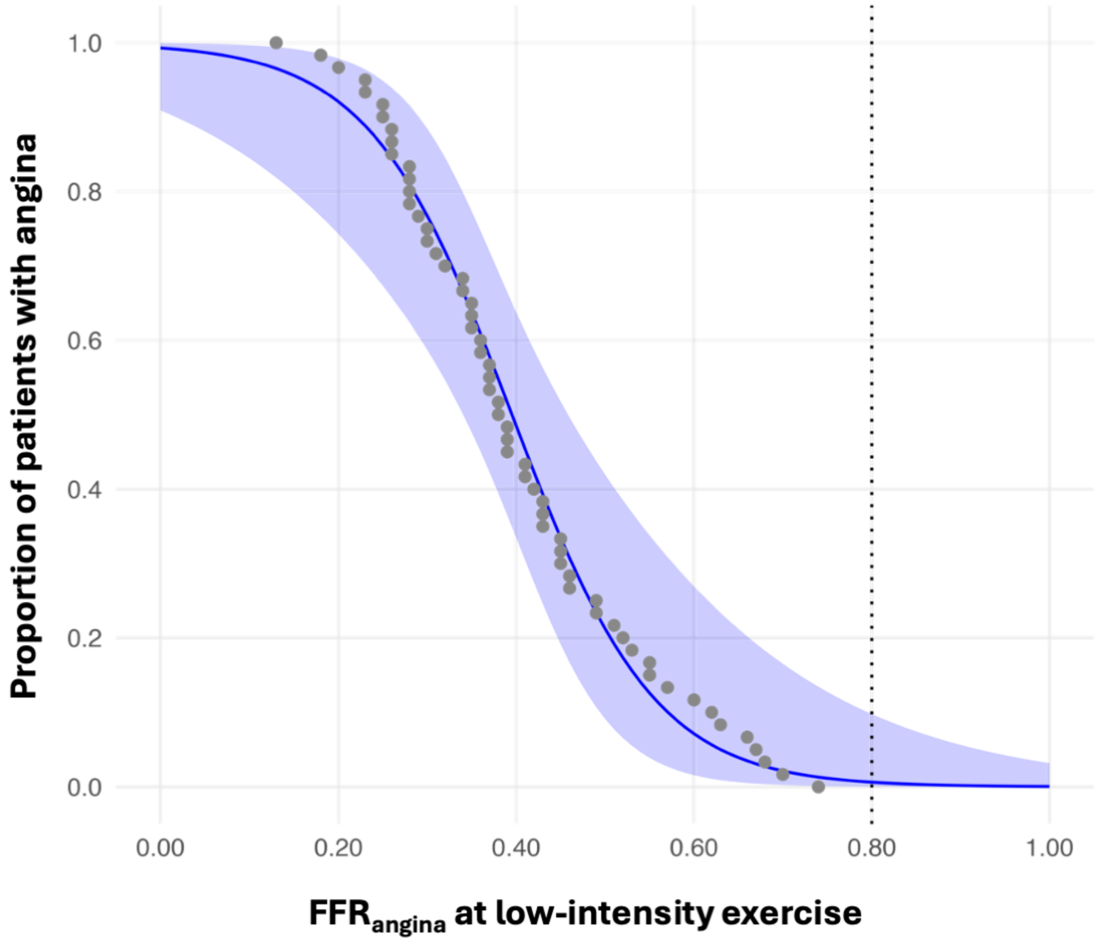

FFR_{angina} threshold at low-intensity exercise	Proportion of patients with angina
0.80	0.006%
0.48	25%
0.40	50%
0.31	75%

During low-intensity exercise, an FFR_{angina} of 0.80 corresponded to 0.006% of patients having angina. The proportion of patients expected to have angina increased to 25% at an FFR_{angina} of 0.48, 50% at 0.40, and 75% at 0.31.

Supplemental figure S8: RFR_{angina} threshold with low-intensity exercise

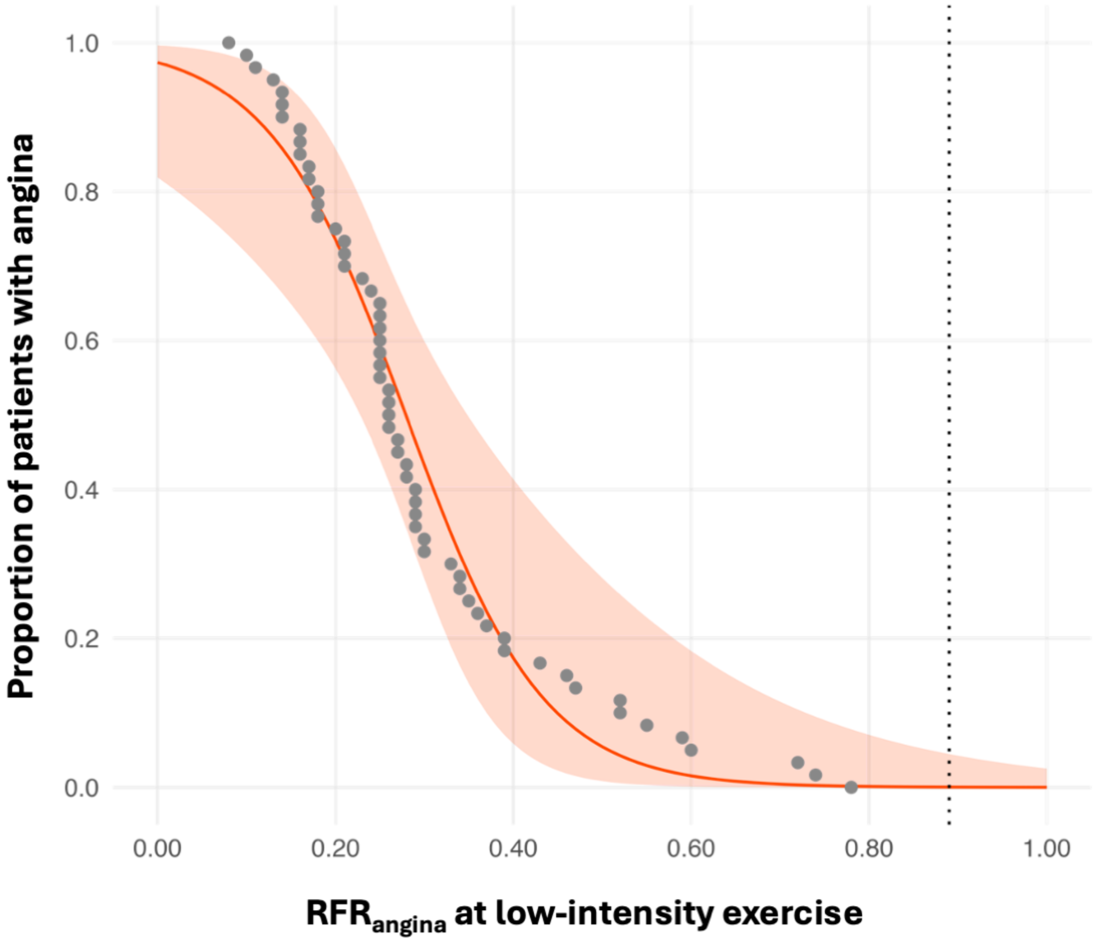

RFR_{angina} threshold at low-intensity exercise	Proportion of patients with angina
0.89	<0.001%
0.53	25%
0.28	50%
0.19	75%

During low-intensity exercise, an RFR_{angina} of 0.89 corresponded to less than 0.001% of patients having angina. The proportion of patients expected to have angina increased to 25% at an RFR_{angina} of 0.53, 50% at 0.28, and 75% at 0.19.

Supplemental figure S9: FFR_{angina} threshold with high-intensity exercise

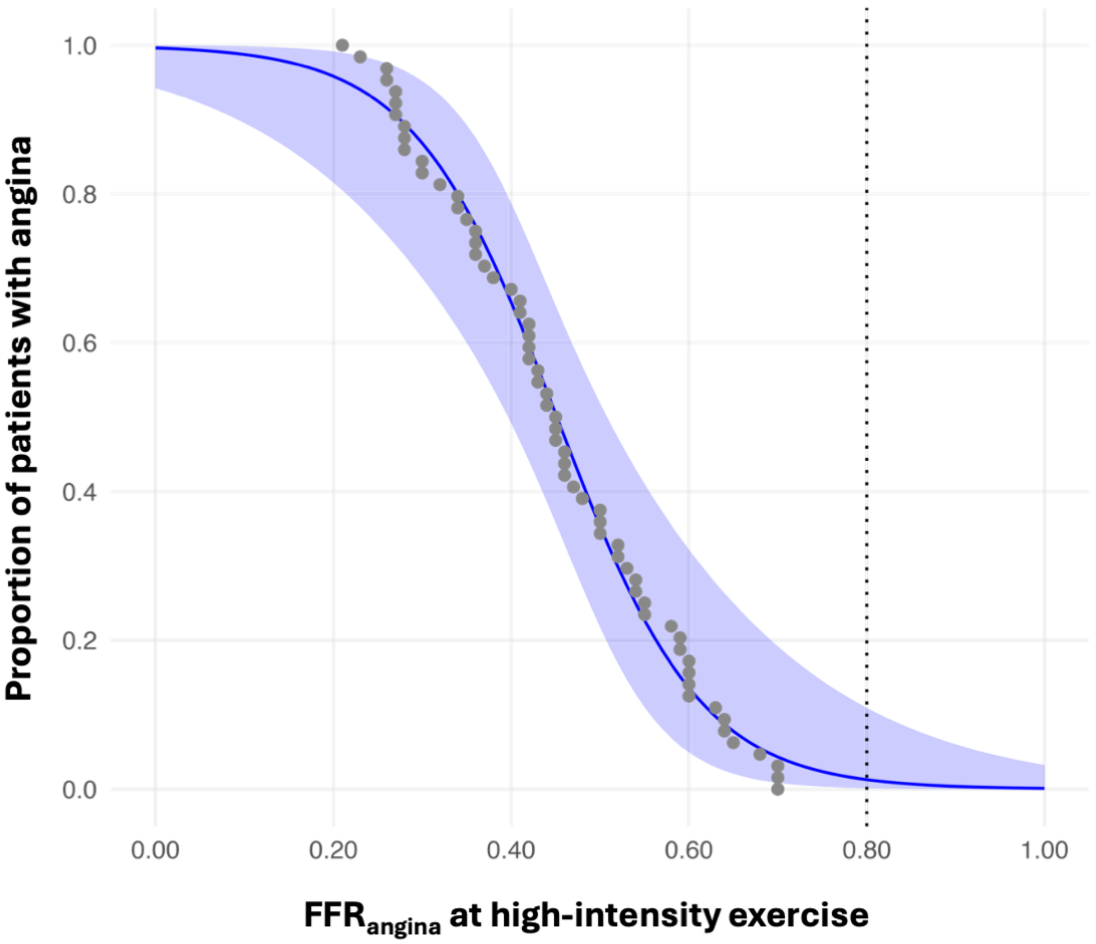

FFR_{angina} threshold at high-intensity exercise	Proportion of patients with angina
0.80	0.01%
0.54	25%
0.45	50%
0.36	75%

During high-intensity exercise, an FFR_{angina} of 0.80 corresponded to 0.01% of patients having angina. The proportion of patients expected to have angina increased to 25% at an FFR_{angina} of 0.55, 50% at 0.45, and 75% at 0.36.

Supplemental figure S10: RFR_{angina} threshold with high-intensity exercise

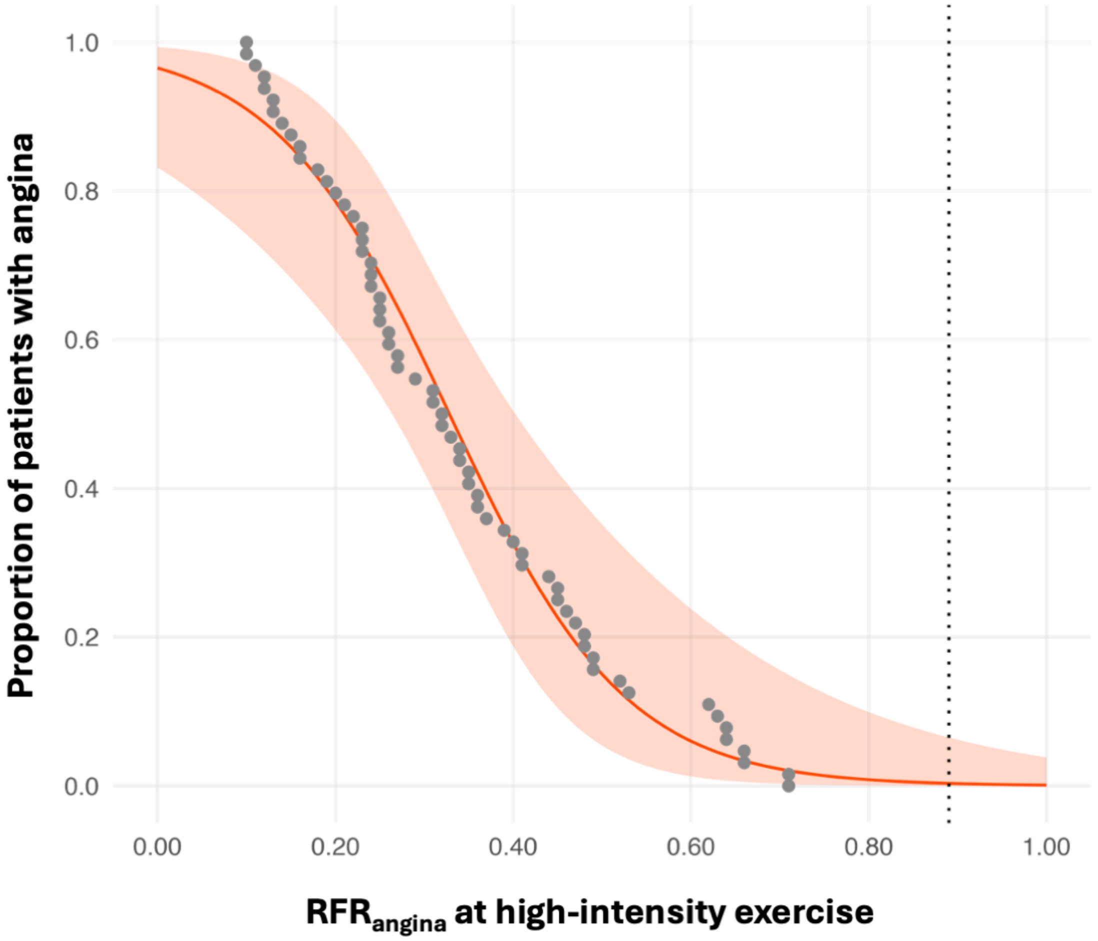

RFR_{angina} threshold at high-intensity exercise	Proportion of patients with angina
0.89	0.003%
0.43	25%
0.33	50%
0.22	75%

During high-intensity exercise, an RFR_{angina} of 0.89 corresponded to less than 0.003% of patients having angina. The proportion of patients expected to have angina increased to 25% at an RFR_{angina} of 0.43, 50% at 0.33, and 75% at 0.22.

Effect of PCI with the primary endpoints

Angina symptom frequency

Supplemental Table S8: Comparison of angina frequency burden at baseline

Angina thresholds	Angina score Below the median threshold	Angina score above the median threshold	Difference (IQR)	Significance
**Rest**				
FFR_{angina}	1.42	0.95	0.47 (0.42 to 0.51)	<0.001
RFR_{angina}	1.05	1.32	-0.27 (-0.31 to -0.22)	<0.001
**Low-intensity exercise**				
FFR_{angina}	1.30	1.10	0.20 (0.18 to 0.27)	<0.001
RFR_{angina}	1.10	1.27	-0.17 (-0.22 to -0.12)	<0.001
**High-intensity exercise**				
FFR_{angina}	1.38	0.98	0.40 (0.3 to 0.44)	<0.001
RFR_{angina}	1.23	1.14	0.09 (0.0 to 0.14)	<0.001

Supplemental Table S9: Ability of angina thresholds to predict benefit with PCI

Daily angina episodes	25th centile	75th centile	Probability of greater benefit in a patient with a lower angina threshold (Pr)
**Rest**			
**FFR_{angina}**	**0.23**	**0.35**	
Odds of improvement	2.28 (1.83 to 2.91)	1.74 (1.42 to 2.15)	
Odds ratio	0.27 (0.11 to 0.42)		>0.999
**RFR_{angina}**	**0.16**	**0.30**	
Odds of improvement	1.75 (1.41 to 2.14)	2.20 (1.73 to 2.74)	
Odds ratio	-0.23 (-0.37 to -0.09)		>0.999
**Low-intensity exercise**			
**FFR_{angina}**	**0.30**	**0.48**	
Odds of improvement	3.25 (2.55 to 4.17)	1.90 (1.53 to 2.40)	
Odds ratio	0.55 (0.37 to 0.74)		>0.999
**RFR_{angina}**	**0.18**	**0.36**	
Odds of improvement	3.65 (2.81 to 4.74)	2.50 (1.95 to 3.23)	
Odds ratio	0.32 (0.12 to 0.51)		>0.999
**High-intensity exercise**			
**FFR_{angina}**	**0.36**	**0.55**	
Odds of improvement	3.50 (2.73 to 4.55)	2.14 (1.72 to 2.63)	
Odds ratio	0.49 (0.33 to 0.66)		>0.999
**RFR_{angina}**	**0.23**	**0.46**	
Odds of improvement	3.03 (2.38 to 3.87)	2.05 (1.65 to 2.59)	
Odds ratio	0.37 (0.18 to 0.55)		>0.999

Supplemental Figure S11: FFR_{angina} (at rest) and angina symptom score

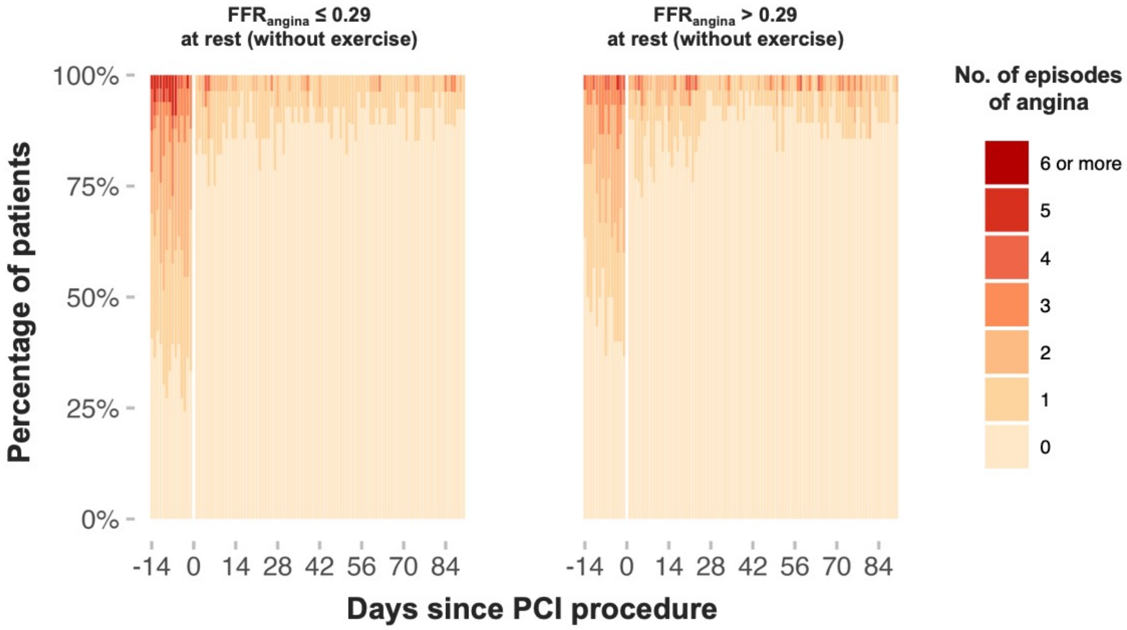

Supplemental Figure S12: FFR_{angina} (at rest) and placebo-controlled impact of PCI

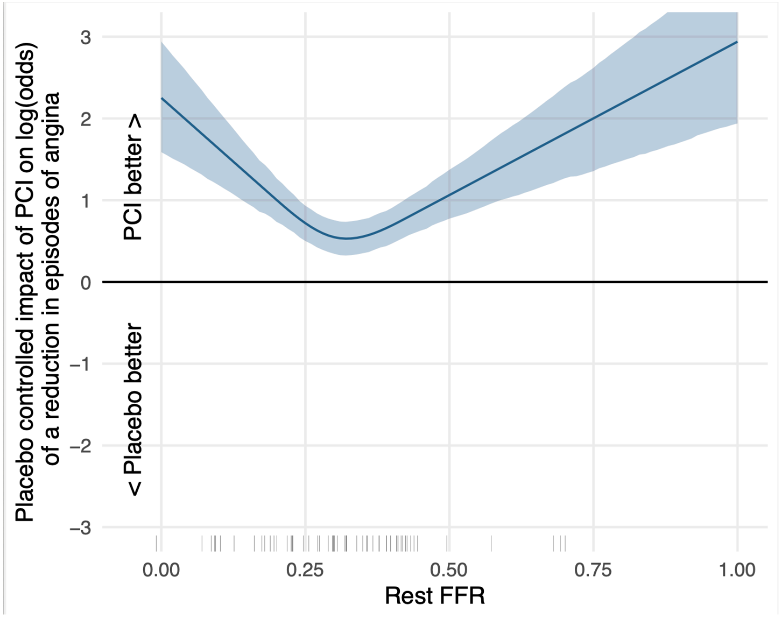

Regression model and coefficients for FFR_{angina} (at rest)

Bayesian Constrained Partial Proportional Odds Ordinal Logistic Model

Dirichlet Priors With Concentration Parameter 0.392 for Intercepts

```
blrm(formula = symptom_frequency_num ~ rcs(symptom_frequency_num_lag1,
    c(1, 3, 5)) + rcs(day_num_m2, 3) + rcs(rest_ffr, 3) + rcs(symptom_frequency_num_pre_mean,
    4), ppo = ~day_num_m2, cppo = function(y) y, data = main_analysis_d,
    pcontrast = pcon, iter = 5000, chains = 4, refresh = 100,
    progress = file.path(output_dir, "res1.progress.txt"), loo = FALSE,
    ppairs = NULL, method = "sampling", file = file.path(output_dir,
        "res1.blrm.rds"))
```

Frequencies of Responses

```
   0    1    2    3    4
4956  529  124   27    9
```

Frequencies of Missing Values Due to Each Variable

```
         symptom_frequency_num     symptom_frequency_num_lag1
                            34                             36
                    day_num_m2                       rest_ffr
                             0                              0
symptom_frequency_num_pre_mean
                            22
```

	Mixed Calibration/ Discrimination Indexes	Discrimination Indexes	Rank Discrim. Indexes
Obs 5645	B 0.077 [0.076, 0.078]	g 1.056 [0.962, 1.167]	C 0.77 [0.757, 0.784]
Draws 10000		g_p 0.124 [0.118, 0.132]	D_{xy} 0.54 [0.513, 0.567]
Chains 4		EV 0.272 [0.248, 0.297]	
Time 191.8s		v 1.306 [1.131, 1.487]	
p 9		vp 0.029 [0.026, 0.032]	

	Mean β	Median β	S.E.	Lower	Upper	Pr(β>0)	Symmetry
y≥1	-3.6521	-3.6525	0.3780	-4.4112	-2.9325	0.0000	0.99
y≥2	-5.4009	-5.3993	0.3958	-6.1935	-4.6476	0.0000	0.98
y≥3	-6.7941	-6.7927	0.4409	-7.6991	-5.9725	0.0000	0.97
y≥4	-7.9937	-7.9810	0.5475	-9.0354	-6.8989	0.0000	0.94
symptom_frequency_num_lag1	2.0508	2.0502	0.0847	1.8873	2.2171	1.0000	1.00
symptom_frequency_num_lag1'	-2.6485	-2.6371	0.5716	-3.7758	-1.5307	0.0000	0.94
day_num_m2	-0.0136	-0.0136	0.0035	-0.0203	-0.0066	0.0000	1.00
day_num_m2'	0.0238	0.0238	0.0034	0.0170	0.0302	1.0000	0.98
rest_ffr	6.2381	6.2213	1.2200	3.8039	8.5773	1.0000	1.03
rest_ffr'	-7.2657	-7.2556	1.2464	-9.6930	-4.8370	0.0000	0.98
symptom_frequency_num_pre_mean	-0.5538	-0.5594	0.3568	-1.2669	0.1157	0.0606	1.01
symptom_frequency_num_pre_mean'	3.7447	3.7496	1.6603	0.5792	6.9203	0.9884	0.99
symptom_frequency_num_pre_mean''	-8.2973	-8.2931	3.6536	-15.4926	-1.4911	0.0113	1.01
day_num_m2 x f(y)	-0.0057	-0.0056	0.0014	-0.0083	-0.0029	0.0000	0.98

Contrasts Given Priors

```
[1] list(c1 = list(rest_ffr = 0.36), c2 = list(rest_ffr = 0.55),
[2]     contrast = expression(c1 - c2), sd = 0.842807127883599)
```

Supplementary figure S13: coefficient density plots: FFR_{angina} (at rest)

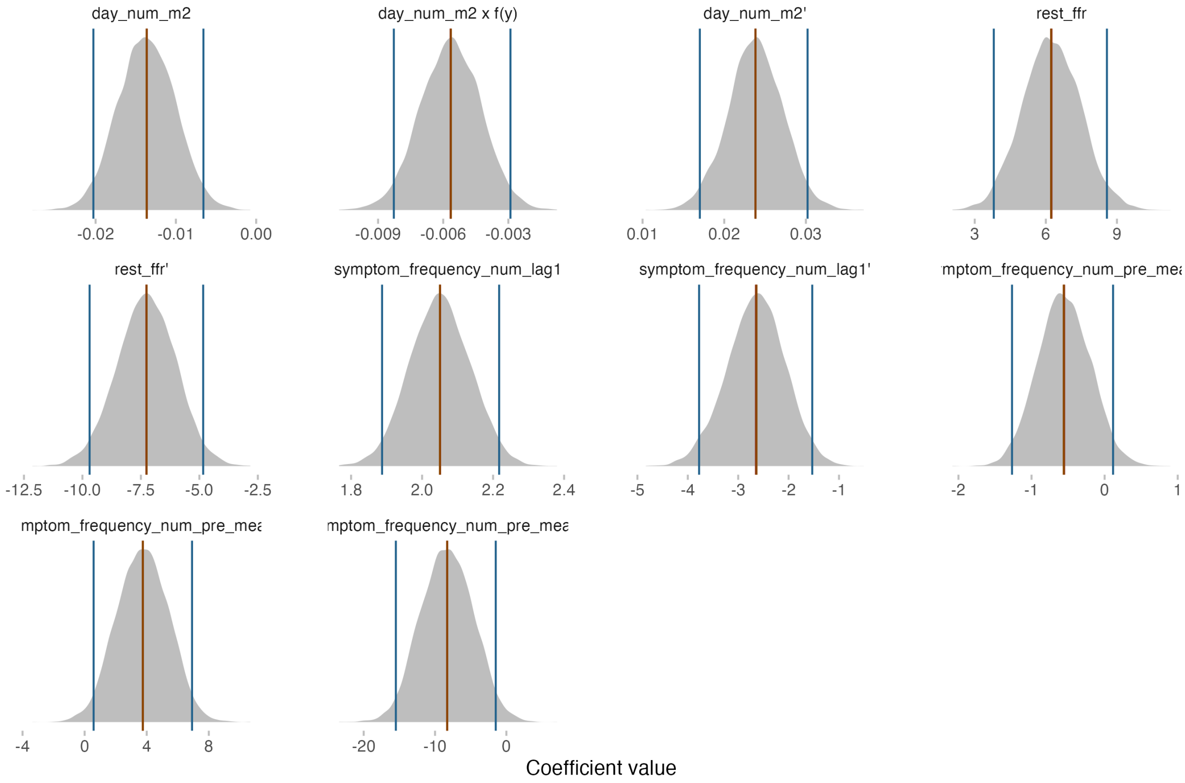

Supplementary figure S14: chain plot of MCMC draws for FFR_{angina} (at rest)

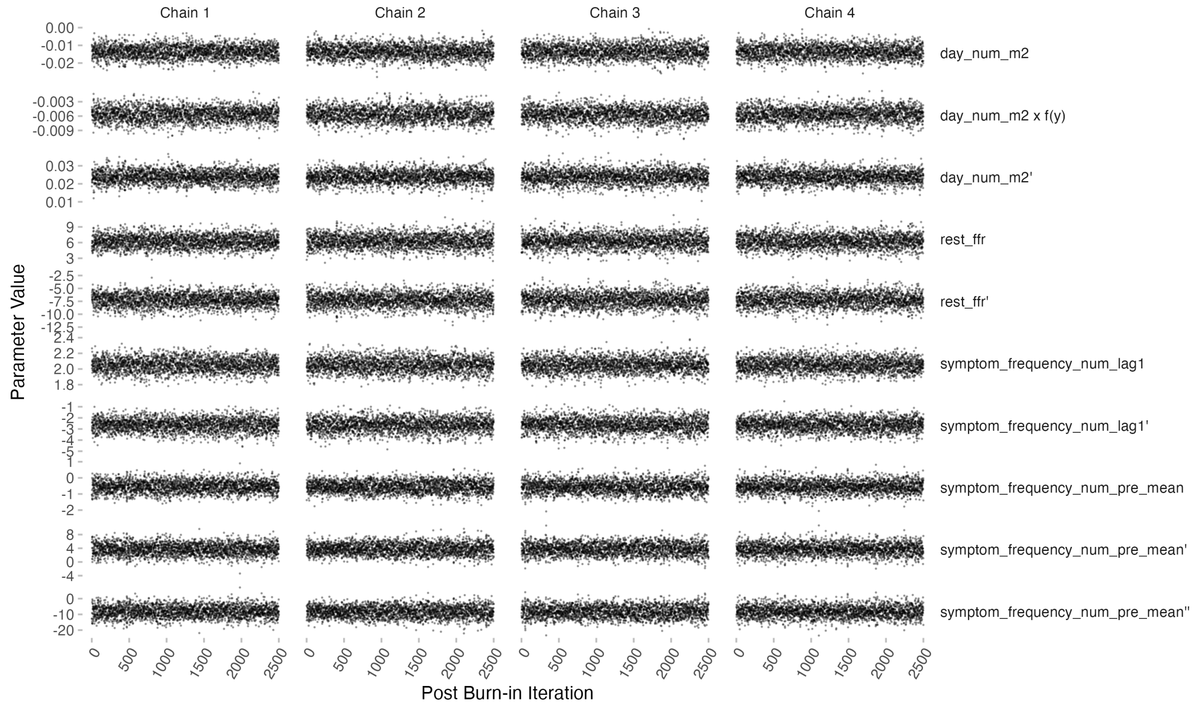

Supplemental Figure S15: RFR_{angina} (at rest) and angina symptom score

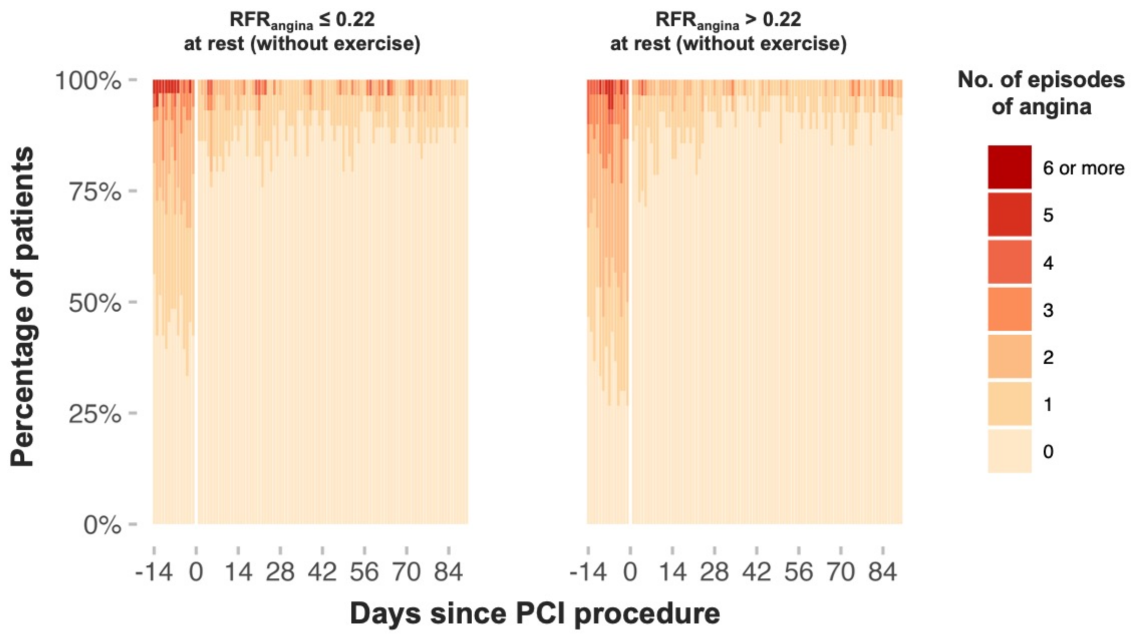

Supplemental Figure S16: RFR_{angina} (at rest) and placebo-controlled impact of PCI

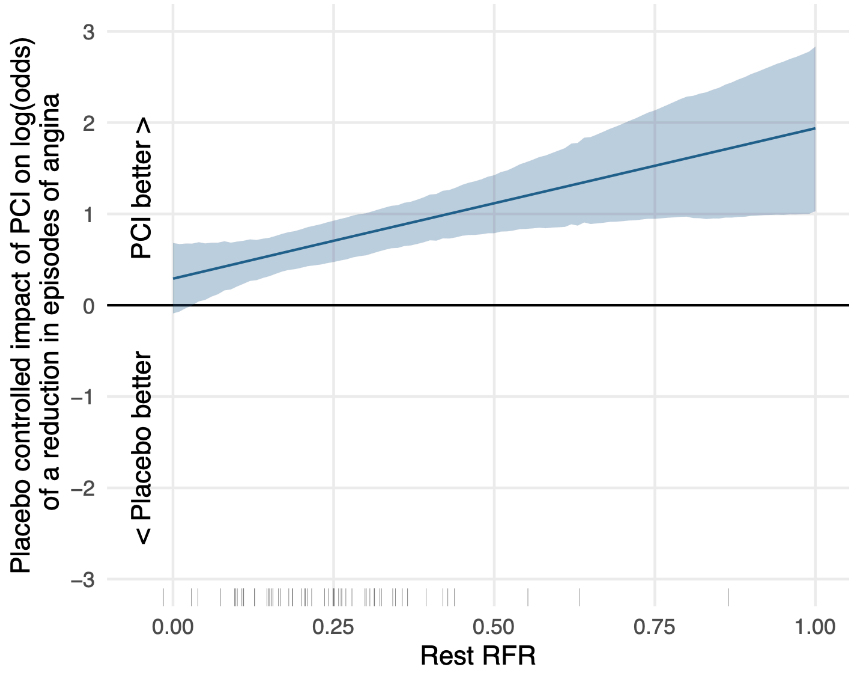

Regression model and coefficients for RFR_{angina} (at rest)

Bayesian Constrained Partial Proportional Odds Ordinal Logistic Model

Dirichlet Priors With Concentration Parameter 0.392 for Intercepts

```
blrm(formula = symptom_frequency_num ~ rcs(symptom_frequency_num_lag1,
    c(1, 3, 5)) + rcs(day_num_m2, 3) + rcs(rest_rfr, 3) + rcs(symptom_frequency_num_pre_mean,
    4), ppo = ~day_num_m2, cppo = function(y) y, data = main_analysis_d,
    pcontrast = pcon, iter = 5000, chains = 4, refresh = 100,
    progress = file.path(output_dir, "res1.progress.txt"), loo = FALSE,
    ppairs = NULL, method = "sampling", file = file.path(output_dir,
        "res1.blrm.rds"))
```

Frequencies of Responses

```
   0    1    2    3    4
4956  529  124   27    9
```

Frequencies of Missing Values Due to Each Variable

```
         symptom_frequency_num     symptom_frequency_num_lag1
                            34                             36
                    day_num_m2                       rest_rfr
                             0                              0
symptom_frequency_num_pre_mean
                            22
```

	Mixed Calibration/ Discrimination Indexes	Discrimination Indexes	Rank Discrim. Indexes
Obs 5645	B 0.077 [0.076, 0.078]	g 0.964 [0.875, 1.061]	C 0.75 [0.737, 0.763]
Draws 10000		g_p 0.121 [0.113, 0.128]	D_{xy} 0.501 [0.473, 0.526]
Chains 4		EV 0.272 [0.241, 0.295]	
Time 176.8s		v 1.209 [1.021, 1.366]	
p 9		vp 0.029 [0.026, 0.032]	

	Mean β	Median β	S.E.	Lower	Upper	Pr(β>0)	Symmetry
y≥1	-1.8155	-1.8098	0.2690	-2.3531	-1.3015	0.0000	0.98
y≥2	-3.5679	-3.5654	0.2885	-4.1091	-2.9855	0.0000	0.99
y≥3	-4.9706	-4.9656	0.3451	-5.6499	-4.3114	0.0000	0.98
y≥4	-6.1832	-6.1674	0.4735	-7.1263	-5.2779	0.0000	0.92
symptom_frequency_num_lag1	2.0979	2.0984	0.0831	1.9306	2.2569	1.0000	1.01
symptom_frequency_num_lag1'	-2.9345	-2.9236	0.5826	-4.0506	-1.7868	0.0000	0.95
day_num_m2	-0.0131	-0.0131	0.0036	-0.0198	-0.0060	0.0003	1.03
day_num_m2'	0.0229	0.0229	0.0034	0.0165	0.0297	1.0000	0.97
rest_rfr	-1.6608	-1.6640	0.9713	-3.5224	0.3001	0.0479	1.03
rest_rfr'	0.0194	0.0390	1.4579	-2.8627	2.8572	0.5129	0.96
symptom_frequency_num_pre_mean	-0.3552	-0.3595	0.3553	-1.0372	0.3505	0.1560	1.01
symptom_frequency_num_pre_mean'	2.3314	2.3344	1.6507	-0.9711	5.5078	0.9189	1.00
symptom_frequency_num_pre_mean''	-4.9750	-4.9836	3.6265	-12.4090	1.8913	0.0864	1.00
day_num_m2 x f(y)	-0.0055	-0.0055	0.0014	-0.0084	-0.0030	0.0000	0.98

Contrasts Given Priors

```
[1] list(c1 = list(rest_rfr = 0.36), c2 = list(rest_rfr = 0.55),
[2]     contrast = expression(c1 - c2), sd = 0.842807127883599)
```

Supplementary figure S17: coefficient density plots: RFR$_{angina}$ (at rest)

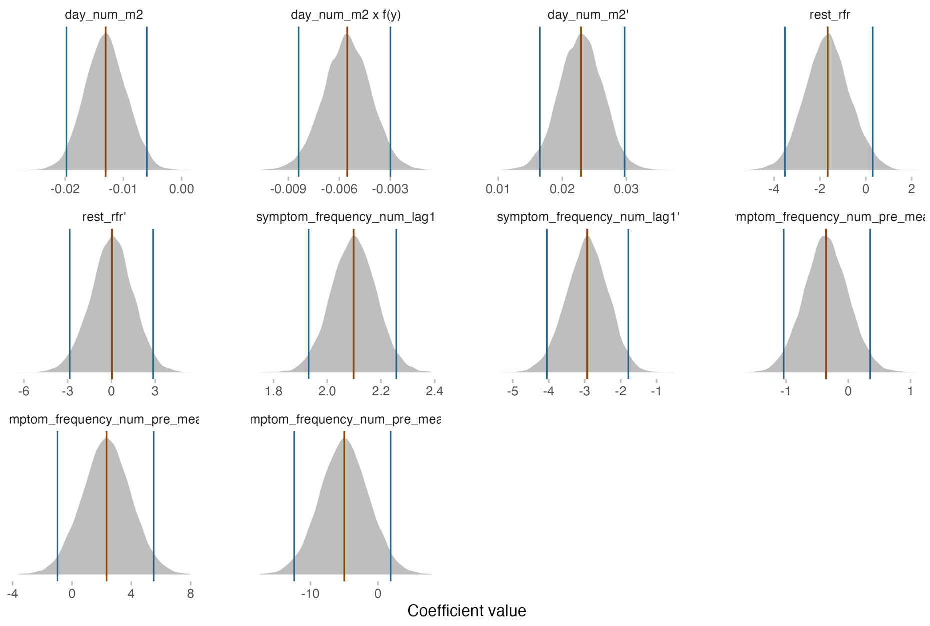

Supplementary figure S18: chain plot of MCMC draws for RFR_{angina} (at rest)

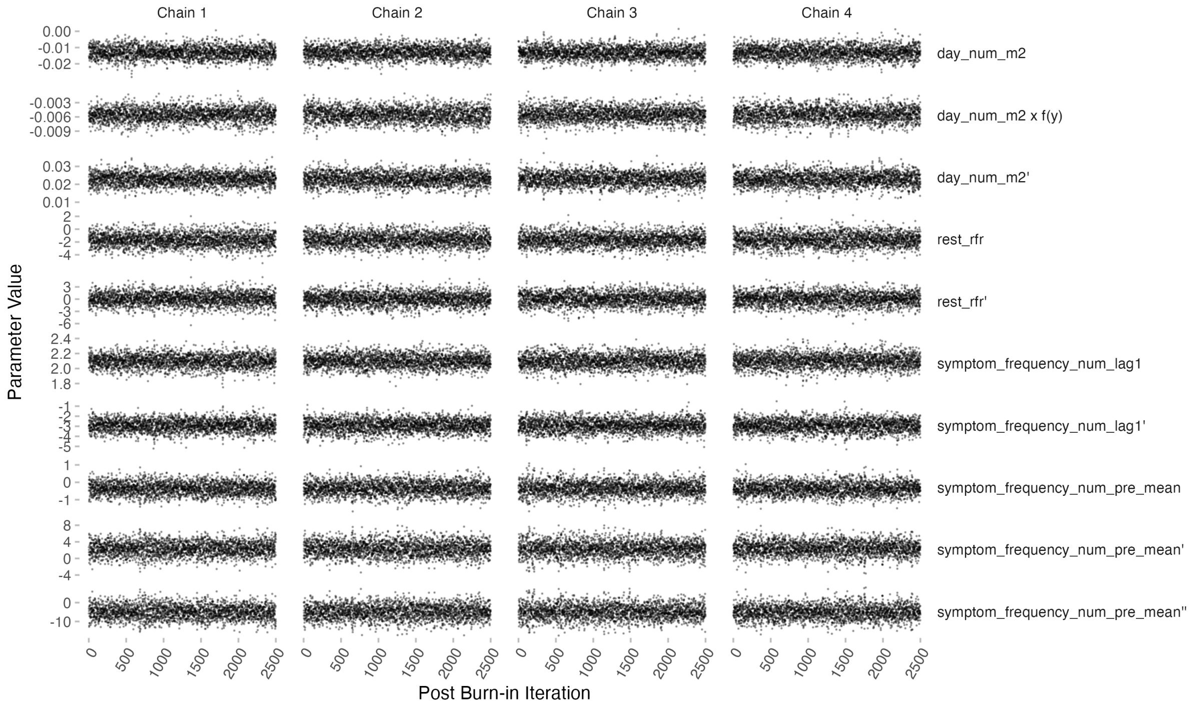

Supplemental Figure S19: FFR_{angina} (at low-intensity exercise) and angina symptom score

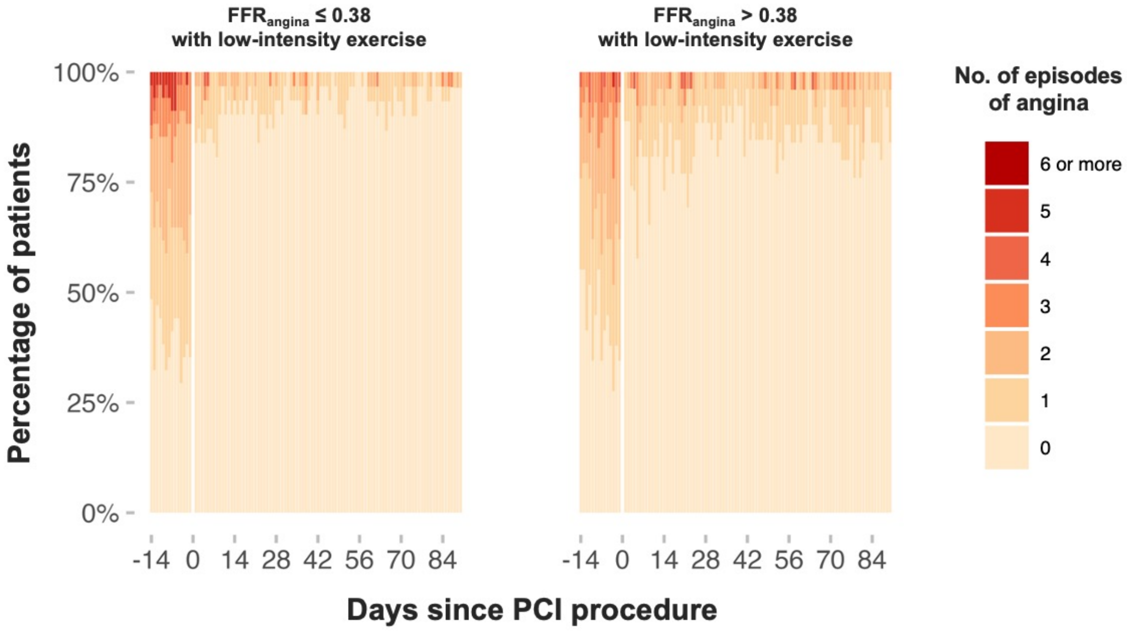

Supplemental Figure S20: FFR$_{angina}$ (at low-intensity exercise) and placebo-controlled impact of PCI

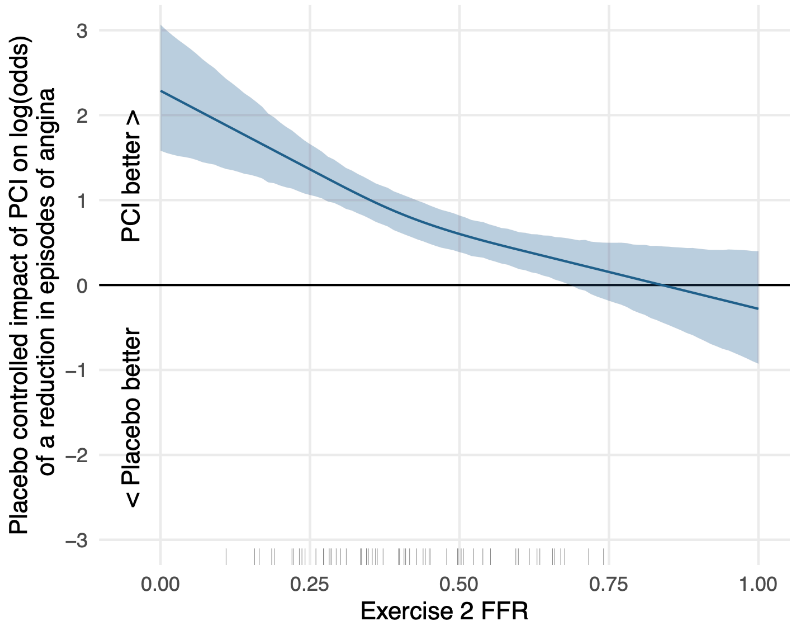

Regression model and coefficients for FFR$_{angina}$ (at low-exercise intensity)

Bayesian Constrained Partial Proportional Odds Ordinal Logistic Model

Dirichlet Priors With Concentration Parameter 0.392 for Intercepts

```
blrm(formula = symptom_frequency_num ~ rcs(symptom_frequency_num_lag1,
    c(1, 3, 5)) + rcs(day_num_m2, 3) + rcs(ex2_angina_ffr, 3) +
    rcs(symptom_frequency_num_pre_mean, 4), ppo = ~day_num_m2,
    cppo = function(y) y, data = main_analysis_d, pcontrast = pcon,
    iter = 5000, chains = 4, refresh = 100, progress = file.path(output_dir,
        "res1.progress.txt"), loo = FALSE, ppairs = NULL, method = "sampling",
    file = file.path(output_dir, "res1.blrm.rds"))
```

Frequencies of Responses

```
   0    1    2    3    4
4954  529  124   27    9
```

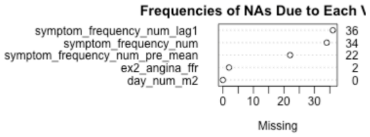

	Mixed Calibration/ Discrimination Indexes	Discrimination Indexes	Rank Discrim. Indexes
Obs 5643	B 0.076 [0.076, 0.077]	g 1.044 [0.95, 1.122]	C 0.782 [0.772, 0.792]
Draws 10000		g_p 0.126 [0.119, 0.134]	D_{xy} 0.564 [0.544, 0.584]
Chains 4		EV 0.277 [0.251, 0.304]	
Time 212.4s		v 1.284 [1.106, 1.447]	
p 9		vp 0.03 [0.027, 0.032]	

	Mean β	Median β	S.E.	Lower	Upper	Pr(β>0)	Symmetry
y≥1	-3.6202	-3.6142	0.4177	-4.4071	-2.7701	0.0000	0.97
y≥2	-5.3724	-5.3630	0.4327	-6.2556	-4.5638	0.0000	0.97
y≥3	-6.7643	-6.7566	0.4747	-7.6522	-5.7880	0.0000	0.96
y≥4	-7.9719	-7.9558	0.5693	-9.0512	-6.8098	0.0000	0.96
symptom_frequency_num_lag1	2.0153	2.0155	0.0836	1.8497	2.1740	1.0000	0.99
symptom_frequency_num_lag1'	-2.4627	-2.4582	0.5469	-3.5256	-1.4048	0.0000	0.95
day_num_m2	-0.0131	-0.0132	0.0035	-0.0202	-0.0064	0.0001	1.02
day_num_m2'	0.0224	0.0224	0.0034	0.0157	0.0290	1.0000	1.00
ex2_angina_ffr	3.7010	3.6857	1.0508	1.6516	5.7511	0.9999	1.04
ex2_angina_ffr'	-1.8141	-1.8031	1.5067	-4.9863	0.9909	0.1104	0.98
symptom_frequency_num_pre_mean	-0.4806	-0.4844	0.3612	-1.1786	0.2274	0.0876	1.01
symptom_frequency_num_pre_mean'	2.8964	2.8980	1.6955	-0.3354	6.2424	0.9566	0.99
symptom_frequency_num_pre_mean''	-6.0963	-6.1095	3.7369	-13.2856	1.2111	0.0496	0.99
day_num_m2 x f(y)	-0.0056	-0.0056	0.0014	-0.0084	-0.0030	0.0001	0.98

Contrasts Given Priors

```
[1] list(c1 = list(ex2_angina_ffr = 0.36), c2 = list(ex2_angina_ffr = 0.55),
[2]     contrast = expression(c1 - c2), sd = 0.842807127883599)
```

Supplementary figure S21: coefficient density plots: FFR_{angina} (at low-exercise intensity)

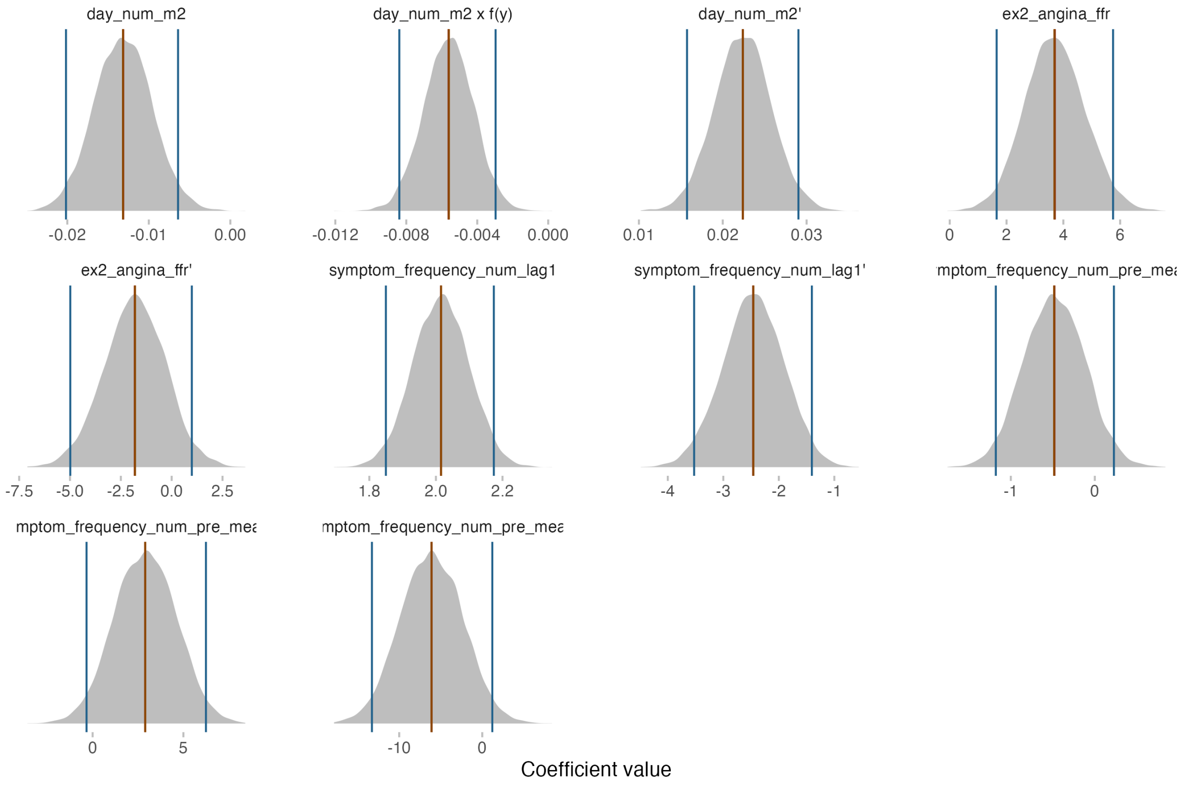

Supplementary figure S22: chain plot of MCMC draws for FFR_{angina} (at low-exercise intensity)

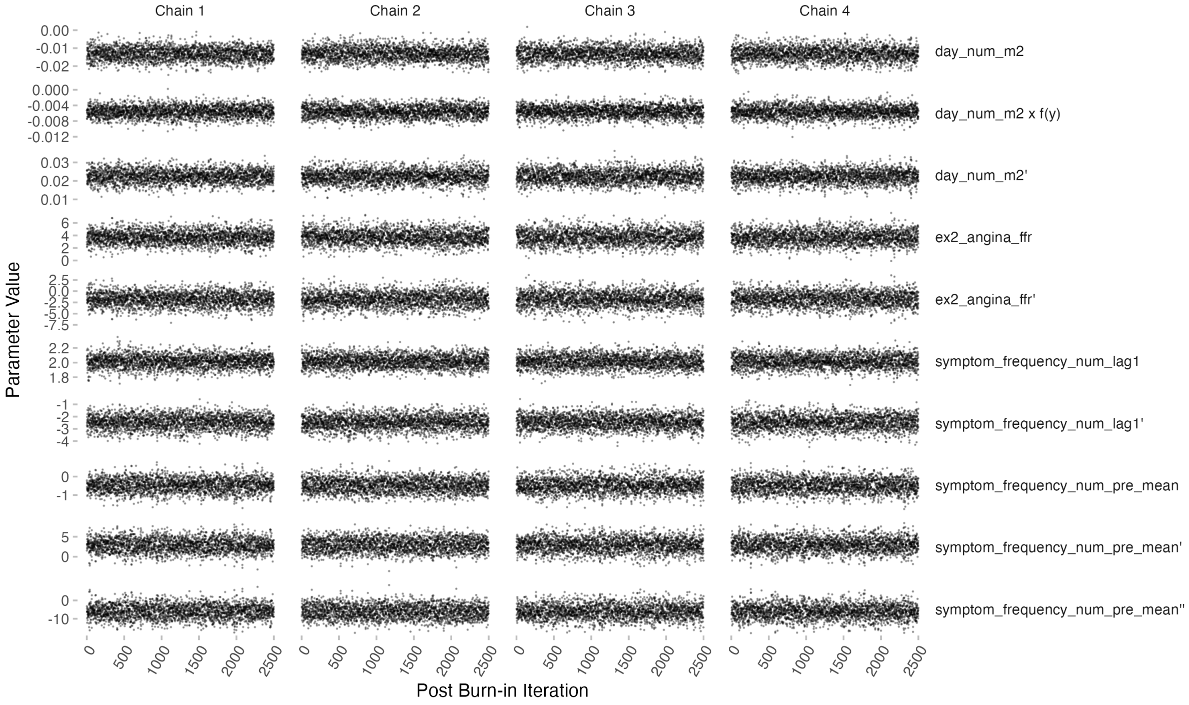

Supplemental Figure S23: RFR$_{angina}$ (at low-intensity exercise) and angina symptom score

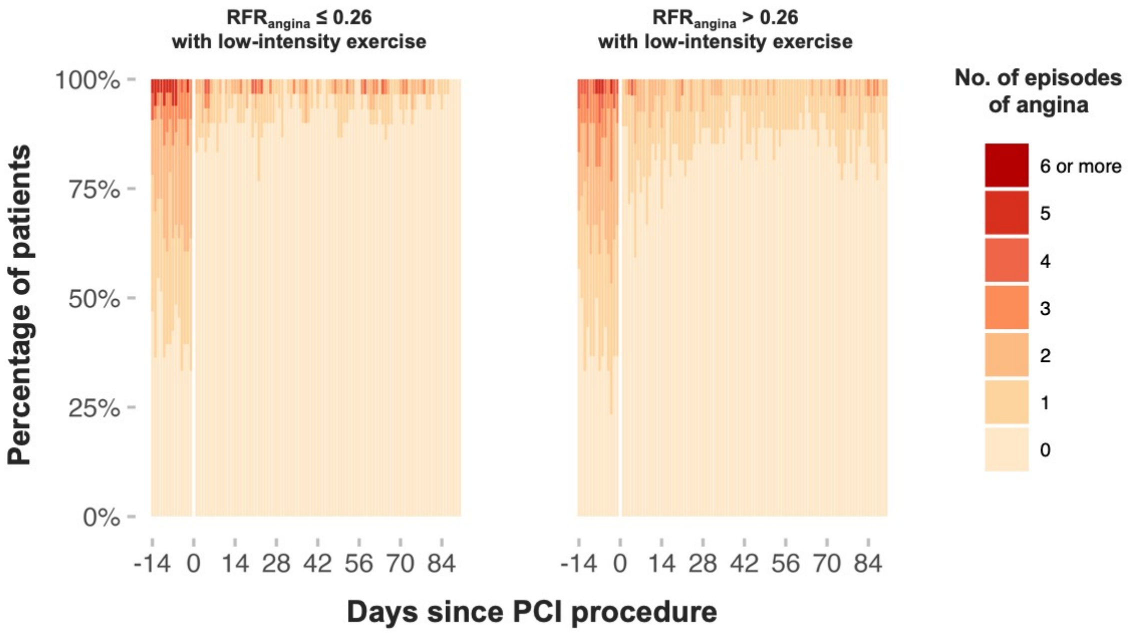

Supplemental Figure S24: RFR$_{angina}$ (at low-intensity exercise) and placebo-controlled impact of PCI

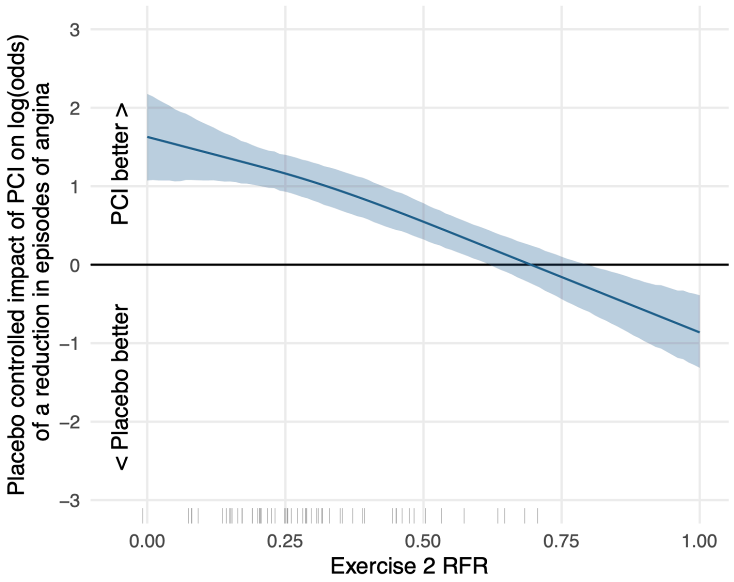

Regression model and coefficients for RFR_{angina} (at low-exercise intensity)

Bayesian Constrained Partial Proportional Odds Ordinal Logistic Model

Dirichlet Priors With Concentration Parameter 0.392 for Intercepts

```
blrm(formula = symptom_frequency_num ~ rcs(symptom_frequency_num_lag1,
    c(1, 3, 5)) + rcs(day_num_m2, 3) + rcs(ex2_angina_rfr, 3) +
    rcs(symptom_frequency_num_pre_mean, 4), ppo = ~day_num_m2,
    cppo = function(y) y, data = main_analysis_d, pcontrast = pcon,
    iter = 5000, chains = 4, refresh = 100, progress = file.path(output_dir,
        "res1.progress.txt"), loo = FALSE, ppairs = NULL, method = "sampling",
    file = file.path(output_dir, "res1.blrm.rds"))
```

Frequencies of Responses

```
   0    1    2    3    4
4954  529  124   27    9
```

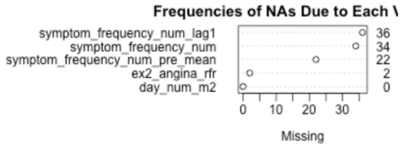

	Mixed Calibration/ Discrimination Indexes	Discrimination Indexes	Rank Discrim. Indexes
Obs 5643	B 0.075 [0.075, 0.076]	g 1.053 [0.969, 1.14]	C 0.787 [0.776, 0.796]
Draws 10000		g_p 0.128 [0.121, 0.136]	D_{xy} 0.574 [0.552, 0.591]
Chains 4		EV 0.285 [0.259, 0.311]	
Time 189.9s		v 1.314 [1.157, 1.481]	
p 9		vp 0.03 [0.028, 0.034]	

	Mean β	Median β	S.E.	Lower	Upper	Pr(β>0)	Symmetry
y≥1	-2.6876	-2.6807	0.3440	-3.3590	-2.0074	0.0000	0.97
y≥2	-4.4483	-4.4406	0.3602	-5.1444	-3.7353	0.0000	0.98
y≥3	-5.8311	-5.8282	0.4075	-6.6501	-5.0451	0.0000	0.98
y≥4	-7.0343	-7.0272	0.5220	-8.0530	-5.9892	0.0000	0.97
symptom_frequency_num_lag1	1.9467	1.9465	0.0837	1.7828	2.1099	1.0000	1.00
symptom_frequency_num_lag1'	-2.1540	-2.1406	0.5598	-3.2569	-1.0752	0.0000	0.96
day_num_m2	-0.0128	-0.0129	0.0035	-0.0198	-0.0060	0.0002	1.02
day_num_m2'	0.0214	0.0214	0.0033	0.0153	0.0282	1.0000	1.00
ex2_angina_rfr	1.8468	1.8435	1.0832	-0.2168	3.9636	0.9555	1.00
ex2_angina_rfr'	1.2728	1.2726	1.9324	-2.5254	4.9952	0.7450	1.01
symptom_frequency_num_pre_mean	-0.7458	-0.7516	0.3702	-1.4622	-0.0119	0.0241	1.04
symptom_frequency_num_pre_mean'	3.8213	3.8363	1.7238	0.4326	7.2042	0.9856	0.97
symptom_frequency_num_pre_mean''	-8.0555	-8.0849	3.7846	-15.2207	-0.3404	0.0184	1.03
day_num_m2 x f(y)	-0.0057	-0.0057	0.0014	-0.0085	-0.0030	0.0000	0.95

Contrasts Given Priors

```
[1] list(c1 = list(ex2_angina_rfr = 0.36), c2 = list(ex2_angina_rfr = 0.55),
[2]     contrast = expression(c1 - c2), sd = 0.842807127883599)
```

Supplementary figure S35: coefficient density plots: RFR_{angina} (at low-exercise intensity)

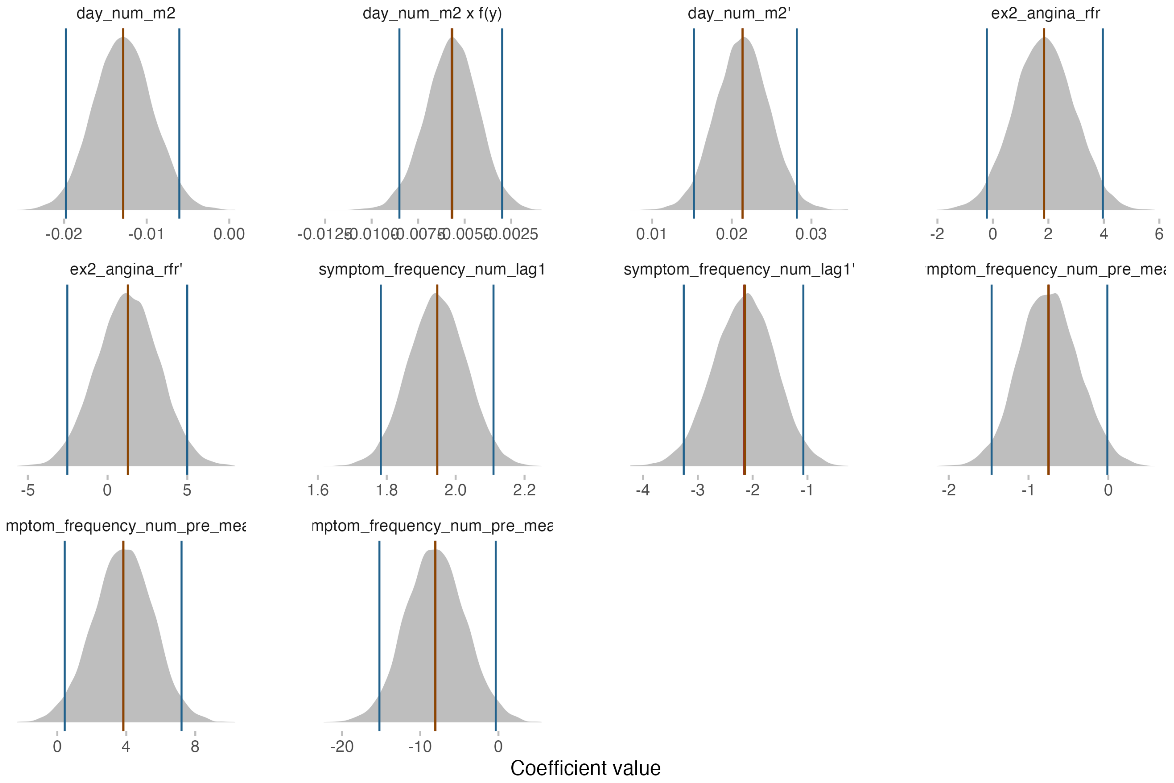

Supplementary figure S26: chain plot of MCMC draws for RFR_{angina} (at low-exercise intensity)

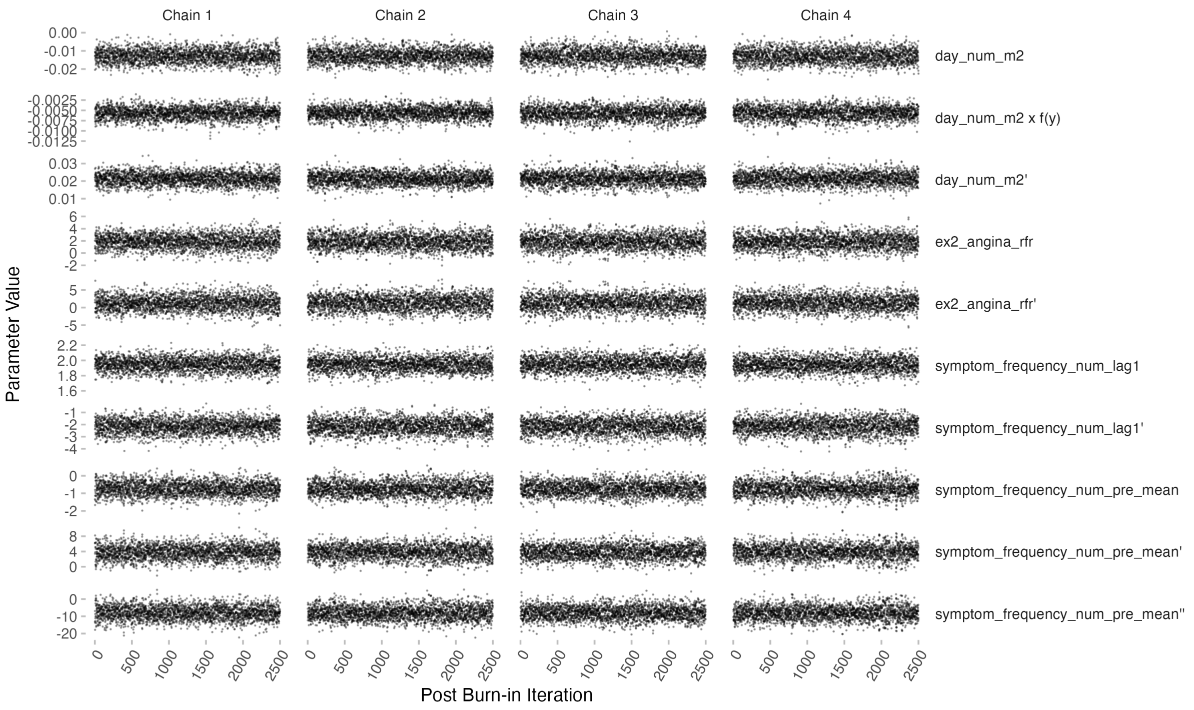

Supplemental Figure S27: FFR$_{angina}$ (at high-intensity exercise) and angina symptom score

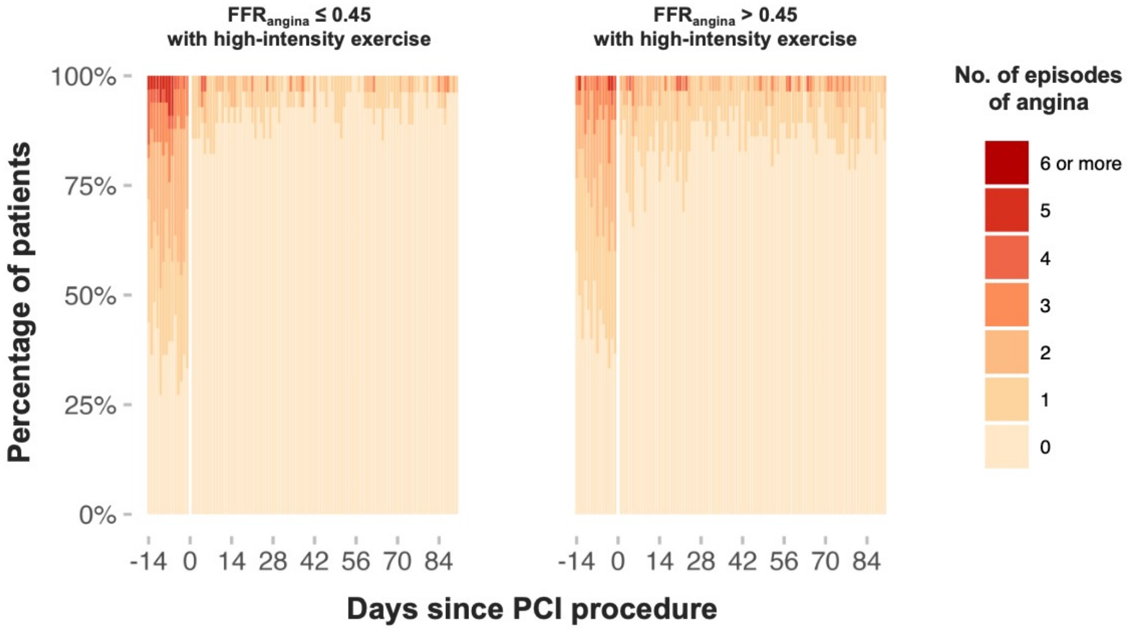

Supplemental Figure S28: FFR$_{angina}$ (at high-intensity exercise) and placebo-controlled impact of PCI

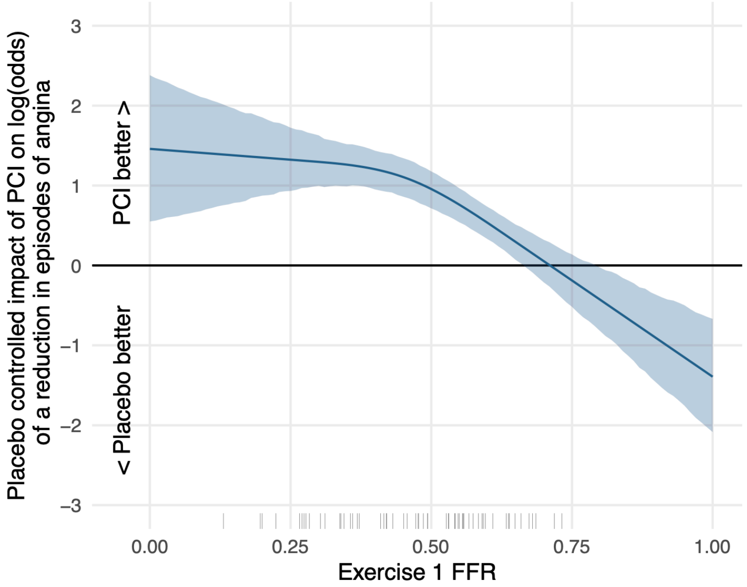

Regression model and coefficients for FFR_{angina} (at high-intensity exercise)

Bayesian Constrained Partial Proportional Odds Ordinal Logistic Model

Dirichlet Priors With Concentration Parameter 0.392 for Intercepts

```
blrm(formula = symptom_frequency_num ~ rcs(symptom_frequency_num_lag1,
    c(1, 3, 5)) + rcs(day_num_m2, 3) + rcs(ex1_angina_ffr, 3) +
    rcs(symptom_frequency_num_pre_mean, 4), ppo = ~day_num_m2,
    cppo = function(y) y, data = main_analysis_d, pcontrast = pcon,
    iter = 5000, chains = 4, refresh = 100, progress = file.path(output_dir,
        "res1.progress.txt"), loo = FALSE, ppairs = NULL, method = "sampling",
    file = file.path(output_dir, "res1.blrm.rds"))
```

Frequencies of Responses

```
   0    1    2    3    4
4956  529  124   27    9
```

Frequencies of Missing Values Due to Each Variable

```
         symptom_frequency_num     symptom_frequency_num_lag1
                            34                             36
                    day_num_m2                  ex1_angina_ffr
                             0                              0
symptom_frequency_num_pre_mean
                            22
```

	Mixed Calibration/ Discrimination Indexes	Discrimination Indexes	Rank Discrim. Indexes
Obs 5645	B 0.076 [0.075, 0.076]	g 1.033 [0.964, 1.115]	C 0.774 [0.763, 0.782]
Draws 10000		g_p 0.126 [0.12, 0.133]	D_{xy} 0.548 [0.527, 0.565]
Chains 4		EV 0.281 [0.256, 0.309]	
Time 74.2s		v 1.281 [1.145, 1.449]	
p 9		vp 0.03 [0.027, 0.033]	

	Mean β	Median β	S.E.	Lower	Upper	Pr(β>0)	Symmetry
y≥1	-2.7186	-2.7103	0.4686	-3.6392	-1.7875	0.0000	0.96
y≥2	-4.4852	-4.4778	0.4825	-5.4312	-3.5342	0.0000	0.95
y≥3	-5.8783	-5.8758	0.5214	-6.8944	-4.8592	0.0000	0.96
y≥4	-7.0809	-7.0765	0.6093	-8.2829	-5.9048	0.0000	0.96
symptom_frequency_num_lag1	1.9927	1.9925	0.0867	1.8198	2.1575	1.0000	1.00
symptom_frequency_num_lag1'	-2.5852	-2.5790	0.5641	-3.6880	-1.5023	0.0000	0.97
day_num_m2	-0.0123	-0.0123	0.0036	-0.0193	-0.0051	0.0006	1.00
day_num_m2'	0.0208	0.0208	0.0034	0.0141	0.0275	1.0000	1.02
ex1_angina_ffr	0.5424	0.5312	1.1767	-1.7441	2.8612	0.6760	1.04
ex1_angina_ffr'	2.8512	2.8651	1.2445	0.4934	5.3680	0.9861	0.98
symptom_frequency_num_pre_mean	-0.5524	-0.5536	0.3527	-1.2222	0.1469	0.0573	1.02
symptom_frequency_num_pre_mean'	3.1849	3.1890	1.6406	0.1472	6.6016	0.9720	0.97
symptom_frequency_num_pre_mean''	-6.6299	-6.6329	3.6040	-13.8277	0.3214	0.0345	1.02
day_num_m2 x f(y)	-0.0057	-0.0057	0.0014	-0.0083	-0.0029	0.0000	0.98

Contrasts Given Priors

```
[1] list(c1 = list(ex1_angina_ffr = 0.36), c2 = list(ex1_angina_ffr = 0.55),
[2]     contrast = expression(c1 - c2), sd = 0.842807127883599)
```

Supplementary figure S30: coefficient density plots: FFR_{angina} (at high-intensity exercise)

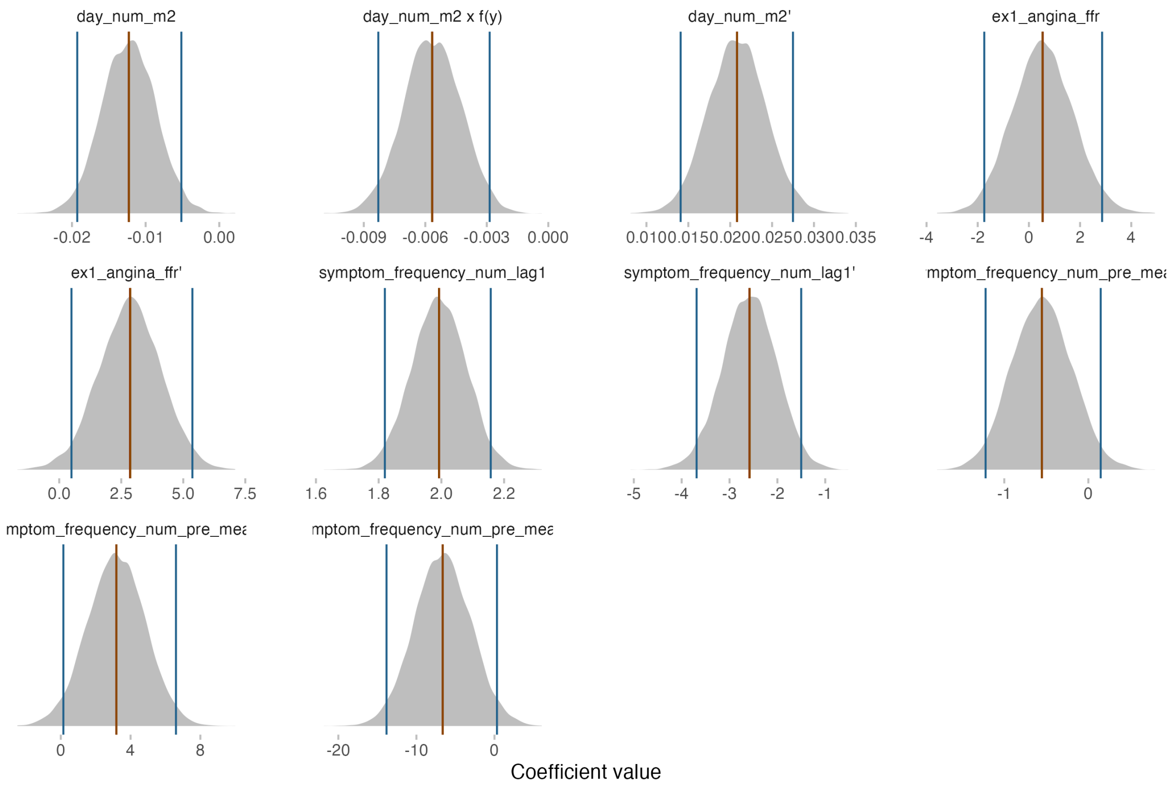

Supplementary figure S31: chain plot of MCMC draws for FFR_{angina} (at high-intensity exercise)

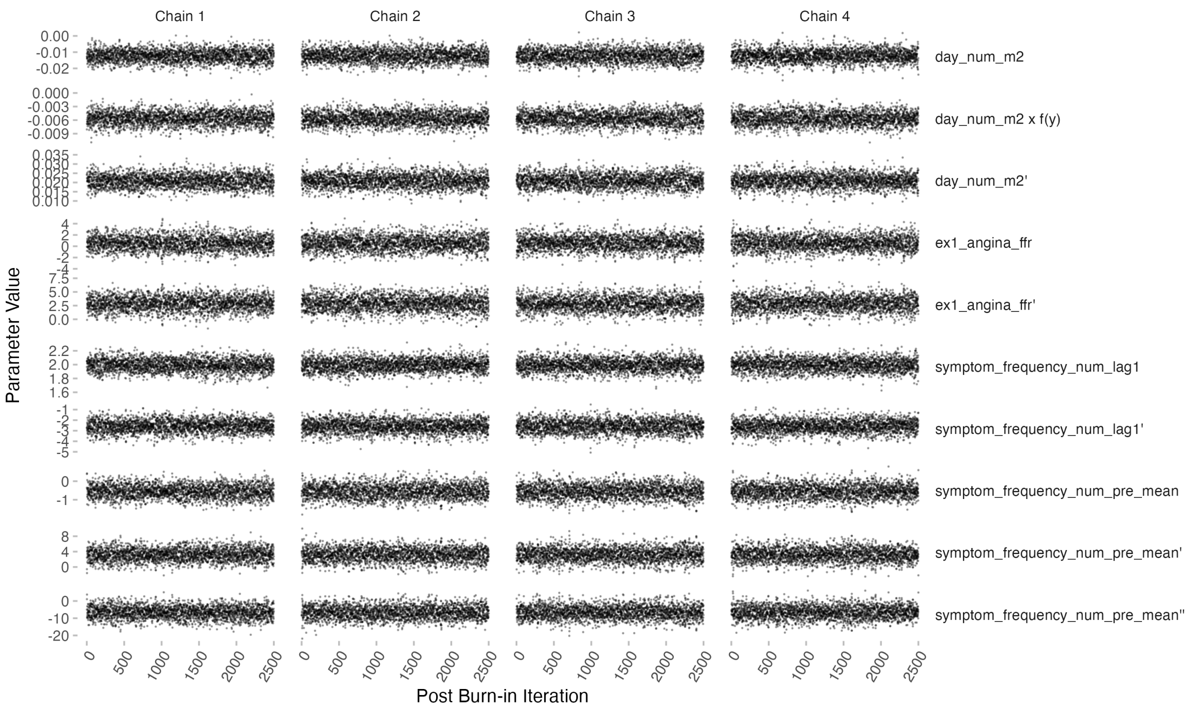

Supplemental Figure S32: RFR$_{angina}$ (at high-intensity exercise) and angina symptom score

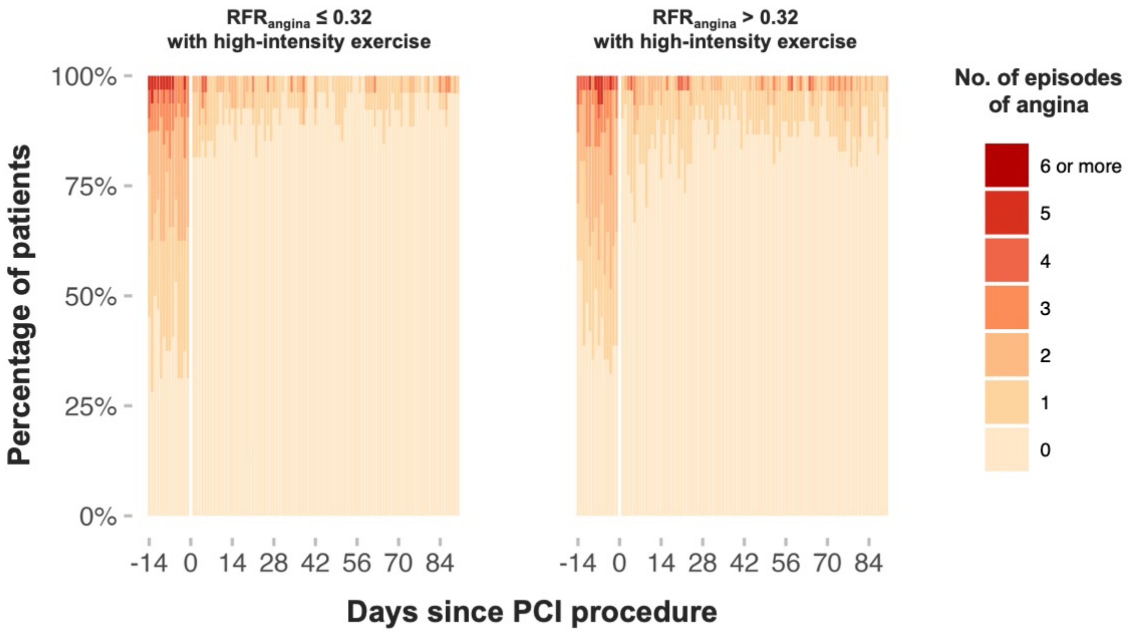

Supplemental Figure S33: RFR$_{angina}$ (at high-intensity exercise) and placebo-controlled impact of PCI

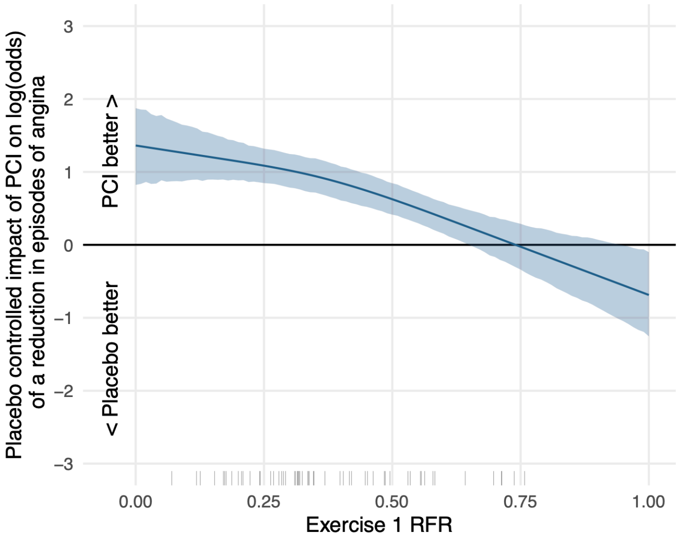

Regression model and coefficients for RFR_{angina} (at high-intensity exercise)

Bayesian Constrained Partial Proportional Odds Ordinal Logistic Model

Dirichlet Priors With Concentration Parameter 0.392 for Intercepts

```
blrm(formula = symptom_frequency_num ~ rcs(symptom_frequency_num_lag1,
    c(1, 3, 5)) + rcs(day_num_m2, 3) + rcs(ex1_angina_rfr, 3) +
    rcs(symptom_frequency_num_pre_mean, 4), ppo = ~day_num_m2,
    cppo = function(y) y, data = main_analysis_d, pcontrast = pcon,
    iter = 5000, chains = 4, refresh = 100, progress = file.path(output_dir,
        "res1.progress.txt"), loo = FALSE, ppairs = NULL, method = "sampling",
    file = file.path(output_dir, "res1.blrm.rds"))
```

Frequencies of Responses

```
   0    1    2    3    4
4956  529  124   27    9
```

Frequencies of Missing Values Due to Each Variable

```
         symptom_frequency_num     symptom_frequency_num_lag1
                            34                             36
                    day_num_m2                  ex1_angina_rfr
                             0                              0
symptom_frequency_num_pre_mean
                            22
```

	Mixed Calibration/ Discrimination Indexes	Discrimination Indexes	Rank Discrim. Indexes
Obs 5645	B 0.076 [0.075, 0.077]	g 0.988 [0.912, 1.062]	C 0.765 [0.752, 0.779]
Draws 10000		g_p 0.124 [0.116, 0.13]	D_{xy} 0.531 [0.505, 0.558]
Chains 4		EV 0.278 [0.255, 0.304]	
Time 262.1s		v 1.229 [1.094, 1.381]	
p 9		vp 0.03 [0.027, 0.032]	

	Mean β	Median β	S.E.	Lower	Upper	Pr(β>0)	Symmetry
y≥1	-2.6497	-2.6495	0.3173	-3.3042	-2.0598	0.0000	0.96
y≥2	-4.4135	-4.4100	0.3354	-5.0583	-3.7396	0.0000	0.96
y≥3	-5.8112	-5.8063	0.3879	-6.5770	-5.0509	0.0000	0.99
y≥4	-7.0196	-7.0073	0.5045	-8.0133	-6.0486	0.0000	0.95
symptom_frequency_num_lag1	2.0211	2.0216	0.0851	1.8578	2.1892	1.0000	0.96
symptom_frequency_num_lag1'	-2.5904	-2.5841	0.5727	-3.6824	-1.4566	0.0000	0.97
day_num_m2	-0.0121	-0.0122	0.0036	-0.0194	-0.0052	0.0005	1.01
day_num_m2'	0.0201	0.0201	0.0034	0.0133	0.0267	1.0000	0.99
ex1_angina_rfr	1.0908	1.0831	0.8803	-0.5700	2.9026	0.8951	1.02
ex1_angina_rfr'	1.3091	1.3055	1.1691	-0.9788	3.6379	0.8711	1.01
symptom_frequency_num_pre_mean	-0.4830	-0.4812	0.3443	-1.1834	0.1685	0.0814	1.03
symptom_frequency_num_pre_mean'	2.5837	2.5831	1.6047	-0.6211	5.6839	0.9481	1.00
symptom_frequency_num_pre_mean''	-5.3268	-5.2908	3.5370	-12.1441	1.8426	0.0643	0.99
day_num_m2 x f(y)	-0.0056	-0.0055	0.0014	-0.0084	-0.0029	0.0000	0.95

Contrasts Given Priors

```
[1] list(c1 = list(ex1_angina_rfr = 0.36), c2 = list(ex1_angina_rfr = 0.55),
[2]     contrast = expression(c1 - c2), sd = 0.842807127883599)
```

Supplementary figure S34: coefficient density plots: RFR_{angina} (at high-intensity exercise)

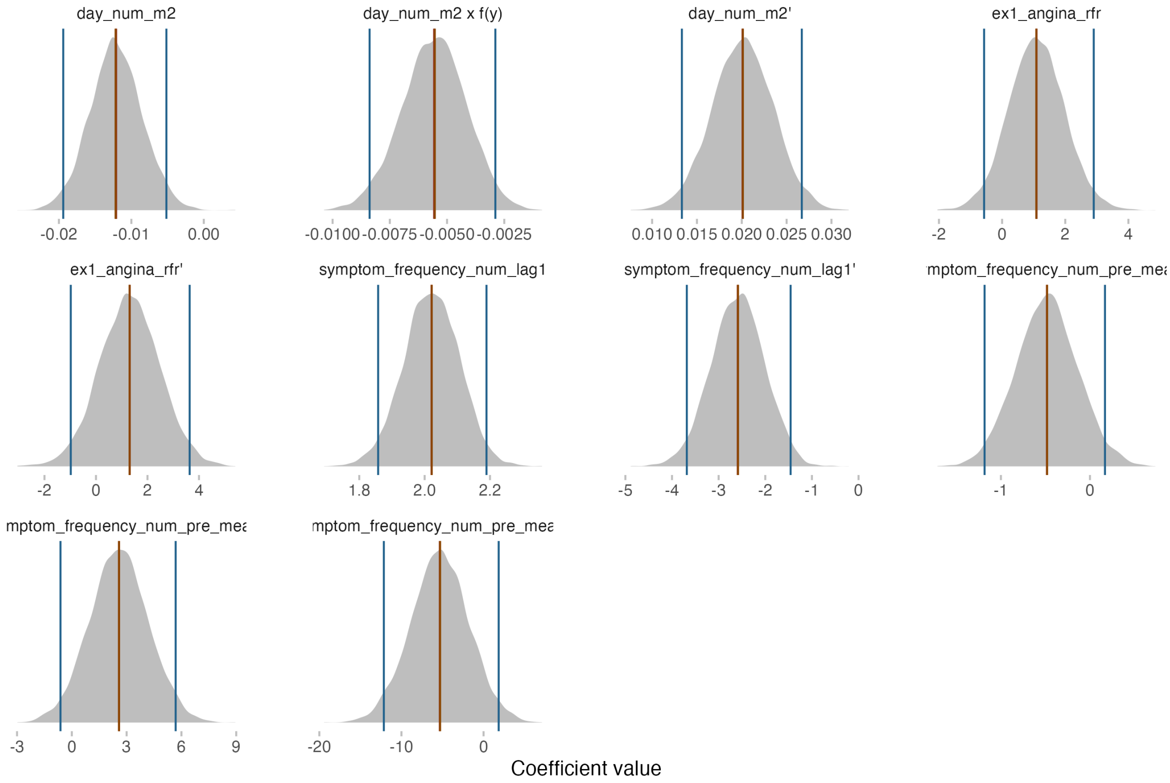

Supplementary figure S35: chain plot of MCMC draws for RFR_{angina} (at high-intensity exercise)

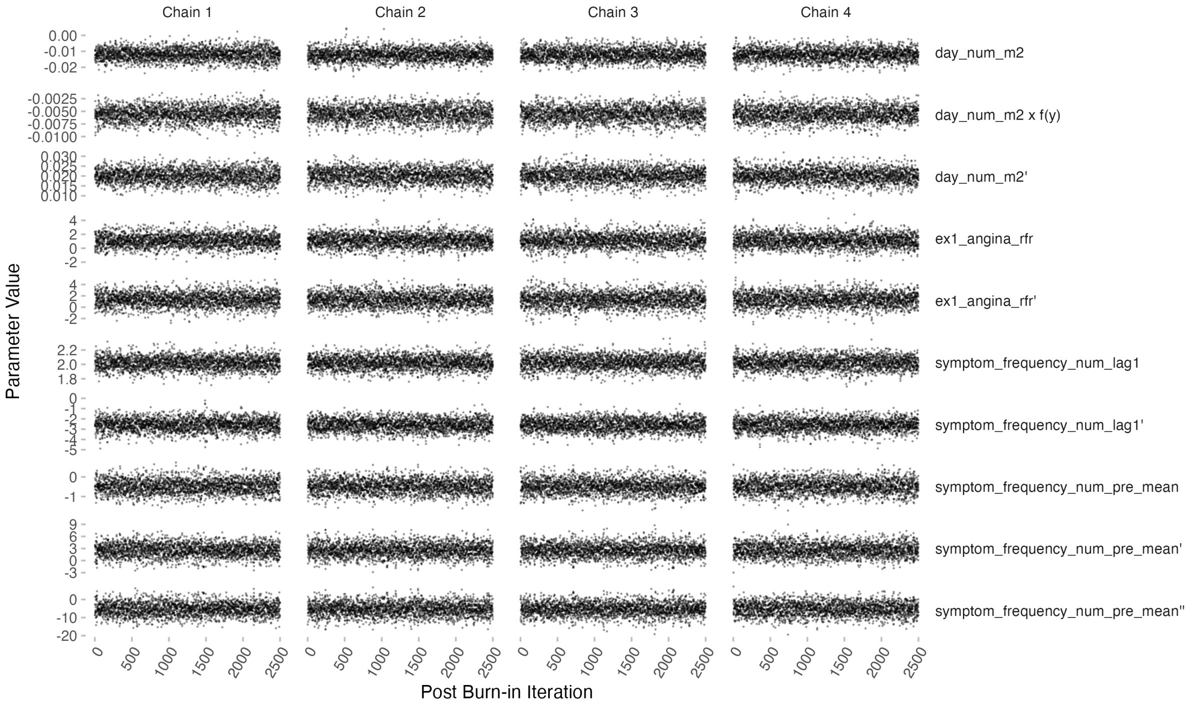

Baseline symptom questionnaires

Canadian Cardiovascular Society (CCS) Class

Total of 65 patients analyzed

Supplemental Table S10: CCS class and angina thresholds

CCS Class	Number of patients (%)
**1**	4 (6.2)
**2**	30 (46.2)
**3**	29 (44.6)
**4**	2 (3.1)

Statistical analysis comparing CCS class and angina threshold

Angina threshold	Logistic Regression P-value
FFR_{angina} at rest	0.362
RFR_{angina} at rest	0.371
FFR_{angina} at low-intensity exercise	0.202
RFR_{angina} at low-intensity exercise	0.262
FFR_{angina} at high-intensity exercise	0.209
RFR_{angina} at high-intensity exercise	0.184

Rose angina questionnaire

Total of 65 patients analyzed

Supplemental Table S11: Rose Angina status and angina thresholds

Rose Angina status	Number of patients (%)
Positive	51
Negative	14

Statistical analysis comparing Rose angina status and angina thresholds

Angina threshold	Logistic Regression P-value
FFR_{angina} at rest	0.4194
RFR_{angina} at rest	0.477
FFR_{angina} at low-intensity exercise	0.536
RFR_{angina} at low-intensity exercise	0.697
FFR_{angina} at high-intensity exercise	0.794
RFR_{angina} at high-intensity exercise	0.525

Seattle angina questionnaire (SAQ)

Total of 65 patients analyzed

Supplemental Table S12: SAQ Physical limitation and angina thresholds

Statistical analysis for SAQ physical limitations

Angina threshold	Logistic Regression P-value
FFR_{angina} at rest	0.6211
RFR_{angina} at rest	0.2399
FFR_{angina} at low-intensity exercise	0.4773
RFR_{angina} at low-intensity exercise	0.6144
FFR_{angina} at high-intensity exercise	0.2705
RFR_{angina} at high-intensity exercise	0.4102

Supplemental Table S13: SAQ Angina Frequency and angina thresholds

Statistical analysis for SAQ Angina Frequency

Angina threshold	Logistic Regression P-value
FFR_{angina} at rest	0.5826
RFR_{angina} at rest	0.414
FFR_{angina} at low-intensity exercise	0.2470
RFR_{angina} at low-intensity exercise	0.2954
FFR_{angina} at high-intensity exercise	0.4993
RFR_{angina} at high-intensity exercise	0.2558

Supplemental Table S14: SAQ Quality of life and angina thresholds

Statistical analysis for SAQ Quality of Life

Angina threshold	Logistic Regression P-value
FFR_{angina} at rest	0.5798
RFR_{angina} at rest	0.3826
FFR_{angina} at low-intensity exercise	0.8196
RFR_{angina} at low-intensity exercise	0.8264
FFR_{angina} at high-intensity exercise	0.7221
RFR_{angina} at high-intensity exercise	0.5649

Supplemental Table S15: SAQ stability and angina thresholds

Statistical analysis for SAQ Angina Stability

Angina threshold	Logistic Regression P-value
FFR_{angina} at rest	0.3973
RFR_{angina} at rest	0.4666
FFR_{angina} at low-intensity exercise	0.2398
RFR_{angina} at low-intensity exercise	0.2166
FFR_{angina} at high-intensity exercise	0.690
RFR_{angina} at high-intensity exercise	0.6864

Supplemental Table S16: SAQ treatment satisfaction and angina thresholds

Statistical analysis Treatment Satisfaction

Angina threshold	Logistic Regression P-value
FFR_{angina} at rest	0.3304
RFR_{angina} at rest	0.3126
FFR_{angina} at low-intensity exercise	0.1323
RFR_{angina} at low-intensity exercise	0.1953
FFR_{angina} at high-intensity exercise	0.3697
RFR_{angina} at high-intensity exercise	0.4151

Pain-sensitivity Questionnaire (PSQ)

Total of 65 patients analyzed

Supplemental Table S17: PSQ and angina thresholds

Median score (IQR)	Mean score (SD)
72 (57-98)	76.67 (25.01)

Statistical analysis for PSQ

Angina threshold	Logistic Regression P-value
FFR_{angina} at rest	0.84473
RFR_{angina} at rest	0.6924
FFR_{angina} at low-intensity exercise	0.91034
RFR_{angina} at low-intensity exercise	0.75742
FFR_{angina} at high-intensity exercise	0.353
RFR_{angina} at high-intensity exercise	0.43896

Exercise data analysis

Supplemental Figure S36: Rate–pressure product (RPP) across all exercise stages

This figure shows a progressive rise in RPP from rest to low- and high-intensity supine exercise, illustrated by individual patient trajectories (grey lines) and the cohort median at each stage (black squares). The figure shows RPP increased significantly across all exercise stages (P <0.001 for all pairwise comparisons).

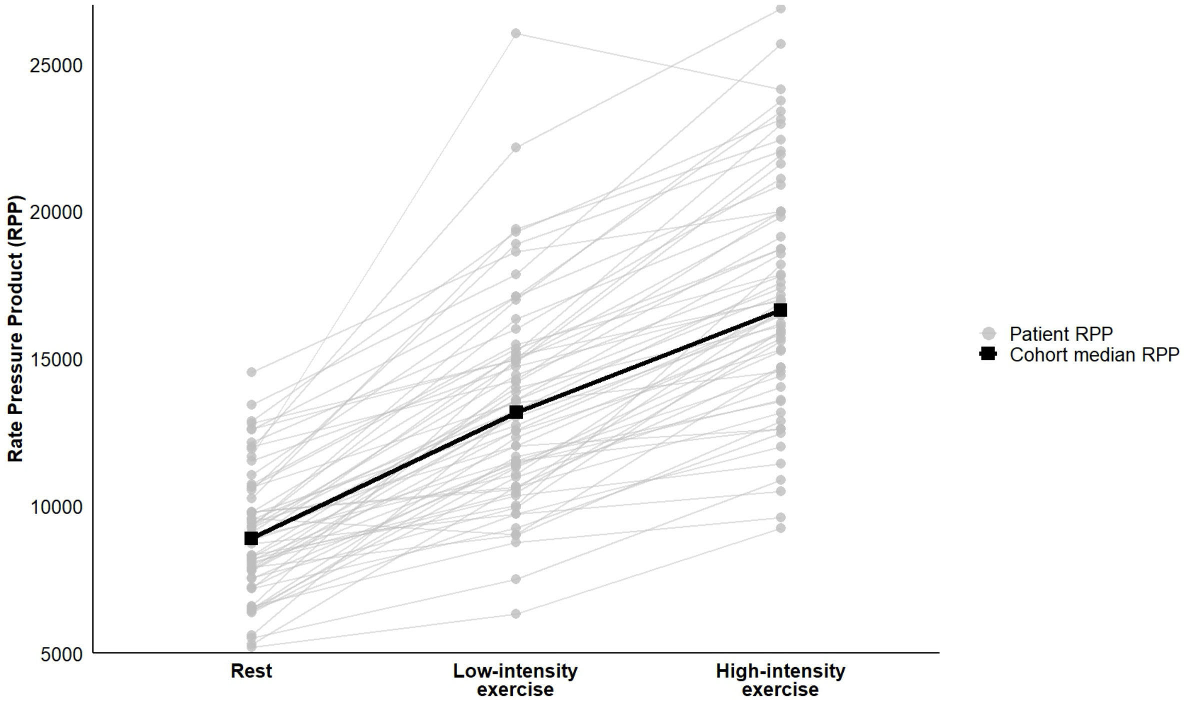

Supplemental Figure S37: Systolic blood pressure (SBP) and the angina thresholds

Figure **(a)** shows the relationship between SBP and FFR_{angina}, and **(b)** shows the corresponding relationship between SBP and RFR_{angina}. Each point represents an individual angina threshold measured at rest (red), low-intensity exercise (yellow), or high-intensity exercise (blue), with larger circles indicating the median for each stage. Both figures demonstrate a progressive rise in FFR_{angina} and RFR_{angina} with increasing systolic blood pressure. SBP also increased significantly across all pairwise comparisons (Rest vs Low P =0.008, Rest vs high P <0.001, Low vs high P =0.018).

Figure 37 (a)

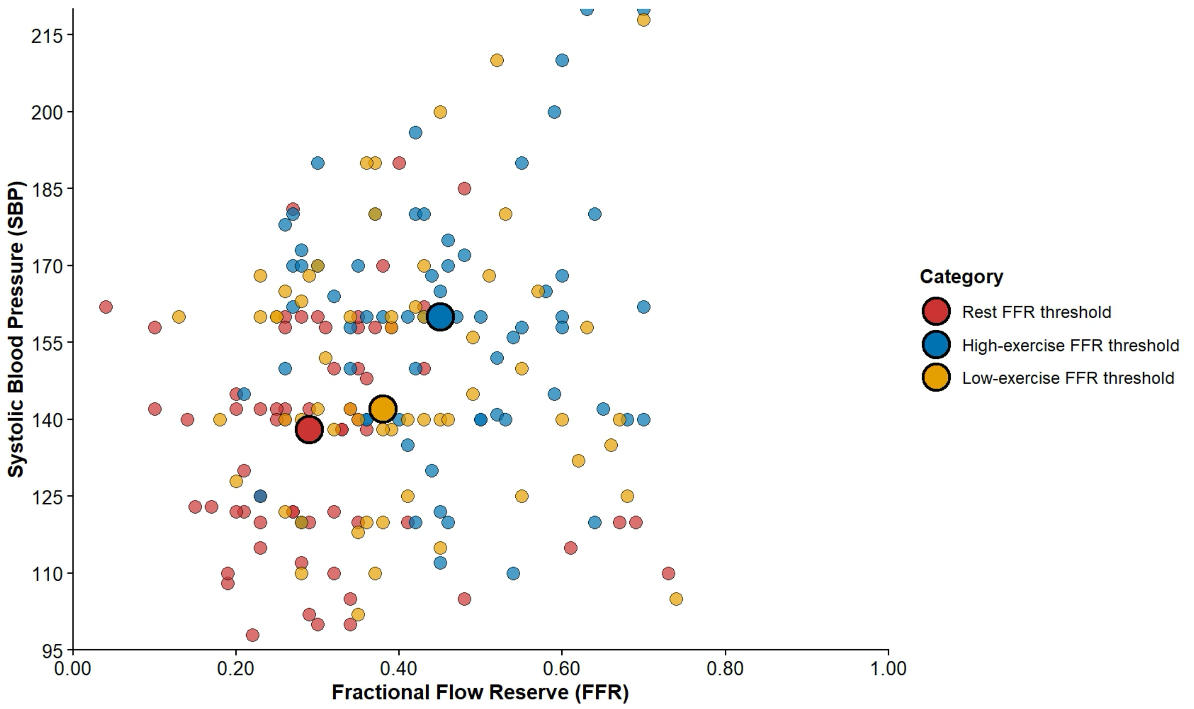

Figure 37 (b)

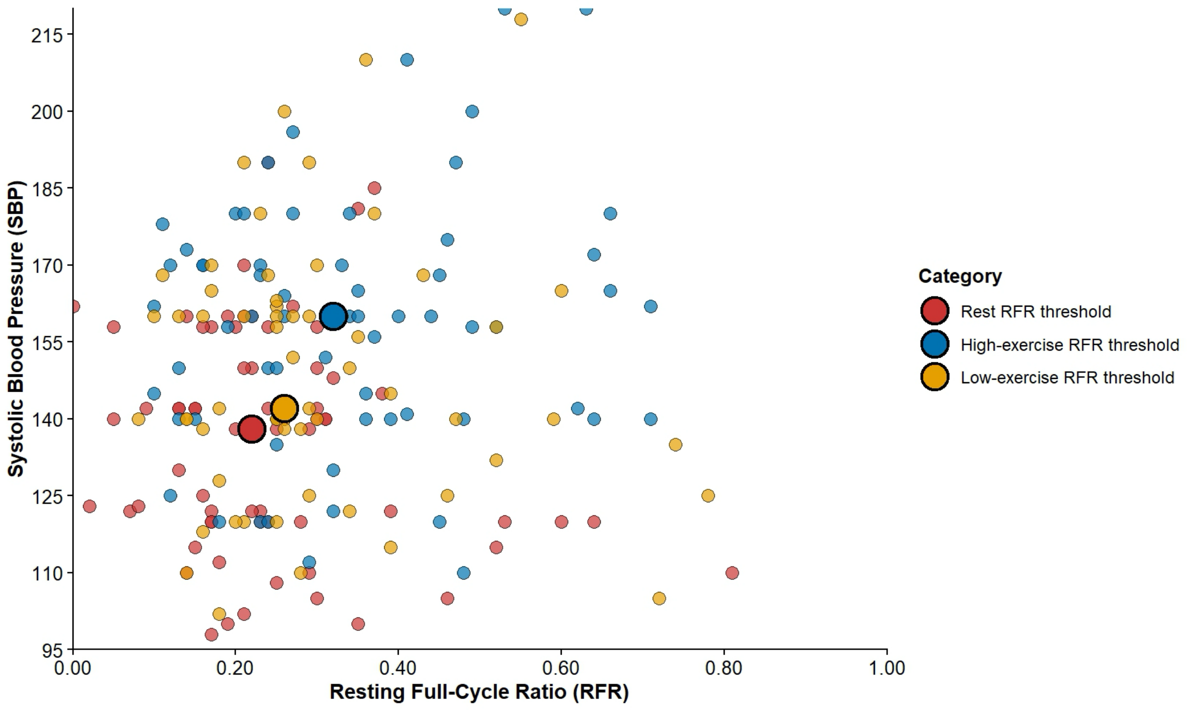

Antianginal medication record

Supplemental Table S18: Baseline antianginal medication use

Number of antianginals prior to enrolment	Patients (n = 65)
0	10 (15.4%)
1	29 (44.6%)
2	23 (35.4%)
3	1 (1.5%)
4	2 (3.1%)

Supplemental Table S19: Type and mean daily dose of antianginal medications used

Medication class	Patients	Antianginal units mean (SD)
Betablocker	34	0.73 (0.60)
CCB	33	2.58 (1.07)
ISMN	14	1.31 (0.55)
Nicorandil	3	3.67 (5.48)
Ranolazine	2	0.73 (0.85)

* Note: numbers do not equal 65 as some patients were not on any antianginal medications while others were on two or more.

Supplemental Table S20: Antianginal medication usage during the study

One participant remained on bisoprolol throughout the study for rate-controlled atrial fibrillation; this was discontinued 48 hours before the research procedure to allow adequate pharmacological washout and ensure accurate symptom reporting.

Number of antianginals during the study	Type and dose	Duration
1	Bisoprolol 5mg	Taken for rate-controlled atrial fibrillation. Taken during the whole study apart from 48 hours prior to PCI procedure to allow adequate washout

Completeness of data collection

Supplemental Table S21: Data completeness

**Data**	**Number of patient (%)**
Baseline	
Demographics	65 (100)
Pre-PCI physiology	
FFR	65 (100)
RFR	65 (100)
CFR	51 (78.5)
IMR	51 (78.5)
CVP	65 (100)
Collateral Flow	
CFI	65 (100)
PCI	
PCI data set	65 (100)
Post-PCI physiology	
FFR	65 (100)
RFR	65 (100)
CFR	60 (92.3)
IMR	60 (92.3)
Rest Angina threshold	
FFR_{angina}	65 (100)
RFR_{angina}	65 (100)
Low-intensity exercise angina threshold	
FFR_{angina}	61 (93.8)
RFR_{angina}	61 (93.8)
High-intensity exercise angina threshold	
FFR_{angina}	65 (100)
RFR_{angina}	65 (100)

Values are n (%) unless otherwise indicated

Supplementary Figure S38: Consort checklist

CONSORT 2010 checklist of information to include when reporting a randomised trial*

Section/Topic	Item No	Checklist item	Reported on page No
**Title and abstract**			
	1a	Identification as a randomised trial in the title	1
	1b	Structured summary of trial design, methods, results, and conclusions (for specific guidance see CONSORT for abstracts)	4
**Introduction**			
Background and objectives	2a	Scientific background and explanation of rationale	7
	2b	Specific objectives or hypotheses	8
**Methods**			
Trial design	3a	Description of trial design (such as parallel, factorial) including allocation ratio	9
	3b	Important changes to methods after trial commencement (such as eligibility criteria), with reasons	N/A
Participants	4a	Eligibility criteria for participants	9 and supplement
	4b	Settings and locations where the data were collected	9
Interventions	5	The interventions for each group with sufficient details to allow replication, including how and when they were actually administered	10-13
Outcomes	6a	Completely defined pre-specified primary and secondary outcome measures, including how and when they were assessed	11
	6b	Any changes to trial outcomes after the trial commenced, with reasons	N/A
Sample size	7a	How sample size was determined	Supplement
	7b	When applicable, explanation of any interim analyses and stopping guidelines	N/A
Randomisation:			
Sequence generation	8a	Method used to generate the random allocation sequence	13
	8b	Type of randomisation; details of any restriction (such as blocking and block size)	Supplement
Allocation concealment mechanism	9	Mechanism used to implement the random allocation sequence (such as sequentially numbered containers), describing any steps taken to conceal the sequence until interventions were assigned	Supplement
Implementation	10	Who generated the random allocation sequence, who enrolled participants, and who assigned participants to interventions	13
Blinding	11a	If done, who was blinded after assignment to interventions (for example, participants, care providers, those	11-13

Section/Topic	Item No	Checklist item	Reported on page No
		assessing outcomes) and how	
	11b	If relevant, description of the similarity of interventions	N/A
Statistical methods	12a	Statistical methods used to compare groups for primary and secondary outcomes	13-14
	12b	Methods for additional analyses, such as subgroup analyses and adjusted analyses	13-14
**Results**			
Participant flow (a diagram is strongly recommended)	13a	For each group, the numbers of participants who were randomly assigned, received intended treatment, and were analysed for the primary outcome	N/A
	13b	For each group, losses and exclusions after randomisation, together with reasons	N/A
Recruitment	14a	Dates defining the periods of recruitment and follow-up	15
	14b	Why the trial ended or was stopped	N/A 29
Baseline data	15	A table showing baseline demographic and clinical characteristics for each group	
Numbers analysed	16	For each group, number of participants (denominator) included in each analysis and whether the analysis was by original assigned groups	Supplement
Outcomes and estimation	17a	For each primary and secondary outcome, results for each group, and the estimated effect size and its precision (such as 95% confidence interval)	31
	17b	For binary outcomes, presentation of both absolute and relative effect sizes is recommended	31
Ancillary analyses	18	Results of any other analyses performed, including subgroup analyses and adjusted analyses, distinguishing pre-specified from exploratory	31 and supplement
Harms	19	All important harms or unintended effects in each group (for specific guidance see CONSORT for harms)	19
**Discussion**			
Limitations	20	Trial limitations, addressing sources of potential bias, imprecision, and, if relevant, multiplicity of analyses	26
Generalisability	21	Generalisability (external validity, applicability) of the trial findings	24-25
Interpretation	22	Interpretation consistent with results, balancing benefits and harms, and considering other relevant evidence	20-25
**Other information**			
Registration	23	Registration number and name of trial registry	N/A
Protocol	24	Where the full trial protocol can be accessed, if available	Supplement
Funding	25	Sources of funding and other support (such as supply of drugs), role of funders	2

*We strongly recommend reading this statement in conjunction with the CONSORT 2010 Explanation and Elaboration for important clarifications on all the items. If relevant, we also recommend reading CONSORT extensions for cluster randomised trials, non-inferiority and equivalence trials, non-pharmacological treatments, herbal interventions, and pragmatic trials. Additional extensions are forthcoming: for those and for up to date references relevant to this checklist, see www.consort-statement.org.
